# Tumor necrosis factor inhibitors and janus kinase inhibitors in the treatment of cicatricial alopecia: A systematic review

**DOI:** 10.1371/journal.pone.0293433

**Published:** 2024-02-09

**Authors:** Nima Hajizadeh, Amirhossein Heidari, Sara Sadeghi, Azadeh Goodarzi

**Affiliations:** 1 School of Medicine, Iran University of Medical Sciences, Tehran, Iran; 2 Rasool Akram Medical Complex Clinical Research Development Center (RCRDC), Iran University of Medical Sciences, Tehran, Iran; 3 Faculty of Medicine, Tehran Medical Sciences, Islamic Azad University, Tehran, Iran; 4 Department of Medicine, New York Health System, South Brooklyn Hospital, New York, NY, United States of America; 5 Department of Dermatology, Faculty of Dermatology, Rasool Akram Medical Complex Clinical Research Development Center (RCRDC), School of Medicine, Iran University of Medical Sciences, Tehran, Iran; Chung Shan Medical University, TAIWAN

## Abstract

**Background:**

Cicatricial alopecia (CA) refers to various conditions that result in permanent hair loss. Treatment of CA has always been challenging. Regarding immune-mediated pathophysiology for many CA subtypes, the administration of Janus kinase (JAK) and tumor necrosis factor (TNF) inhibitors have potentiated the treatments of CA.

**Methods:**

After a thorough systematic search in PubMed/Medline, Embase, Web of Science, Scopus, Google Scholar, ClinicalTrials.gov, and WHO ICTRP, a total of 3,532 relevant records were retrieved and screened. Accordingly, 56 studies met the eligibility criteria and entered the review.

**Results:**

Among JAK inhibitors, oral tofacitinib was the most frequently reported and the most effective treatment in improving signs and symptoms of CA with minimal adverse effects (AEs). Baricitinib was another JAK inhibitor with sustained improvement while causing mild AEs. As a TNF inhibitor, adalimumab induced a rapid and stable improvement in signs and symptoms in most patients with rare, tolerable AEs. Thalidomide was the other frequently reported yet controversial TNF inhibitor, which caused a rapid and significant improvement in the condition. However, it may result in mild to severe AEs, particularly neuropathies. Infliximab is a TNF inhibitor with mostly favorable results, albeit in a few patients caused treatable dermatological AEs. Apremilast and certolizumab pegol caused an incomplete amelioration of signs and symptoms with no AEs. Lenalidomide is another TNF inhibitor that can induce temporary improvement in CA with probable AEs. It is noteworthy that utilizing adalimumab, infliximab, etanercept, golimumab, and an anonymous TNF inhibitor has induced paradoxical CA and other A.E.s in some patients.

**Conclusion:**

Recent studies have recommended JAK and TNF inhibitors, especially oral tofacitinib and adalimumab, as a new modality or adjuvant therapy to previous medications for primary CA. Nonetheless, monitoring AEs on a regular basis is suggested, and further extensive studies are required before definitive recommendations.

## 1. Introduction

Cicatricial alopecia (CA) is a heterogeneous group of cutaneous disorders characterized by fibrosis or hyalinized collagen replacing follicles, leading to irreversible destruction of hair follicles with subsequent scarring and baldness [1, 2]. The two types of CA are classified according to the target inflammation structure and the mechanisms responsible for the destruction of follicles, including primary and secondary forms [3]. Primary CA is caused by direct inflammatory damage to the hair follicle epithelium, which targets the hair follicle itself. Secondary CA results from inflammation or mechanical damage to surrounding tissues, affecting and destroying hair follicles. Physical damage, burns, radiation, infections, tumors, and chronic inflammatory diseases such as linear morphea can progress both types of alopecia. Based on the predominant cell type in the inflammatory infiltrate, primary CA is also categorized into five subtypes:: (I) Lymphocytic, including lichen planopilaris (LPP), frontal fibrosing alopecia (FFA), pseudopelade of brocq (PPB), and central centrifugal cicatricial alopecia (CCCA); (II) Neutrophilic, including folliculitis decalvans (FD) and dissecting cellulitis of the scalp (DCS), also known as perifolliculitis capitis abscedens et suffodiens (PCAS); (III) Mixed, including erosive pustular dermatosis of the scalp (EPDS) and folliculitis (acne) keloidalis; (IV) Nonspecific, defined as idiopathic CA with indefinite clinical and histopathological findings [1]. CA treatment aims to halt or slow the destruction of follicles, minimize the symptoms, and increase the chance of hair regrowth [3].

The tumor necrosis factor-alpha (TNF- α) is the first cytokine that appears in the blood, secreted primarily by activated macrophages and other immune cells, facilitating the initiation and regulation of inflammation in the body [4, 5]. TNF- α, as a mediator of autoimmune disease, significantly contributes to the pathogenesis of several chronic inflammatory and rheumatic diseases [6, 7]. TNF inhibitors, a class of drugs used to treat various inflammatory diseases, were investigated initially for treating rheumatoid arthritis [8]. The U.S. Food and Drug Administration (FDA) approved four types of TNF inhibitors for treating dermatological conditions, including plaque psoriasis [9].

The Janus kinase-signal transducer and activator of transcription (JAK-STAT) pathways play an integral role in intracellular cytokine signaling [10]. The Janus kinase (JAK) family comprises four members: JAK1, JAK2, JAK3, and Tyrosine kinase 2 (TYK2) [11]. JAK-STAT signaling pathways are of great significance in regulating the growth, development, and differentiation of immune and hematopoietic cells [12]. JAK inhibitors are a class of medications that work by inhibiting the phosphorylation and activation of different JAKs, thus blocking the cascades of cytokines-related inflammation [13, 14].

Despite improvements in the diagnosis and treatment of CA, the explanations for the exact pathogenesis of CA are still largely uncertain [15]. Prior investigations suggest multiple therapeutic options for CA, such as topical or systemic corticosteroids, antibiotics, mycophenolate mofetil, minoxidil, and systemic retinoids [1, 3, 16]. However, utilizing previous treatment approaches was not compelling enough to treat CA patients and may induce following drug-related complications and high incidence of adverse events (AEs) and relapses [3]. Based on intracellular inflammatory mechanisms associated with CA, new medications with specific anti-inflammatory pathways could be promising in managing CA with fewer AEs [17]. Recently, TNF and JAK inhibitors, previously proven to treat many dermatological and autoimmune diseases, were applied to CA patients to evaluate the promise and perils of these classes of drugs [9, 10, 14, 18]. In this study, we aim to systematically assess all aspects of TNF and JAK inhibitors in the management of CA.

## 2. Material and methods

The current systematic review was conducted according to the PRISMA checklists which are attached as supplementary documents (**S1** and **S2 Tables**).

### 2.1. Search strategy

A thorough systematic search was conducted in databases, including PubMed/Medline, Embase, Web of Science, and Scopus, as well as the Google Scholar search engine, ClinicalTrials.gov, and WHO ICTRP. A complete list of search terms is available in supplementary documents (**S3 Table**).

### 2.2. Eligibility criteria

Studies were eligible for inclusion in this systematic review if they were clinical trials, case reports, case series, or observational studies with an available English full text. The eligible source populations were individuals of any age treated with JAK inhibitors or TNF inhibitors for any type of CA. Reviews, experimental studies (*in vitro*/*ex vivo* or animal studies), and studies exclusively about non-cicatricial alopecia were excluded.

### 2.3. Data extraction and study selection

Two independent reviewers (NH, AH) conducted separate data extraction processes for all the eligible studies as follows: (I) study characteristics (author, year, design, sample size, treatment, outcome measurement, and follow-up duration), (II) patients’ characteristics (age, gender, cause of CA, baseline condition, disease duration, previous treatments), and (III) results (efficacy, outcome, recurrence, and A.E.s). Ultimately, the corresponding authors meticulously reviewed any disparities and disagreements in the extracted data and provided guidance for creating the final tables containing the extracted data.

### 2.4. Risk of bias assessment

Two investigators evaluated the methodological quality of the selected studies and the risk of bias independently (NH, AH). For these assessments, the National Institute of Health (NIH) Quality Assessment Tool for Observational Cohort and Cross-Sectional Studies [19], the NIH Tool for Before-After (Pre-Post) Studies with No Control Group [20], and Murad MH *et al*. for case series and case reports [21] were utilized, respectively (**S4 to S6 Tables**). In summary, the bias assessment of the included studies in this systematic review revealed varying levels of methodological quality. The evaluation of before-after studies with no control group (**S4 Table**) showed a range of quality scores from 4 (Poor) to 8 (Fair), highlighting issues such as unclear participant selection and inadequate sample sizes. Observational cohort and cross-sectional studies (**S5 Table**) received a single high-quality score of 9 (Fair), indicating robust methodology. In contrast, case reports and case series (**S6 Table**) had total quality scores varying from 2 to 6, with common issues including lack of patient representation, unclear exposure and outcome ascertainment, and incomplete reporting.

## 3. Results

### 3.1. Search results

**Fig 1:**
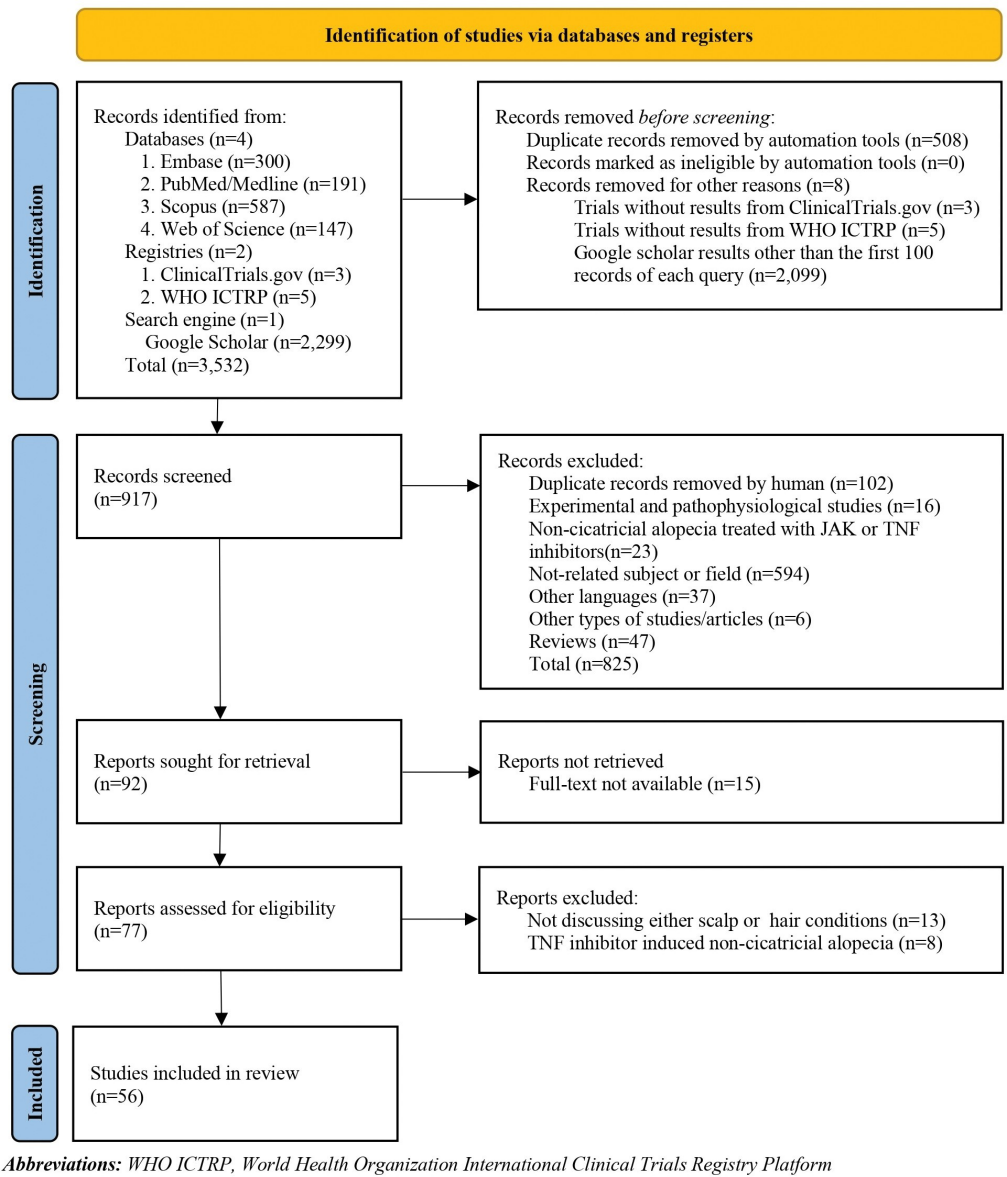
PRISMA 2020 flow diagram for new systematic reviews which included searches of databases and registers only.

A total of 3,532 records were found in a search up to December 24^th^, 2022. The number of 508 duplicates were detected and removed by the software. In the first and second phases of the screening, 917 studies were reviewed by reading the titles and abstracts to select the relevant publications by two independent reviewers (NH, AH). Disagreements were resolved with discussion or the consensus of the corresponding authors. Full texts were reviewed in the last screening phase, and 56 publications were included for data extraction. The inclusion process of our study-based PRISMA flowchart is illustrated in Fig 1.

### 3.2. Characteristics of eligible studies

The included studies encompass forty-five case reports, seven case series, three interventional studies, and one retrospective cohort. Among these, nine studies focused on JAK inhibitor therapies, while thirty-four studies investigated TNF inhibitor therapies for the treatment of CA. Moreover, fourteen studies reported CA as an AE of the treatment with TNF inhibitors. The sample size of the selected studies ranged from one to 118 patients, and a total of 342 patients were presented in the included articles. However, some of these patients were not compatible with the topic of the study and were not included in the results.

Causes of alopecia in the studies are as follows: LPP, FFA, FD, DCS (PCAS), PPB, EPDS, discoid lupus erythematosus (DLE), and subacute cutaneous lupus erythematosus (SCLE). Additionally, TNF-i that results in CA in some patients included adalimumab, infliximab, etanercept, golimumab, and one unidentified.

### 3.3. Janus kinase inhibitors

Nine studies reported various JAK inhibitors as treatments for CA in a total of 49 subjects. The results of these studies are completely demonstrated in **Table 1**.

**Table 1:** Characteristics of eligible studies utilizing Janus kinase inhibitors for the cicatricial alopecia treatment.

Study ID	Design of study	Sample Size	Gender Ratio (%F:M)	Age Mean (Range)	Condition[s]	Disease Duration (m)	Previous Treatments	Treatment[s] of Study	Outcome Measurement	Efficacy	Adverse Effects	Recurrence	Follow-up Duration (m)
Batra, 2020 [25]	C.R. ^(1)^	2	50:50	27–45	Pt ^(2)^ 1. LPP ^(3)^ Pt 2. FFA ^(4)^	Pt 1. 48 Pt 2. 45	All Pt. TAC ^(5)^, HCQ ^(6)^, Minoxidil Pt 1. MMF ^(7)^, Acitretin, Naltrexone, Clobetasol Pt 2. Avobenzone, Finasteride	Pt 1. Tofacitinib 5 mg ^(8)^ P.O. ^(9)^ BID ^(10)^ + Dapsone (after 2 m ^(11)^) Pt 2. Finasteride 5 mg PO QD ^(12)^ + HCQ 200 mg PO BID + TAC 2.5 mg/mL ^(13)^ inj ^(14)^ q ^(15)^1m + Topical Minoxidil 5% BID	Photo documentation Trichoscopy Clinical condition	Pt 1. Reduction in visibility of the scalp, Hair regrowth on the crown and vertex, Elimination of itching and hyperkeratosis Pt 2. Initial worsening of the condition for 11 m, Hair regrowth after 6 m, Elimination of inflammation and hyperkeratosis	N.R. ^(16)^	N.R.	Pt 1. 4 Pt 2. 17
Bordone, 2017 [26]	CR	4	NR	NR	Pt 1 to 3. LPP Pt 4. FFA	NR	NR	Tofacitinib 5 mg PO BID, TID ^(17)^ (after 1 m in Pt 1 and 2)	Clinical condition	Continuation of hair loss for 1 m in Pt 1 and 2 Later cessation of hair loss Improvement in pruritis	N.R.	Recurrence of disease within 1 m after discontinuation of the drug	Pt 1. 6 Pt 2 and 4. 12 Pt 3. 8
Eldik, 2019 [29]	CR	1	100:0	78	EPDS ^(18)^ + Concomitant RA ^(19)^	NR	Certolizumab pegol, TAC, Clobetasol, Tacrolimus, Mupirocin, Ketoconazole, Vinegar soak	Tofacitinib 5 mg PO BID	Clinical condition	Complete resolution	NR	NR	1
Jerjen, 2020 [30]	C.R.	3	67:33	31–42	F.D. ^(20)^	Pt 1. 144 Pt 2. 60 Pt 3. 96	All Pt. TAC, Cyclosporine, Minoxidil, Clobetasol propionate, Clindamycin Pt 1. HCQ, Isotretinoin, Minocycline, Rifampicin, Mupirocin Pt 2. MMF, Prednisone, Isotretinoin, Dapsone, Cephalexin Pt 3. Minocycline, Spironolactone	Pt 1. Tofacitinib 2.5 mg PO BID, 4 mg Q.D. (after 8 m) Pt 2 and 3. Tofacitinib 2.5 mg P.O. QD	Photo documentation Clinical condition Patient-reported outcomes Lab tests	Pt 1. Reduction in pustules and bleeding, Clinical and photographic improvement, cessation of pain, 80% improvement in itching Pt 2. Rapid resolution of inflammation, Inactivation of the disease Pt 3. Rapid reduction in hair shedding and pustules, Clinical and patient-reported improvement	Pt 1. LFT ^(21)^ derangement, Indigestion Pt 2. Mild eosinophilia, Transient total cholesterol elevation, Mild fatigue Pt 3. None	Recurrence of the disease 1, 6, and 12 m after discontinuation of the drug, respectively	Pt 1. 17 Pt 2 and 3. 22
Leung, 2019 [28]	CR	1	100:0	70	EPDS + Concomitant RA	21	Certolizumab pegol, TAC, Clobetasol, Tacrolimus, Mupirocin, Vinegar soak	Tofacitinib 5 mg PO BID	Photo documentation Clinical condition	Near-complete resolution of the ulceration, peripheral erosions, purulent discharge, and hemorrhagic crusts Re-epithelialization of the ulcer Improvement in R.A. symptoms	NR	NR	1
Moussa, 2022 [27]	CS ^(22)^	12	75:25	26–73	LPP (n = 7) FFA (n = 5)	24–312 Median (IQR ^(23)^) = 84 (39.6–144)	Tofacitinib (n = 4), HCQ, TCS ^(24)^, OCS ^(25)^, ILC ^(26)^, MMF, PRP ^(27)^, MTX ^(28)^, TCI ^(29)^, Minoxidil, Doxycycline, Ciclosporin, Bicalutamide, Tildrakizumab, Finasteride, Spironolactone	Baricitinib 3.4 mg (median dosage, titrated based on response and tolerability)	Clinical condition LPPAI ^(30)^ Lab tests	All Pt. Baseline LPPAI median (IQR) = 5.8 (4.7–6.9), Disease progression (n = 2) LPP Pt. Initial reduction in LPPAI (n = 5, reduction median = 46.5%), Maintained response in 43% FFA Pt. Initial reduction in LPPAI (n = 3, reduction median = 83.8%), Maintained response in 40%	Mild A.E.s ^(31)^ LFT derangement (n = 1) Elevated cholesterol (n = 1) Neutropenia (n = 1) Fatigue (n = 1)	NR	Max ^(32)^ of 10
Plante, 2020 [24]	CS	9	89:11	33–69	LPP and/or FFA	12–108	HCQ, TCS, TAC, MPA ^(33)^, Doxycycline, Dutasteride, Pioglitazone, Tacrolimus, Finasteride, Minoxidil, Pimecrolimus, Naltrexone, Prednisone, Ketoconazole, Leflunomide, Laser cap, Excimer laser	Pt 1 to 3. Tofacitinib cream 2% topical BID Pt 4 to 7. Tofacitinib 5 mg PO BID, TID (in Pt 6 and 7 after 10 and 7 m, respectively) Pt 8. Tofacitinib 11 mg P.O. Q.D. Pt 9. Tofacitinib cream 2% topical BID, Tofacitinib 5 mg PO BID (after 1 m)	Clinical condition Patient-reported outcomes Lab tests	Median time to initial response = 3 m Maintained improvement in all patients but Pt 2 and 5 Better response to P.O. treatment compared with topical	Mildly elevated T.G. ^(34)^ and cholesterol (n = 2) Pt 5. Mild transient Hb ^(35)^ and Cr ^(36)^ derangement Pt 9. Negative initial clinical response to topical therapy	NR	Max of 17
Sallee, 2018 [23]	CT ^(37)^	8	NR	NR	LPP	NR	NR	Tofacitinib 5 mg PO BID, TID (later in 2 patients)	Clinical condition LPPAI Lab tests	Significant improvement in LPPAI (reduction range 30–94%)	None	Recurrence of the disease after discontinuation of the drug Remission upon the treatment’s re-initiation	NR
Yang, 2018 [22]	CS	10	60:40	55 (33–68)	LPP	12–180	HCQ, TCS, TAC, MPA, Doxycycline, Pioglitazone, Finasteride, Minoxidil, Prednisone, Excimer laser	Tofacitinib 5 mg PO BID, TID (in Pt 1 and 7 after 2 and 4 m, respectively)	Photo documentation Clinical condition LPPAI Lab tests	Significant improvement in LPPAI (reduction range 30–94%) Clinical response (n = 8) Improvement in erythema, scaling, and hair density	No significant change in lab tests Pt 7. 4.5 Kg ^(38)^ weight gain	Recurrence of the disease after discontinuation of the drug (n = 1)	Max of 19

**Abbreviations:** (1) C.R., case report; (2) Pt, patient[s]; (3) LPP, lichen planopilaris; (4) FFA, frontal fibrosing alopecia; (5) TAC, Triamcinolone acetonide; (6) HCQ, Hydroxychloroquine; (7) MMF, Mycophenolate mofetil; (8) mg, milligram[s]; (9) P.O., orally; (10) BID, twice a day; (11) m, month[s]; (12) Q.D., daily; (13) mL, milliliter[s]; (14) inj, injection[s]; (15) q, every; (16) N.R., not reported; (17) TID, thrice a day; (18) EPDS, erosive pustular dermatosis of the scalp; (19) R.A., rheumatoid arthritis; (20) F.D., folliculitis decalvans; (21) LFT, liver function test; (22) C.S., case series; (23) IQR, interquartile range; (24) TCS, topical corticosteroid; (25) OCS, oral corticosteroid; (26) ILC, intralesional corticosteroid; (27) PRP, platelet-rich plasma; (28) MTX, Methotrexate; (29) TCI, topical calcineurin inhibitor; (30) LPPAI, lichen planopilaris activity index; (31) A.E., adverse effect; (32) max, maximum; (33) MPA, Mycophenolic acid; (34) T.G., triglyceride; (35) Hb, hemoglobin; (36) Cr, creatinine; (37) C.T., clinical trial; (38) Kg, kilogram[s].

#### 3.3.1. Lichen planopilaris (LPP) and frontal fibrosing alopecia (FFA)

Six studies investigated a total of 49 peers with LPP or FFA treated with JAK inhibitors. Oral tofacitinib therapy was the most frequent treatment and resulted in a mostly sustained and significant improvement in lichen planopilaris activity index (LPPAI) [22, 23], signs, and symptoms [22, 24–26]. A challenge/rechallenge phenomenon was reported in some studies, indicating the dependence of clinical improvement on the continuation of the medication [22, 23, 26]. Furthermore, cessation of hair loss [26] and even hair regrowth [22, 25] was observed in a number of patients. Concurrently, it’s important to highlight that while topical tofacitinib showed effectiveness in a majority of cases, it fell short in terms of both effectiveness and sustainability when compared to its oral counterpart. [24].

Baricitinib was administered to treat 12 patients who failed previous treatments, including tofacitinib [27]. Most patients experienced an initial improvement in LPPAI; however, less than half maintained favorable results after six months. AEs were said to be rare, minimal, and tolerable in all subjects.

#### 3.3.2. Erosive pustular dermatosis of the scalp (EPDS)

Two individuals suffering from EPDS and concomitant rheumatoid arthritis (RA) were treated with oral tofacitinib [28, 29]. Notably, one has resisted the previous therapy with certolizumab pegol [29]. Both patients expounded an almost complete amelioration in signs and symptoms with no AEs during their follow-up period.

#### 3.3.3. Folliculitis decalvans (FD)

Three patients with relatively long-term FD showed a rapid and significant improvement while on tofacitinib therapy [30]. Minimal AEs were reported in two cases. Nevertheless, recurrence was spotted in all three after discontinuing the therapy.

### 3.4. Tumor necrosis factor inhibitors

In 34 publications assessing the effect of TNF inhibitors in managing CA, multiple TNF inhibitors were utilized in 141 patients. The individual results of the studies are consolidated in **Table 2**.

**Table 2:** Characteristics of eligible studies utilizing tumor necrosis factor-alpha inhibitors for the cicatricial alopecia treatment.

Study ID	Design of study	Sample Size	Gender Ratio (%F:M)	Age (y) Mean (Range)	Condition[s]	Disease Duration (m)	Previous Treatments	Treatment[s] of Study	Outcome Measurement	Efficacy	Adverse Effects	Recurrence	Follow-up Duration (m)
Alam, 2020 [31]	C.R. ^(1)^	1	100:0	61	LPP ^(2)^ + Concomitant HS ^(3)^ and RA ^(4)^	6	Certolizumab pegol, HCQ ^(5)^, MTX ^(6)^, Clobetasol, Clindamycin, Rifampin, Leflunomide, Sulfasalazine	Adalimumab 160 mg ^(7)^, 80 mg, 40 mg SC ^(8)^ q ^(9)^1w ^(10)^ (each for once)	Photodocumentation Clinical condition	Hair regrowth Reduction in redness of alopecic patches Improvement in both H.S. and R.A.	NR ^(11)^	NR	9
Alfadley, 2003 [44]	CR	2	0:100	25–31	DLE ^(12)^ + Concomitant SLE ^(13)^	Pt ^(14)^ 1. 45 Pt 2. 132	HCQ, Prednisone	Thalidomide 200 mg/d ^(15)^, 300 mg/d (after 4 w) P.O. ^(16)^, Tapered and discontinued after neuropathy onset	Photo documentation Clinical condition Lab tests	Pt 1. Improvement of edema and erythema, Normalization of ESR ^(17)^ and complement test results Pt 2. Clinical improvement after 2 m	Right sural nerve sensory neuropathy after 1 y ^(18)^ (Pt 1) and 9 m (Pt 2)	None	24
Alhameedy, 2019 [35]	C.R.	1	100:0	54	F.D. ^(19)^	108	TCS ^(20)^, ILC ^(21)^, Clindamycin, Rifampicin, Acitretin, Isotretinoin	Adalimumab 160 mg, 80 mg (after 2 w), 40 mg SC q1w (after 2 w)	Photodocumentation Clinical condition DLQI ^(22)^	Significant improvement in DLQI from 16 to 7, inflammation, signs, and symptoms after 3 m No new alopecic area	None	Recurrence of pustules and inflammation 2 w after discontinuation of the drug Remission within 1 m after re-initiation of the treatment	NR
Alsantali, 2021 [58]	C.R.	1	0:100	38	DCS ^(23)^ (PCAS ^(24)^)	77	Isotretinoin, Clindamycin, Doxycycline, Co-amoxiclav	Adalimumab 80 mg, 40 mg SC q1w (after 1 w)	Photo documentation Clinical condition	Excellent clinical response after 1 m Decrease in pain and swelling Cessation of discharge Hair regrowth after 2 m	NR	NR	2
Brandt, 2008 [45]	C.R.	1	0:100	24	DCS (PCAS)	48	Isotretinoin, Doxycycline, Ciprofloxacin, Dapsone	Infliximab 5 mg/Kg ^(25)^ inf ^(26)^ q8w (for 12 m)	Photo documentation Clinical condition	Excellent clinical response Hair regrowth after 4 m Maintained response 12 m after the treatment discontinuation	None	None	24
Cautela, 2020 [46]	C.S. ^(27)^	7	NR	NR	DCS (PCAS)	NR	NR	Adalimumab 160 mg, 80 mg (after 2 w), 40 mg SC q1w (after 2 w)	Clinical condition	Rapid improvement of clinical condition and inflammation Partial hair regrowth	None	None	NR
Fässler, 2020 [39]	C.R.	1	0:100	28	F.D.	60	TCS, Isotretinoin, Clindamycin, Rifampin, Dapsone, PDT ^(28)^	Apremilast PO	Photo documentation Clinical condition Trichoscopy Patient-reported outcomes	Rapid improvement of clinical condition and inflammation Almost complete resolution of erythema, follicular pustules, oozing, and crusts Elimination of follicular hyperkeratosis and erythema, pain, and itching No significant change in tufted hairs, hair diameter diversity, cicatricial white patches, and yellow dots	N.R.	Rapid recurrence of the disease after discontinuation of the drug at 7^th^ w Remission of the disease after re-initiation of the treatment	6.25
Garbelini-Lima, 2021 [62]	CR	1	100:0	44	DLE + Concomitant SLE	1	HCQ, Prednisone	Thalidomide 100 mg PO QD ^(29)^ Clobetasol propionate cream 0.05% topical BID ^(30)^ Prednisone 60 mg/d P.O. (for 2 m)	Photo documentation Clinical condition	Total hair regrowth after 9 m	N.R.	None	18
George, 2001 [34]	C.R.	1	100:0	41	LPP	NR	HCQ, Prednisone, TAC ^(31)^, Diflorasone diacetate	Thalidomide 150 mg, 50 mg (after 1 m) PO QD	Photo documentation Clinical condition	Significant hair regrowth after 1 m	N.R.	None	18
Hession, 2010 [43]	C.R.	2	50:50	39–61	Pt 1. DCS (PCAS) Pt 2. Disseminated GA ^(32)^	NR	NR	Adalimumab 40 mg SC q2w	Clinical condition	Pt 1. Complete remission after 5 m Pt 2. Remaining of only faint macular erythema after 4 w, Continued remission for 6 m	N.R.	Pt 1. Recurrence of the disease 6 m after discontinuation of the drug Pt 2. Recurrence of erythematous violaceous plaques 4 m after discontinuation of the drug All Pt. Remission of disease after re-initiation of the treatment	Pt 1. 19 Pt 2. 13
Hoy, 2022 [42]	C.R.	1	0:100	42	FD	N.R.	Clindamycin, Rifampin, Minocycline, Isotretinoin, Dapsone	Certolizumab pegol 400 mg, 200 mg SC q2w (after 6 w)	Photo documentation Clinical condition PGA ^(33)^ Patient-reported outcomes	Rapid and maintained cessation of pain and itching after 1 m Improvement in inflammation, erythema, and pustules Improvement in PGA from "severe" to "mild to moderate" Not complete resolution of inflammation after 2 y of treatment	None	Minor fluctuations in disease activity	33
Iorizzo, 2022 [36]	C.S.	23	26:74	21–67	F.D.	12–120	Apremilast (n = 1), TAC, OCS ^(34)^, Doxycycline, Clindamycin, Rifampin, Minocycline, Co-trimoxazole, Dapsone, Isotretinoin, Fusidic acid, Clobetasol	Adalimumab 160 mg, 80 mg SC q2w (after 2 w)	Photo documentation Clinical condition	Rapid and maintained clinical improvement (n = 21) Improvement of inflammation Stabilization of the size of the patch No new lesions	Mild GI ^(35)^ symptoms (n = 2)	NR	Max ^(36)^ of 24
Jouanique, 2004 [33]	CT ^(37)^	6	NR	NR	LPP (n = 4) PPB ^(38)^ (n = 2)	NR	NR	Thalidomide 100 mg/d, increased up to 200 mg/d (after 1 m based on response) PO	Photo documentation Clinical condition Hair counts by macro photographs	All Pt. Average 16% hair loss remaining (n = 4) 2 LPP Pt. Worsening of the condition, 5 and 40% decrease in hair counts 2 LPP Pt. No hair regrowth, Loss to F/U ^(39)^ after 2 and 4 m, 11 and 12% decrease in hair counts PPB Pt. No clinical change	Slowly progressive sensory neuropathy 12 m after drug discontinuation (n = 1)	N.R.	6
Knop, 1983 [61]	CT	60	75:25	NR	DLE	6–384	TCS, OCS, Antimalarials, Immunosuppressants, Light protection	Thalidomide 200 mg PO BID, Monthly decrease until 50–100 mg/d (based on response)	Photo documentation Clinical condition Lab tests	Significant improvement (n = 54) Complete Regression (n = 39) Relatively better response in female patients	Paresthesia and/or sensory loss w/o peripheral neuropathy (n = 3) Slight mainly sensory peripheral neuropathy (n = 8) Moderate sensory peripheral neuropathy and slight motor lesions (n = 3) Severe peripheral neuropathy (n = 1) Somnolence (n = 60) Constipation (n = 19) Circulatory disturbances (n = 7) Oral dryness (n = 2) Rash (n = 7) Edema (n = 4) Disappearance of the A.E.s ^(40)^, except some sensory disturbances after discontinuation of the drug	Recurrence of the disease after discontinuation of therapy (n = 30) or decrease in dosage (n = 7) Remission of the disease after re-initiation of the treatment or increase in dosage	24
Kreutzer, 2014 [32]	C.R.	3	100:0	50–68	Pt 1 and 2. F.D. Pt 3. LPP	Pt 1. 129 Pt 2. 112 Pt 3. 24	Pt 1 and 2. MTX, OCS, Clindamycin, Rifampin, Dapsone, Tetracycline, Isotretinoin Pt 3. HCQ, TCS	Adalimumab 40 mg SC q2w	Photo documentation Clinical condition	Pt 1 and 2. Significant remission within 2 and 3 m, respectively Pt 3. Marked clinical improvement, Regression of the peripilar scaling, and erythema	N.R.	Recurrence of disease 3 m after discontinuation of the drug	Max of 6
Kurokawa, 2021 [59]	C.R.	1	0:100	18	DCS (PCAS) + Concomitant HS and NCA ^(41)^	73	Faropenem, Saireito herb (TJ-114)	Adalimumab 160 mg, 80 mg SC q2w (after 2 w)	Photo documentation Clinical condition IHS4 ^(42)^	Significant clinical improvement within 1 m Scar formation and re-epithelialization of the hemorrhagic ulcer Cessation of severe pain, itching, and insomnia Improvement of the occipital nodules Hair regrowth Significant improvement of the face NCA and the buttocks’ nodules IHS4 reduction from 3 to 0	NR	NR	NR
Malara, 2022 [47]	C.R.	4	50:50	45–59	DLE (Scalp lesions only present in Pt 1)	Pt 1. 180 Pt 2. 108 Pt 3. N.R. Pt 4. 144	Pt 1. HCQ, CQ ^(43)^, TCS, Azathioprine Pt 2. HCQ, OCS, Dapsone Pt 3. HCQ, OCS, Azathioprine Pt 4. OCS, Azathioprine, Cyclosporine, Cyclophosphamide	Thalidomide 50 mg PO QD	Photo documentation Clinical condition	Rapid clinical improvement within 1–2 m Decrease in erythema size and lesions’ thickness Complete resolution after 3–13 w	None	None	9
Mansouri, 2016 [48]	C.R.	2	0:100	27–48	Pt 1. DCS (PCAS) + Concomitant H.S. and abnormal LFT ^(44)^ Pt 2. DCS (PCAS)	Pt 1. 240 Pt 2. 48	All Pt. OCS, Isotretinoin, Multiple antibiotics Pt 1. Zinc sulfate, Surgical excision and drainage Pt 2. TCS	Pt 1. Adalimumab 80 mg, 40 mg SC q2w (after 1 w) Pt 2. Infliximab 5 mg/Kg inj ^(45)^ (baseline, after 2 and 6 w, then q8w)	Photo documentation Clinical condition DLQI ^(46)^ Lab tests	All Pt. No prevention from CA ^(47)^ progression Pt 1. Improvement in inflammation, discharge, pain, and LFT after 1 m, DLQI reduction from 21 to 10 after 5 m Pt 2. Reduction in inflammation, symptoms, and odor within 3 m, DLQI reduction from 18 to 6 after 12 m	NR	None	Max of 20
Martin-García, 2015 [49]	CR	1	0:100	30	DCS (PCAS)	183	TAC, Doxycycline, Ciprofloxacin, Isotretinoin	Adalimumab 80 mg, 40 mg SC q2w (after 1 w)	Photo documentation Clinical condition Patient-reported outcomes	Progressive decrease in pain and swelling after 1 m Complete resolution of inflammatory lesions after 7 m	None	None	24
Masnec, 2018 [50]	C.R.	1	0:100	26	DCS (PCAS) + Concomitant HS and facial acne	N.R.	Multiple antibiotics, Isotretinoin	Adalimumab 80 mg (baseline, after 1 d and 2 w), 40 mg SC q1w (after 2 w)	Photo documentation Clinical condition DLQI	Excellent clinical response after 10 w Significant improvement in all symptoms, secretion, pain, and inflammation No new nodules or sinus tracts Resolution of the facial acne DLQI reduction from 27 to 1	None	None	15
Maxon, 2020 [60]	C.R.	1	0:100	37	DCS (PCAS)	156	ILC, Isotretinoin, antibiotics, surgical excision	Adalimumab 40 mg SC q1w	Photo documentation Clinical condition	Significant clinical improvement after 2 m Significant hair regrowth and reduction in bogginess and tenderness of the scalp after 6 m Further improvement after 2 y by switching to Acitretin	N.R.	None	24
Mihaljević, 2012 [40]	C.R.	1	0:100	45	FD	N.R.	Methylprednisolone, Multiple antibiotics, Isotretinoin, Zinc, Topical treatments	Infliximab 5 mg/Kg inj (baseline, after 2 and 6 w, then q8w)	Photo documentation Clinical condition Lab tests	Rapid remission of inflammatory lesions after 3 sessions No lab test abnormalities	None	None	12
Minakawa, 2021 [51]	C.R.	1	0:100	30	DCS (PCAS) + Concomitant HS	144	Clindamycin, Minocycline, Nadifloxacin, Benzoyl peroxide	Adalimumab SC	Photo documentation Clinical condition DLQI Lab tests	Cessation of purulent secretions and pain after 1 m Normalization of WBC ^(48)^ count, CRP ^(49)^, and KL-6 ^(50)^ antigen levels DLQI reduction from 2 to 0	None	None	18
Navarini, 2010 [52]	CR	3	0:100	27–30	Pt 1 and 2. DCS (PCAS) Pt 3. DCS (PCAS) + Concomitant HS	NR	NR	Adalimumab 80 mg, 40 mg SC q2w (after 1 w)	Photodocumentation Clinical condition Patient-reported outcomes Biopsy findings	All Pt. Rapid clinical improvement within 8 w, Significant reduction in disease activity and symptoms after 3 m, No change in residual pathological structures Pt 1 and 3. Reduction in inflammatory infiltrate, Increase in fibrosis and cicatrization	N.R.	Pt 3. Re-activation of the disease after discontinuation of the drug for 4 w, remission of the disease after re-initiation	NR
Sanchez-Diaz, 2021 [53]	CS	8	12:88	14–55	Pt 1 and 2. Facial HS Pt 3 and 4. DCS (PCAS) Pt 5. Nape H.S. Pt 6 to 8. H.S. lymphedema	15–204	Pt 1 and 2. Doxycycline, Clindamycin, Dapsone Pt 3, 4, and 6 to 8. Doxycycline, Clindamycin, Rifampin, Isotretinoin, Acitretin, PDT Pt 5. Doxycycline, Isotretinoin, Colchicine	Pt 1. Adalimumab 40 mg SC q1w Pt 2, 4, 7 and 8. Adalimumab 80 mg SC q2w Pt 3. Adalimumab SC, Infliximab 5 mg/Kg inf q8w (after 60 w) Pt 5. Adalimumab 80 mg SC q2w, q1w (after 80 w) Pt 6. Ustekinumab 90 mg inj q6w	Photo documentation Clinical condition IHS4 Patient-reported outcomes	Pt 1, 2, and 4. Rapid and maintained improvement Pt 3. Secondary failure of Adalimumab therapy, Disease control after 16 w of Infliximab therapy Pt 5. Favorable response to therapy until 12 w, Later secondary failure, and progressive worsening, Partial improvement after an increase in dosage Pt 6. Decrease in disease activity Pt 7 and 8. Poor response to the treatment despite dosage intensification and adjuvant therapies	None	N.R.	24–128
Sand, 2015 [38]	R.C. ^(51)^	118	NR	NR	DCS (PCAS) (n = 2 males) FD (n = 1 male) Other conditions (n = 115)	NR	NR	DCS and F.D. Pt. Adalimumab 40 mg SC q2w Other Pt. Various treatments	Clinical condition Lab tests	An elderly DCS Pt. Complete clearance of the lesions within 3 m A young DCS Pt. No clinical response after 6 m A young F.D. Pt. No clinical response after 3 m Other Pt. Various outcomes	DCS and F.D. Pt. None Other Pt. Various adverse events	NR	NR
Shah, 2009 [64]	CR	2	100:0	40–43	DLE + Concomitant SLE	Pt 1. 108 Pt 2. 120	All Pt. Thalidomide, MTX, HCQ, CQ, Quinacrine, Dapsone, Rituximab, Azathioprine Pt 1. MMF, TCS, IVIG ^(52)^ Pt 2. Prednisone, Cyclosporine	Lenalidomide 5 mg PO QD	Photo documentation Clinical condition CLASI ^(53)^ PtGA ^(54)^ Lab tests	Pt 1. Rapid clinical response within 1 m, Partial improvement in clinical condition, CLASI, and PtGA Pt 2. No clinical improvement after 6 m	Pt 1. Sustained neutropenia after 22 m, Intermittent hypokalemia Pt 2. Leukopenia, Mildly elevated LFT results, cellulitis of the legs, vasculitis	Pt 1. Slight increase in the disease activity after 10 m, Maintenance of the condition by increasing concomitant Prednisone from 5 to 10 mg/d until 22^nd^ m	Pt 1. 22 Pt 2. 6
Shireen, 2018 [37]	C.R.	1	0:100	23	F.D.	60	Doxycycline, Clindamycin, Terbinafine, Fluocinolone, Isotretinoin, Chymotrypsin, Paracetamol	Adalimumab biosimilar (Exemptia, ZRC-3197) 80 mg (baseline and after 2 w), 40 mg S.C. (after 1 and 2 w, then q2w for 12 w, then q4w)	Photo documentation Clinical condition	Complete remission of the lesions Hair regrowth	None	None	NR
Stevens, 1997 [54]	CS	16	94:6	25–65	Pt 1 to 4. DLE + Concomitant SLE Pt 5 to 11. DLE Pt 12. SLE Pt 13 to 15. SCLE ^(55)^ + Concomitant SLE Pt 16. SCLE	12–360	HCQ, Quinacrine, Prednisolone, Azathioprine, Cyclophosphamide	Thalidomide 50–100 mg/d P.O. (in short and rapidly tapering courses based on clinical response and A.E.s)	Photo documentation Clinical condition NCS ^(56)^	All Pt. Improvement of rash, erythema, photosensitivity, itching, local irritation, and clinical condition within 2 w, Maximum response after 16 w, decrease in hair loss w/o hair regrowth DLE + SLE Pt. Complete (n = 3) and partial (n = 1) response DLE Pt. Complete (n = 1), partial (n = 3), and no (n = 2) response, Drop-out (n = 1) SLE Pt. Complete response SCLE + SLE. Complete (n = 1) and partial (n = 2) response SCLE. Complete response	Discontinuation of the treatment in 1 DLE Pt after 2 w due to headache and dizziness Morning drowsiness needing dose reduction (n = 2) Mild peripheral sensory neuropathy (n = 1) Mild episodic peripheral paresthesia (n = 2) Resolution of neuropathy after discontinuation of the drug	Relapse of the disease (n = 6)	NR
Sukhatme, 2009 [55]	C.R.	1	0:100	39	DCS (PCAS)	72	TAC, Multiple antibiotics, Isotretinoin, Surgical excision	Adalimumab 80 mg, 40 mg SC q2w (after 1 w)	Photo documentation Clinical condition	Cessation of pain and purulent discharge after 1 m Improvement in bogginess, nodules, and erythema after 2 m Resolution of the lesions and normal hair growth after 5 m	N.R.	None	NR
Takahashi, 2019 [56]	C.R.	1	0:100	19	DCS (PCAS) + Concomitant HS	63	Clarithromycin, Zinc	Adalimumab 80 mg, 40 mg, 80 mg (after 3 m) SC q2w	Photo documentation Clinical condition Lab tests	Significant improvement in pain and cessation of purulent secretion after 1 m Partial hair regrowth and cessation of axillary inflammation after 3 m	Persistent increase in WBC count and CRP level within 3 m Normalization of lab tests after the increase in dosage	None	9
Tran, 2020 [63]	C.R.	1	0:100	13	DLE	3	HCQ, TCS, Prednisone, Sun protection	Thalidomide 100 mg, 50 mg (after 3 m) PO QD, Thrice per week (after 1 m)	Photo documentation Clinical condition Lab tests	No new lesions and significant terminal hair regrowth after 3 w Complete resolution of alopecia and improvement in lesions after 7 w	Mild drowsiness	None	4
Wollina, 2012 [57]	C.R.	1	0:100	30	DCS (PCAS) + Concomitant acne conglobata	12	Prednisolone, Rifampin, Isotretinoin, Ibuprofen, Metamizole, Amitriptyline, surgical excision	Infliximab 5 mg/Kg inf (baseline, after 2 and 6 w)	Photo documentation Clinical condition Lab tests	Rapid, significant improvement in inflammation, secretion, pain, and nodules after the 1^st^ session Normalization of CRP level Cessation of scarring process and lymph node swelling	Psoriasiform exanthema after the 2^nd^ session Complete resolution of the rash within several days using topical Prednicarbate	None	NR
Wu, 2013 [41]	CR	1	0:100	47	FD + Concomitant HS	120	MTX, MMF ^(57)^, TAC, Clobetasol, Prednisone, Minocycline, Clindamycin, Rifampin, Dapsone, Isotretinoin, Acitretin, Antiseptic shampoos	Infliximab 5 mg/Kg inf q2w (discontinued after 2 sessions)	Photo documentation Clinical condition	NR	Severe eruptive condyloma acuminata in perineal region after 2 w Rapid resolution of the warts using cryotherapy, Imiquimod cream 5%, and Podofilox solution 0.5%	NR	NR

**Abbreviations:** (1) CR, case report; (2) LPP, lichen planopilaris; (3) HS, hidradenitis suppurativa; (4) RA, rheumatoid arthritis; (5) HCQ, Hydroxychloroquine; (6) MTX, Methotrexate; (7) mg, milligram[s]; (8) SC, subcutaneous injection; (9) q, every; (10) w, week[s]; (11) NR, not reported; (12) DLE, discoid lupus erythematosus; (13) SLE, systemic lupus erythematosus; (14) Pt, patient[s]; (15) d, day[s]; (16) PO, orally; (17) ESR, erythrocyte sedimentation rate; (18) y, year[s]; (19) FD, folliculitis decalvans; (20) TCS, topical corticosteroid; (21) ILC, intralesional corticosteroid; (22) DLQI, dermatology life quality index; (23) DCS, dissecting cellulitis of the scalp; (24) PCAS, perifolliculitis capitis abscedens et suffodiens; (25) Kg, kilogram[s]; (26) inf, infusion[s]; (27) CS, case series; (28) PDT, photodynamic therapy; (29) QD, daily; (30) BID, twice a day; (31) TAC, Triamcinolone acetonide; (32) GA, granuloma annulare; (33) PGA, physician global assessment; (34) OCS, oral corticosteroid; (35) GI, gastrointestinal; (36) max, maximum; (37) CT, clinical trial; (38) PPB, pseudopelade of Brocq; (39) F/U, Follow-up; (40) AE, adverse effect; (41) NCS, nerve conduction study; (42) IHS4, international hidradenitis suppurativa severity score system; (43) CQ, Chloroquine; (44) LFT, liver function test; (45) inj, injection[s]; (46) DLQI, dermatology life quality index; (47) CA, cicatricial alopecia; (48) WBC, white blood cell; (49) CRP, C-reactive protein; (50) KL-6, Krebs von den Lungen 6; (51) RC, retrospective cohort; (52) IVIG, intra-venous immunoglobulin; (53) CLASI, cutaneous lupus erythematosus disease area and severity index; (54) PtGA, patient general assessment; (55) SCLE, subacute cutaneous lupus erythematosus; (56) NCS, nerve conduction study; (57) MMF, Mycophenolate mofetil.

#### 3.4.1. Lichen planopilaris (LPP)

Treatment of LPP using adalimumab resulted in a remarkable improvement in signs and symptoms as well as hair regrowth in two patients [31, 32]; One was refractory to a previous TNF inhibitor (certolizumab pegol) and was simultaneously affected by hidradenitis suppurativa (HS) and RA [31]. The other patient experienced a recurrence of the disease three months after the discontinuation of adalimumab [32].

Thalidomide therapy for LPP led to controversial results in two studies. A group of patients experienced continuous or even deteriorating hair loss as well as a case of thalidomide-related slowly progressive sensory neuropathy [33]. In contrast, another study demonstrated thalidomide therapy resulted in rapid hair regrowth and stable outcomes in a subject with LPP [34].

#### 3.4.2. Folliculitis decalvans (FD)

Twenty-eight patients were treated with adalimumab for FD. This treatment resulted in a relatively rapid and mainly sustained improvement in the condition of most patients despite uncommon and tolerable AEs [32, 35–37]. However, a young patient did not respond to the treatment after three months [38]. It is also claimed that one patient with a satisfactory response to adalimumab had previously received apremilast, another TNF inhibitor, with no improvement [36]. The phenomenon of challenge and rechallenge [35] and flare-ups following medication discontinuation [36] were observed in a few of the cases. Despite a quick response and near-complete improvement in some signs and symptoms (with positive challenge/ rechallenge phenomenon) in one patient, apremilast did not lead to hair regrowth [39].

Other TNF inhibitors, such as infliximab, showed a rapid improvement in inflammatory lesions without AEs or recurrence in one FD patient [40]. In spite of the latter favorable result of infliximab in treating FD, a study reported the prompt occurrence of severe eruptive condyloma acuminata in the perineal region of one patient [41]. However, the lesions were rapidly resolved with proper treatments. Additionally, certolizumab pegol led to a noteworthy improvement in a patient with FD, although the improvement was not complete [42].

#### 3.4.3. Dissecting cellulitis of the scalp (DCS) or perifolliculitis capitis abscedens et suffodiens (PCAS)

Adalimumab therapy has been depicted to be an effective treatment for DCS (PCAS) with appealing clinical outcomes [43]. A prompt and substantial improvement in clinical condition has emerged in several patients; some were also affected by other concurrent dermatological disorders [44–57]. Additionally, hair regrowth was successfully detected in a group of patients [46, 55, 56, 58–60]. Conversely, some patients exhibited no alteration in the progression of alopecia [48], did not manifest a favorable clinical response [38], experienced secondary treatment failure [53], and, in a few cases, even demonstrated an exacerbation of fibrosis and cicatrization, along with no discernible change in residual pathological structures [52]. The only reported AE was a reversible dose-dependent change in laboratory test results in one patient [56].

In the patient with secondary failure of adalimumab therapy, switching to infliximab stabled the disease after 16 weeks [53]. Two other patients receiving infliximab therapy for DCS (PCAS) had a similar clinical response. According to the details, one of the DCS (PCAS) cases experienced an abrupt, notable amelioration in signs and symptoms after the first therapy session, followed by a delayed halting in the scarring process. Nonetheless, the patient experienced an AE in the form of reversible psoriasiform exanthema [57]. Infliximab in other patients maintained a satisfactory clinical response as well as hair regrowth with no AE [45].

#### 3.4.4. Discoid lupus erythematosus (DLE)

Treating DLE with thalidomide was reported in six studies. All studies showed an instant and significant improvement in signs and symptoms in patients with DLE [44, 47, 54, 61–63]. Notably, substantial hair regrowth was reported in two cases [62, 63]. Meanwhile, several mild to severe AEs were observed, including neuropathies, headache, dizziness, drowsiness, constipation, rash, and edema [44, 54, 61, 63]. A dose-dependent clinical response and phenomenon of challenge and rechallenge regarding the efficacy of thalidomide were reported in one of these studies [61].

Furthermore, two patients with a history of unsuccessful thalidomide therapy were switched to lenalidomide. Both patients were also affected by systemic lupus erythematosus (SLE). One of them experienced a rapid clinical response and improvement in signs and symptoms, while the other one failed to reach any clinical improvement. Moreover, disturbances in laboratory test results in both patients and vasculitis, in addition to cellulitis of the legs in the non-responding patient, were reported as AEs [64].

#### 3.4.5. Subacute cutaneous lupus erythematosus (SCLE) and pseudopelade of brocq (PPB)

In a patient with SCLE and concomitant SLE, thalidomide therapy resulted in a complete clinical response with no severe AEs and amelioration in signs and symptoms as well as cessation of hair loss, and no hair regrowth was observed [54]. Failure in clinical improvement was also documented in two patients with PPB.

### 3.5. Tumour Necrosis Factor (TNF) inhibitor therapy-induced cicatricial alopecia

Thirteen studies reported the induction of CA following the prescription of TNF inhibitors for different clinical conditions in a total of 14 individuals. The results of these studies are presented in **Table 3.**

**Table 3:** Characteristics of eligible studies with tumor necrosis factor inhibitors treatment induced cicatricial alopecia.

Study ID	Design of study	Sample Size	Gender Ratio (%F: M)	Age (y) Mean (Range)	Baseline Condition[s], Duration (m)	Previous Treatment[s]	Treatment[s] of Interest	Concomitant Treatment[s] and Habit[s]	Outcome Measurement	Treatment Outcome[s]	Succeeding Actions and Therapies	Subsequent Outcome[s]	Follow-up Duration (m)
Abbasi, 2009 [73]	C.R. ^(1)^	1	0:100	8	Psoriasis, 24	MTX ^(2)^, Biologic agents, NB-UVB ^(3)^ phototherapy	Etanercept 0.8 mg ^(4)^/Kg ^(5)^ SC ^(6)^ q ^(7)^1w ^(8)^ (>24 m ^(9)^)	N.R. ^(10)^	Clinical condition	Improvement of psoriasis 9x8 cm ^(11)^ LPP ^(12)^ alopecic patch	Terbinafine 125 mg PO ^(13)^ QD ^(14)^ (for 4 w) Class I TCS ^(15)^ Topical Tacrolimus	No clinical improvement	24
Amschler, 2018 [68]	C.R.	1	100:0	36	CD ^(16)^, N.R.	Azathioprine	Infliximab 5 mg/Kg inf ^(17)^ q8w (6 sessions) Adalimumab SC	Azathioprine Smoking	Photo documentation Clinical condition Histological examination	Acceptable control of CD TNF-i ^(18)^-induced paradoxical palmoplantar pustulosis after 3 sessions Same A.E. ^(19)^ and erythematous scaly plaques on the scalp after another 3 sessions	Step 1. TCS, Vitamin D3 analogs Step 2. Substitution of Infliximab with Adalimumab Step 3. Potent TCS, Octenidine dihydrochloride, Discontinuation of Adalimumab Step 4. Ustekinumab 90 mg (baseline, after 4 w, then q12w)	Step 1. Successful treatment of palmoplantar lesions Step 2. Control of CD, Resolution of the palmoplantar lesions within 6 m, Worsening of the scalp involvement, Recurrent extensive erosions and exudation with CA ^(20)^, Psoriasiform dermatitis with parakeratosis and F.D. ^(21)^ Step 3. Partial response, No improvement in inflammation of the scalp after 7 m Step 4. Complete resolution of the scalp disease after 4 w w/o ^(22)^ recurrence, Residual scars	36
Brehon, 2020 [65]	C.R.	3	100:0	30–53	Pt ^(23)^ 1 and 2. R.A. ^(24)^, N.R. Pt 3. AS ^(25)^ + CD, NR	All Pt. MTX Pt 2. Leflunomide Pt 3. Infliximab, Azathioprine	Pt 1 and 3. Adalimumab Pt 2. Certolizumab pegol	Pt 1. Rituximab Pt 2. Leflunomide, Corticosteroid Pt 3. None	Photo documentation Clinical condition Histological examination Lab tests	All Pt. Complete control of underlying disease Pt 1. CA, Widespread DLE ^(26)^ after 24 m Pt 2. Localized DLE of the arms and ears after 2 m Pt 3. CA, Widespread DLE after 48 m, Inflammatory joint pain, Synovitis of the hands, PEff ^(27)^, Lymphopenia, Decreased complement level	All Pt. Discontinuation of TNF-i therapy, Short-term HCQ ^(28)^ Pt 2. TCS Pt 3. Prednisone 1 mg/Kg P.O. Q.D., Rituximab, Belimumab, Low-dose IL-2 ^(29)^	Pt 1 and 2. Disappearance of the skin lesions within 3 m Pt 3. Persistent articular and cutaneous activity with Rituximab therapy, Partial improvement with Belimumab therapy, Later relapse	Pt 1. 84 Pt 2 and 3. 18
El Shabrawi-Caelen, 2010 [66]	CR	2	100:0	19–31	Pt 1. CD, 36 Pt 2. CD, 24	Pt 1. Infliximab, Mesalazine, Azathioprine Pt 2. OCS ^(30)^, Sulfasalazine	Adalimumab 40 mg SC q2-3w	None	Photodocumentation Clinical condition Histological examination	Pt 1. Diffuse psoriasis and non-scarring alopecia after 3 m Pt 2. Moderate G.I. ^(31)^ symptoms, Severe scalp psoriasis, Progressive CA after 2 m	Pt 1. Discontinuation of TNF-i therapy Pt 2. N.R.	Pt 1. Resolution of the lesions, Complete hair regrowth Pt 2. NR	NR
Fernández-Torres, 2010 [70]	CR	1	0:100	37	Plaque psoriasis + Anemia + Recent respiratory infection + Abnormal LFT ^(32)^, >240	MTX, Cyclosporine, Acitretin, Psoralen-enhanced UVA ^(33)^ phototherapy	Infliximab 5 mg/Kg inf q8w	Cyclosporine 5 mg/Kg P.O. Q.D. (tapered and discontinued within 6 w) MTX 5 mg/w (after 7 m)	Photo documentation Clinical condition Histological examination PASI ^(34)^	Resolution of erythema and fever PASI reduction by 75% An outbreak of psoriasis in conjunction with a respiratory infection after 7 m LPP after 11 m from baseline worsening after each Infliximab session	Continuation of Infliximab therapy Deflazacort 35 mg P.O. Q.D. (tapered q3w to discontinuation)	Adequate control of psoriasis Mild response of LPP No new lesions	NR
Garcovich, 2008 [74]	C.R.	1	100:0	56	Psoriatic arthritis + Cutaneous psoriasis, 72	MTX, Cyclosporine	Etanercept 50 mg SC q1w	NR	Photo documentation Clinical condition Histological examination PASI ACR ^(35)^ score	Improvement of skin lesions and joint tenderness PASI and ACR scores reduced by 75 and 20%, respectively, after 12 w Advanced LPP after 32 w	Step 1. Discontinuation of Etanercept, Cyclosporine 3 mg/Kg P.O. Q.D., Etoricoxib 90 mg P.O. Q.D., Betamethasone 0.1% topical Q.D. Step 2. Re-initiation of Etanercept therapy Step 3. Discontinuation of Etanercept, MTX, NSAIDs ^(36)^	Step 1. Cessation of erythema and follicular hyperkeratosis after 3 m, Significant reduction in local pain and itching, Adequate control of psoriasis for 7 m before severe recurrence Step 2. Recurrence of LPP, Improvement of psoriatic arthritis after 12 w Step 3. Stability of involvement of the scalp	NR
Helm, 2018 [76]	C.R.	1	0:100	53	AS + A.D. ^(37)^ + Localized A.A. ^(38)^, N.R.	Adalimumab, MTX, Sulfasalazine	Golimumab	Minoxidil 2% topical Desonide topical Tacrolimus topical	Photo documentation Clinical condition	Flare of A.A. Two new 5x6 cm CA plaques Mild improvement in the skin changes An acute photo distributed lupus-like cutaneous reaction on the face	Continuing Golimumab therapy Sun protection Clobetasol solution topical QD or BID ^(39)^ TAC ^(40)^ 8 mg intralesional	Good control of AS Significant improvement of alopecic lesions	NR
Jayasekera, 2016 [69]	CR	1	100:0	12	Extended oligoarthritis + Bilateral optic nerve drusen + Asymptomatic arachnoid cyst, N.R.	MTX	Etanercept 25 mg SC q2w (for 57 m) Adalimumab 40 mg SC q2w (after 57 m, for 3 m)	None	Photo documentation Clinical condition Histological examination	Temporary good response to Etanercept therapy LPP after 3 m of Adalimumab therapy Discoid plaques on hips and thighs	Step 1. Clobetasol 0.05% topical Step 2. Sulfasalazine, Several intra-articular steroid inj ^(41)^ Step 3. Tocilizumab	Step 1. Resolution of most lesions, Remaining of some active lesions on the frontal scalp and left thigh after 6 m, recurrence of arthritis Step 2. Not controlled arthritis Step 3. Complete hair regrowth after 6 m	6
Lenzy, 2010 [77]	C.R.	1	100:0	35	IBD ^(42)^, NR	NR	TNF-i therapy	N.R.	Clinical condition Dermoscopy	2–6 cm psoriatic CA plaques Asymptomatic psoriatic plaques on the trunk, palms, and legs	Discontinuation of the TNF-i Super-potent TCS	NR	NR
McPhie, 2020 [71]	C.R.	1	100:0	31	IBD, NR	NR	Infliximab inf	NR	Photo documentation Clinical condition Dermoscopy Histological examination	Widespread pruritis after the 1^st^ session Severe pruritus and a predominantly abdominal rash after the 2^nd^ session	Step 1. Discontinuation of Infliximab therapy Step 2. Re-initiation of Infliximab therapy Step 3. Prednisone 35 mg P.O. Q.D., Betamethasone valerate 0.1% topical BID Step 4. Discontinuation of Infliximab therapy, Continuation of Prednisone and Betamethasone valerate therapies	Step 1. Complete resolution of the rash after 8 m, Recurrence of IBD 4 m later Step 2. Diffuse pruritic rash after 2 w, alopecia of 60% of scalp and eyebrows Step 3. No improvement, LPP, Lichenoid dermatitis, parakeratosis Step 4. Partial improvement	N.R.
Swale, 2003 [75]	C.R.	1	100:0	58	RA, 84 + DLE, 36	MTX, TCS, Betamethasone valerate, Cyclosporine, Indomethacin, Sulfasalazine, Folate	Etanercept 25 mg SC twice q1w, once q1w (after 8 w)	MTX 12.5 mg P.O. q1w Occasional TCS	Photo documentation Clinical condition Histological examination Lab tests	Significant improvement in R.A. Stabilization of DLE Extension and photosensitivity of the skin lesions	Step 1. Super-potent TCS, HCQ 200 mg P.O. Q.D. Step 2. Tacrolimus 0.1% topical, Mometasone furoate 0.1% topical Step 3. Discontinuation of Etanercept therapy	Step 1. Increase in R.A. activity and skin lesions, CA, SLE ^(43)^ Step 2. Temporary stabilization of the skin lesions, Later deterioration of SLE Step 3. Rapid worsening of R.A., Rapid resolution of all skin lesions, Minimal asymptomatic erythema and dryness after 4 w, 3 very small DLE areas on the neck after 7 m	42
Udkoff, 2016 [72]	C.R.	1	0:100	23	CD, N.R.	Azathioprine	Infliximab inf	Azathioprine	Photodocumentation Clinical condition Histological examination	CA in the form of F.D. after 8 m	Step 1. Discontinuation of infliximab therapy, Minocycline 100 mg PO BID Step 2. Betamethasone lotion topical BID, Mineral oil topical application with covering overnight, Coal tar 5% or Ketoconazole 2% or salicylic acid 6% shampoo at morning, Minocycline 100 mg PO BID Step 3. Tapering and discontinuation of topical therapies, ustekinumab	Step 1. Progression of CA, New pruritic scalp lesions within 1 m, Scalp psoriasis Step 2. Significant improvement in alopecia and scalp psoriasis, Complete hair regrowth, and no scalp scales after 4 m Step 3. No recurrence, Stabilization of CD w/o A.E.s	4
Walsh, 2013 [67]	C.R.	1	100:0	12	EOJIA ^(44)^, 72	N.R.	Adalimumab	N.R.	Clinical condition Histological examination	Rapidly progressive cutaneous eruption after 4 m LPP Discoid lesions on the hips and legs	Discontinuation of Adalimumab therapy Clobetasol propionate topical	Resolution of most lesions Residual active lesions on the frontal scalp and left thigh after 6 m Flare-up of EOJIA	NR

**Abbreviations:** (1) CR, case report; (2) MTX, Methotrexate; (3) NB-UVB, narrow-band ultraviolet B; (4) mg, milligram[s]; (5) Kg, kilogram[s]; (6) SC, subcutaneous injection; (7) q, every; (8) w, week[s]; (9) m, month[s]; (10) NR, not reported; (11) cm, centimeter[s]; (12) LPP, lichen planopilaris; (13) PO, orally; (14) QD, daily; (15) TCS, topical corticosteroid; (16) CD, Crohn’s disease; (17) inf, infusion[s]; (18) TNF-i, tumor necrosis factor inhibitor; (19) AE, adverse effect; (20) CA, cicatricial alopecia; (21) FD, folliculitis decalvans; (22) w/o, without; (23) Pt, patient[s]; (24) RA, rheumatoid arthritis; (25) AS, ankylosing spondylitis; (26) DLE, discoid lupus erythematosus; (27) PEff, pericardial effusion; (28) HCQ, Hydroxychloroquine; (29) IL-2, interleukin-2; (30) OCS, oral corticosteroid; (31) GI, gastrointestinal; (32) LFT, liver function test; (33) UVA, ultraviolet A; (34) PASI, psoriasis area and severity index; (35) ACR, American college of rheumatology; (36) NSAID, non-steroidal anti-inflammatory drug; (37) AD, atopic dermatitis; (38) AA, alopecia areata; (39) BID, twice a day; (40) TAC, Triamcinolone acetonide; (41) inj, injection[s]; (42) IBD, inflammatory bowel disease; (43) SLE, systemic lupus erythematosus; (44) EOJIA, extended oligojuvenile idiopathic arthritis.

#### 3.5.1. Adalimumab

Adalimumab therapy in some patients resulted in several AEs, including widespread DLE, inflammatory joint pain, abnormal laboratory tests [65], severe scalp psoriasis, moderate gastrointestinal (GI) symptoms [66], LPP, rapidly progressive skin lesions [67], and scarring alopecia [65–67]. Discontinuation of the treatment caused an improvement in skin manifestations of most patients [65, 67], while stopping the medication was not enough in one patient who was eventually commenced on belimumab therapy, leading to a delayed and partial improvement in adalimumab-induced skin lesions [65].

In another study, a patient with Crohn’s disease (CD) experienced adequate control of the condition after receiving infliximab [68]. Due to the incidence of palmoplantar pustulosis and erythematous scaly plaques on the scalp, infliximab was substituted with adalimumab, and subsequently, the palmoplantar lesions disappeared. However, several AEs, including worsening of the scalp, CA in the form of FD, and psoriasiform dermatitis with parakeratosis, occurred as well. Finally, after suspension of adalimumab and initiation of different therapies, treatment with ustekinumab was able to completely resolve the scalp disease with minimal residual scars.

Lastly, etanercept was administered for the treatment of extended oligoarthritis, bilateral optic nerve drusen, and an asymptomatic arachnoid cyst in a patient who showed good results [69]. The patient was then switched to adalimumab as maintenance therapy. Three months following the initiation of adalimumab treatment, the patient exhibited a recurrence of LPP, arthritis, and the appearance of discoid plaques on the hips and thighs. Various medications were administered, and complete hair regrowth was finally achieved by tocilizumab therapy.

#### 3.5.2. Infliximab

In two out of three patients receiving infliximab therapy, the occurrence of LPP and other AEs was reported [70, 71]. Deflazacort was added to the treatment regimen of one of the patients and caused a mild improvement in LPP without any new lesions [70]. Nevertheless, the other patient experienced a challenge/rechallenge phenomenon regarding both the efficacy and AEs of infliximab therapy. After trying different medications, cessation of infliximab following prolonged prednisone and betamethasone valerate treatments resulted in a partial improvement of the AEs [71].

The third patient was receiving infliximab for CD and experienced scalp psoriasis and CA in the form of FD [72]. After switching from infliximab to other treatments, a remarkable improvement in the scalp condition, complete hair regrowth, and stabilization of CD were obtained.

#### 3.5.3. Etanercept

Etanercept improved different manifestations of psoriasis in two patients while causing CA in the form of LPP [73, 74]. The phenomenon of challenge and rechallenge regarding both the therapeutic effect on psoriasis and the recurrence of alopecia was reported [74].

Moreover, the AEs of etanercept therapy were comparably more severe in a patient with RA [75] and DLE. Although the treatment temporarily stabilized the baseline conditions, it ultimately led to the development of CA and SLE, which were only responsive to the discontinuation of etanercept.

#### 3.5.4. Golimumab and others

Golimumab therapy in a patient with concomitant atopic dermatitis (AD), ankylosing spondylitis (AS), and localized alopecia areata (AA) was associated with an AA exacerbation, CA, and photodistributed lupus-like cutaneous reaction [76]. Adding sun protection, topical clobetasol solution, and intralesional triamcinolone acetonide (TAC) injections were beneficial for improving the alopecic lesions and stabilization of AS.

Lastly, an anonymous TNF inhibitor was used to treat inflammatory bowel disease (IBD) in a patient, resulting in psoriatic CA of the scalp and asymptomatic plaques on the body [77]. In order to manage the condition, discontinuation of the TNF inhibitor, as well as the application of super-potent topical steroids, was necessary.

## 4. Discussion

We systematically reviewed 56 studies regarding the efficacy and safety of JAK and TNF inhibitors in treating CA. A total of 342 patients with different causes of alopecia, including LPP, FFA, EPDS, FD., DCS (PCAS), DLE, PPB, SCLE, TNF inhibitor therapy-induced CA, and some non-cicatricial subtypes (were excluded from the current review) were reported in the studies. Among the included studies, nine and 34 articles assessed the therapeutic effects of JAK and TNF inhibitors in CA, respectively. The results of the current systematic review support that JAK and TNF inhibitors are potential therapeutic options for managing CA.

One proposed pathophysiology for CA argues the attack of the hair follicles by one’s immune system, resulting in inflammation and damage to the stem cells in the follicles and subsequent scarring and permanent hair loss [1, 2]. JAK inhibitors can suppress the activity of several cytokines and growth factors, such as interferon-gamma, interleukin (IL)-2, IL-6, and TNF, which may be involved in the pathogenesis of CA [13, 14]. As a result of inhibiting these cytokines, JAK inhibitors can prevent further damage to the hair follicles by reducing inflammation. Additionally, the inhibition of the JAK-STAT pathway may promote hair regrowth by activating and proliferating the stem cells embedded in the hair follicles, though the actual mechanism is unknown [78].

Tofacitinib (oral or topical) and baricitinib are both effective in improving CA; however, the effectiveness, sustainability of outcomes, and frequency of usage were relatively higher with oral tofacitinib. Hair regrowth was reported, and AEs were rare, mild, and tolerable in all patients. Tofacitinib targets the activity of JAK1 and JAK3, while baricitinib inhibits JAK1 and JAK2. These receptors render the signaling pathways of multiple cytokines and growth factors, contributing to inflammation in the follicles and immune-mediated damage [79].

In addition, JAK inhibitors have been effective in the induction of hair regrowth and improved the quality of life in patients with AA [80]. It seems that oral treatment has a remarkably higher response rate than the topical form of the medication. The response rate was no different between pediatric and adult patients. Consistent with these results, another study concluded that oral administration was highly favorable [81]. The topical formulations did not provide a satisfactory response for the patients. Notably, these agents are required to be administered chronically to maintain long-term response [82].

TNF is a proinflammatory cytokine that plays a crucial role in the pathogenesis of some autoimmune and inflammatory disorders [6, 82]. Upregulation of TNF in CA leads to the immune-mediated destruction of the hair follicles. TNF inhibitors, such as adalimumab, etanercept, and infliximab, can bind to and neutralize TNF, reduce inflammation, and prevent hair follicles from further damage [8, 9].

Adalimumab is an effective treatment for LPP, FD, and DCS (PCAS); most patients showed a rapid response and sustained clinical improvement, while hair regrowth was observed only in some. On the other hand, thalidomide therapy for LPP and DLE was of variable outcomes; some patients experienced continued or deteriorated hair loss, and others showed rapid hair regrowth and maintained results. Despite substantial improvement in signs and symptoms, thalidomide therapy may cause mild to severe AEs.

Infliximab therapy is an effective alternative to adalimumab for CA. Some patients experienced excellent clinical improvement and hair regrowth. Nonetheless, some reversible AEs, such as psoriasiform exanthema or severe eruptive condyloma acuminata in the perineal region, were observed. Certolizumab pegol therapy resulted in a remarkable amelioration of signs and symptoms of FD, though a complete improvement has not been achieved. The results suggested that TNF inhibitors, particularly adalimumab, can effectively treat CA. However, there is limited evidence to determine the efficacy and safety of such medications to treat each subtype of CA.

Recent literature expounded that people with a background of rheumatological or gastrointestinal disorders may experience induced new-onset psoriasis while receiving TNF inhibitors. Reversible alopecia was a random AE of treatment with TNF inhibitors [83, 84]. Compared to these studies, our systematic review focused on using TNF and JAK inhibitors in treating CA, a more severe, scarring, and difficult-to-treat type of alopecia. Based on existing evidence, both TNF and JAK inhibitors can effectively alleviate signs and symptoms of CA with minimal AEs. Nonetheless, the evidence of JAK inhibitors in treating CA was limited.

The literature suggests that TNF inhibitors can cause AEs, including cancer, serious infections, heart failure, and demyelinating disorders, such as multiple sclerosis and lupus-like syndrome, after administration in predisposed individuals [85]. Therefore, patients receiving these medications should be closely monitored and regularly followed up. Moreover, the potential of thalidomide in treating cutaneous conditions has recently re‐emerged precisely regarding its benefits in treating specific dermatological disorders unresponsive to traditional therapies [86]. However, due to its teratogenic effects, thalidomide should also be regulated. Hence, it can only be prescribed under strict conditions and obligatory contraception for women of childbearing age [87].

Recent investigations are constrained by several factors derived from smaller studies such as case reports and case series. Besides, observer bias is a common issue in current evidence, which occurs when studies are not blinded during treatment and outcome assessment. Selection and publication biases are also significant since only positive results will likely be published. A small sample size of the patients also limits statistical power. Despite all these issues, our results add to the growing literature on immunomodulatory therapies to treat CA. JAK inhibitors, especially oral tofacitinib, are promising options for treating LPP and FFA with minimal AEs. Although TNF inhibitors can effectively treat LPP and FD, more studies are mandatory to investigate their long-term efficacy and safety in different age groups.

## 5. Conclusion

In summary, the results of this systematic review convey valuable insights for clinicians in choosing appropriate medications for treating CA based on patient conditions. It is imperative to note that JAK and TNF inhibitors, particularly oral tofacitinib and injectable adalimumab, can be considered as new replacements or adjuvants to previous therapies for CA, particularly primary subtypes. Although these medications are efficacious, maintained, safe, and feasible treatments in most cases, patients should be regularly monitored for AEs. Other JAK and TNF inhibitors also demonstrated therapeutic potential for treating CA, but their controversial clinical effects and inconsistent AEs require close monitoring. Furthermore, some classes of JAK and TNF inhibitors have not been investigated in the management of CA to date. Lastly, more explanation is mandatory regarding the rare alopecia-inducing AE of TNF inhibitors in some conditions; it is crucial to halt TNF inhibitor therapy whenever such AEs appear before causing scar and irreversible baldness. Albeit confirmation of these findings requires large-scale randomized trial studies in the future prior to definitive recommendation.

## Supporting information

S1 Table PRISMA 2020 checklist for reporting systematic reviews. (DOCX)

S2 Table PRISMA 2020 checklist for abstracts of systematic reviews. (DOCX)

S3 Table The list of search strategies and final results on each database and register. (DOCX)

S4 Table The quality assessment included observational cohort and cross-sectional studies [19]. (DOCX)

S5 Table The quality assessment included before-after studies with no control group [20]. (DOCX)

S6 Table The quality assessment included case reports and case series by Murad et al. [21]. (DOCX)

## Abbreviations

AA: alopecia areata
ACR: American college of rheumatology
AD: atopic dermatitis
AE: adverse effect
AS: ankylosing spondylitis
BID: twice a day
CA: cicatricial alopecia
CCCA: central centrifugal cicatricial alopecia
CD: Crohn’s disease
CLASI: Cutaneous lupus erythematosus disease area and severity index
cm: centimeter[s]
CQ: Chloroquine
CR: case report
Cr: creatinine
CRP: C-reactive protein
CS: case series
CT: clinical trial
d: day[s]
DCS: dissecting cellulitis of the scalp
DLE: Discoid lupus erythematosus
DLQI: Dermatology life quality index
EOJIA: Extended oligojuvenile idiopathic arthritis
EPDS: Erosive pustular dermatosis of the scalp
ESR: Erythrocyte sedimentation rate
F/U: Follow-up
FD: Folliculitis decalvans
FFA: frontal fibrosing alopecia
GA: Granuloma annulare
GI: gastrointestinal
gr: gram[s]
Hb: Hemoglobin
HCQ: Hydroxychloroquine
HS: Hidradenitis suppurativa
IBD: Inflammatory bowel disease
IHS4: International hidradenitis suppurativa severity score system
IL: interleukin
ILC: Intralesional corticosteroid
IM: ntramuscular
inj: injection[s]
inf: infusion[s]
IQR: interquartile range
IVIG: Intra-venous immunoglobulin
JAK: Janus kinase
Kg: kilogram[s]
KL-6: Krebs von den Lungen 6
LFT: Liver function test
LPP: Lichen planopilaris
LPPAI: Lichen planopilaris activity index
m: month[s]
Max: maximum; mg: milligram[s]
mL: milliliter[s]
MMF: Mycophenolate mofetil
MPA: Mycophenolic acid
MTX: Methotrexat
NB-UVB: Narrow-band ultraviolet B
NCA: Nodulocystic acne
NCS: Nerve conduction study
NR: Not reported
NSAID: non-steroidal anti-inflammatory drug
OCS: Oral corticosteroid
PASI: Psoriasis area and severity index
PCAS: Perifolliculitis capitis abscedens et suffodiens
PDT: photodynamic therapy
PEff: pericardial effusion
PGA: Physician global assessment
PO: orally
PPB: Pseudopelade of Brocq
PRP: Platelet-rich plasma
Pt: patient[s]
PtGA: Patient general assessment
q: every
QD: Daily; RA: rheumatoid arthritis
RC: Retrospective cohort
SC: Subcutaneous injection
SCLE: Subacute cutaneous lupus erythematosus
SLE: systemic lupus erythematosus
TAC: Triamcinolone acetonide
TCI: Topical calcineurin inhibitor
TCS: Topical corticosteroid
TG: Triglyceride
TID: Thrice a day
TNF: tumor necrosis factor
UVA: Ultraviolet A
w: week[s]
w/o: without
WBC: white blood cell
y: year[s]
