# Supplementary material for: Tumor necrosis factor inhibitors and janus kinase inhibitors in the treatment of cicatricial alopecia: A systematic review
Source: PLoS One. 2024 Feb 9;19(2):e0293433. doi: 10.1371/journal.pone.0293433 (PMC10857607; doi:10.1371/journal.pone.0293433)
Supplement: S3 Table — (DOCX) [file pone.0293433.s003.docx]

**S3 Table**. The list of search strategies and final results on each database and register.

| Database or Search Engine | Search Terms | Number of Results |
| --- | --- | --- |
| ClinicalTrials.gov | Condition or disease:  Alopecia cicatrisata OR Cicatricial alopecia OR Scarring alopecia OR Lichen planopilaris OR Frontal fibrosing alopecia OR Coup de sabre OR Folliculitis decalvans OR Erosive pustular dermatosis OR Central centrifugal OR Dissecting cellulitis |  |
|  | Other terms (search No. 1):  Janus Kinase Inhibitor OR JAK inhibitor OR Jakinib OR Ruxolitinib OR Tofacitinib OR Tasocitinib OR Oclacitinib OR Baricitinib OR Peficitinib OR Upadacitinib OR Fedratinib OR Delgocitinib OR Filgotinib OR Abrocitinib OR Pacritinib OR Deucravacitinib  [Link](https://clinicaltrials.gov/ct2/results?cond=Alopecia+cicatrisata+OR+Cicatricial+alopecia+OR+Scarring+alopecia+OR+Lichen+planopilaris+OR+Frontal+fibrosing+alopecia+OR+Coup+de+sabre+OR+Folliculitis+decalvans+OR+Erosive+pustular+dermatosis+OR+Central+centrifugal+OR+Dissecting+cellulitis&term=Janus+Kinase+Inhibitor+OR+JAK+inhibitor+OR+Jakinib+OR+Ruxolitinib+OR+Tofacitinib+OR+Tasocitinib+OR+Oclacitinib+OR+Baricitinib+OR+Peficitinib+OR+Upadacitinib+OR+Fedratinib+OR+Delgocitinib+OR+Filgotinib+OR+Abrocitinib+OR+Pacritinib+OR+Deucravacitinib&type=&rslt=&age_v=&gndr=&intr=&titles=&outc=&spons=&lead=&id=&cntry=&state=&city=&dist=&locn=&rsub=&strd_s=&strd_e=&prcd_s=&prcd_e=&sfpd_s=&sfpd_e=&rfpd_s=&rfpd_e=&lupd_s=&lupd_e=&sort=) | 3 |
|  | Other terms (search No. 2):  Cerdulatinib OR Gandotinib OR Lestaurtinib OR Momelotinib OR Cucurbitacin OR CHZ868 OR Tumor Necrosis Factor OR TNF OR Cachectin OR Infliximab OR Adalimumab OR Etanercept OR Golimumab OR Certolizumab  [Link](https://clinicaltrials.gov/ct2/results?cond=Alopecia+cicatrisata+OR+Cicatricial+alopecia+OR+Scarring+alopecia+OR+Lichen+planopilaris+OR+Frontal+fibrosing+alopecia+OR+Coup+de+sabre+OR+Folliculitis+decalvans+OR+Erosive+pustular+dermatosis+OR+Central+centrifugal+OR+Dissecting+cellulitis&term=Cerdulatinib+OR+Gandotinib+OR+Lestaurtinib+OR+Momelotinib+OR+Cucurbitacin+OR+CHZ868+OR+Tumor+Necrosis+Factor+OR+TNF+OR+Cachectin+OR+Infliximab+OR+Adalimumab+OR+Etanercept+OR+Golimumab+OR+Certolizumab&type=&rslt=&age_v=&gndr=&intr=&titles=&outc=&spons=&lead=&id=&cntry=&state=&city=&dist=&locn=&rsub=&strd_s=&strd_e=&prcd_s=&prcd_e=&sfpd_s=&sfpd_e=&rfpd_s=&rfpd_e=&lupd_s=&lupd_e=&sort=) | No results |
|  | Other terms (search No. 3):  Thalidomide OR Lenalidomide OR Nafamostat OR Pentoxifylline OR ABP-501 OR Ajulemic OR IP-751 OR Golnerminogene OR PF-06410293 OR PF-06438179 OR Remtolumab OR ABT-122 OR AN-0128 OR Apeptico OR AP-301 OR Baicalein  [Link](https://clinicaltrials.gov/ct2/results?cond=Alopecia+cicatrisata+OR+Cicatricial+alopecia+OR+Scarring+alopecia+OR+Lichen+planopilaris+OR+Frontal+fibrosing+alopecia+OR+Coup+de+sabre+OR+Folliculitis+decalvans+OR+Erosive+pustular+dermatosis+OR+Central+centrifugal+OR+Dissecting+cellulitis&term=Thalidomide+OR+Lenalidomide+OR+Nafamostat+OR+Pentoxifylline+OR+ABP-501+OR+Ajulemic+OR+IP-751+OR+Golnerminogene+OR+PF-06410293+OR+PF-06438179+OR+Remtolumab+OR+ABT-122+OR+AN-0128+OR+Apeptico+OR+AP-301+OR+Baicalein&type=&rslt=&age_v=&gndr=&intr=&titles=&outc=&spons=&lead=&id=&cntry=&state=&city=&dist=&locn=&rsub=&strd_s=&strd_e=&prcd_s=&prcd_e=&sfpd_s=&sfpd_e=&rfpd_s=&rfpd_e=&lupd_s=&lupd_e=&sort=) | No results |
|  | Other terms (search No. 4):  Delenex OR DLX-105 OR Ortataxel OR Pegsunercept OR Allotrap OR Delmitide OR Kinoid OR Debio-0512 OR COVA322 OR GTPL10469 OR Placulumab OR ABBV-257 OR AST-005 OR AVX-470 OR Aurimune OR CYT-609 OR INB03 OR PF-05230905  [Link](https://clinicaltrials.gov/ct2/results?cond=Alopecia+cicatrisata+OR+Cicatricial+alopecia+OR+Scarring+alopecia+OR+Lichen+planopilaris+OR+Frontal+fibrosing+alopecia+OR+Coup+de+sabre+OR+Folliculitis+decalvans+OR+Erosive+pustular+dermatosis+OR+Central+centrifugal+OR+Dissecting+cellulitis&term=Delenex+OR+DLX-105+OR+Ortataxel+OR+Pegsunercept+OR+Allotrap+OR+Delmitide+OR+Kinoid+OR+Debio-0512+OR+COVA322+OR+GTPL10469+OR+Placulumab+OR+ABBV-257+OR+AST-005+OR+AVX-470+OR+Aurimune+OR+CYT-609+OR+INB03+OR+PF-05230905&type=&rslt=&age_v=&gndr=&intr=&titles=&outc=&spons=&lead=&id=&cntry=&state=&city=&dist=&locn=&rsub=&strd_s=&strd_e=&prcd_s=&prcd_e=&sfpd_s=&sfpd_e=&rfpd_s=&rfpd_e=&lupd_s=&lupd_e=&sort=) | No results |
|  | Other terms (search No. 5):  PMI-005 OR ABX-0401 OR ALX-0071 OR Celastrol OR CDP571 OR Afelimomab OR Camobucol OR CRX-191 OR AME-527 OR CYT-007-TNFQb OR ALS-00T2-0501 OR FR-133605 OR MDL-201112 OR MDL-201449 OR MDL-201449A  [Link](https://clinicaltrials.gov/ct2/results?cond=Alopecia+cicatrisata+OR+Cicatricial+alopecia+OR+Scarring+alopecia+OR+Lichen+planopilaris+OR+Frontal+fibrosing+alopecia+OR+Coup+de+sabre+OR+Folliculitis+decalvans+OR+Erosive+pustular+dermatosis+OR+Central+centrifugal+OR+Dissecting+cellulitis&term=PMI-005+OR+ABX-0401+OR+ALX-0071+OR+Celastrol+OR+CDP571+OR+Afelimomab+OR+Camobucol+OR+CRX-191+OR+AME-527+OR+CYT-007-TNFQb+OR+ALS-00T2-0501+OR+FR-133605+OR+MDL-201112+OR+MDL-201449+OR+MDL-201449A&type=&rslt=&age_v=&gndr=&intr=&titles=&outc=&spons=&lead=&id=&cntry=&state=&city=&dist=&locn=&rsub=&strd_s=&strd_e=&prcd_s=&prcd_e=&sfpd_s=&sfpd_e=&rfpd_s=&rfpd_e=&lupd_s=&lupd_e=&sort=) | No results |
| Embase | ('jak inhibit*':ti,ab,kw OR 'jakinib*':ti,ab,kw OR 'janus kinase inhibit*':ti,ab,kw OR 'tyrosine kinase inhibit*':ti,ab,kw OR 'jak-stat inhibit*':ti,ab,kw OR 'jaki*':ti,ab,kw OR 'jak i*':ti,ab,kw OR 'jak-i*':ti,ab,kw OR 'jak1 inhibit*':ti,ab,kw OR 'jak2 inhibit*':ti,ab,kw OR 'jak3 inhibit*':ti,ab,kw OR 'tyk2 inhibit*':ti,ab,kw OR 'janus kinase 1 inhibit*':ti,ab,kw OR 'janus kinase 2 inhibit*':ti,ab,kw OR 'janus kinase 3 inhibit*':ti,ab,kw OR 'tyrosine kinase 2 inhibit*':ti,ab,kw OR 'ruxolitinib':ti,ab,kw OR 'jakafi':ti,ab,kw OR 'jakavi':ti,ab,kw OR 'opzelura':ti,ab,kw OR 'incb018424':ti,ab,kw OR 'incb-018424':ti,ab,kw OR 'incb 018424':ti,ab,kw OR 'incb18424':ti,ab,kw OR 'incb-18424':ti,ab,kw OR 'incb 18424':ti,ab,kw OR 'inca24':ti,ab,kw OR 'inc-a24':ti,ab,kw OR 'inc a24':ti,ab,kw OR 'inc424':ti,ab,kw OR 'inc-424':ti,ab,kw OR 'inc 424':ti,ab,kw OR '941678-49-5':ti,ab,kw OR 'c17h18n6':ti,ab,kw OR 'c17h21n6o4p':ti,ab,kw OR 'tofacitinib':ti,ab,kw OR 'tasocitinib':ti,ab,kw OR 'xeljanz':ti,ab,kw OR 'jaquinus':ti,ab,kw OR 'cp-690550':ti,ab,kw OR 'cp 690550':ti,ab,kw OR 'cp690550':ti,ab,kw OR 'cp-690,550':ti,ab,kw OR 'cp 690,550':ti,ab,kw OR 'cp690,550':ti,ab,kw OR '477600-75-2':ti,ab,kw OR 'c16h20n6o':ti,ab,kw OR 'oclacitinib':ti,ab,kw OR 'apoquel':ti,ab,kw OR 'pf 03394197':ti,ab,kw OR 'pf-03394197':ti,ab,kw OR 'pf03394197':ti,ab,kw OR '1208319-26-9':ti,ab,kw OR 'c15h23n5o2s':ti,ab,kw OR 'baricitinib':ti,ab,kw OR 'olumiant':ti,ab,kw OR 'incb 028050':ti,ab,kw OR 'incb-028050':ti,ab,kw OR 'incb028050':ti,ab,kw OR 'incb 28050':ti,ab,kw OR 'incb-28050':ti,ab,kw OR 'incb28050':ti,ab,kw OR 'ly 3009104':ti,ab,kw OR 'ly-3009104':ti,ab,kw OR 'ly3009104':ti,ab,kw OR '1187594-09-7':ti,ab,kw OR 'c16h17n7o2s':ti,ab,kw OR 'peficitinib':ti,ab,kw OR 'smyraf':ti,ab,kw OR 'asp 015k':ti,ab,kw OR 'asp-015k':ti,ab,kw OR 'asp015k':ti,ab,kw OR 'jnj 54781532':ti,ab,kw OR 'jnj-54781532':ti,ab,kw OR 'jnj54781532':ti,ab,kw OR '944118-01-8':ti,ab,kw OR 'c18h22n4o2':ti,ab,kw OR 'upadacitinib':ti,ab,kw OR 'rinvoq':ti,ab,kw OR 'abt 494':ti,ab,kw OR 'abt-494':ti,ab,kw OR 'abt494':ti,ab,kw OR '1310726-60-3':ti,ab,kw OR 'c17h19f3n6o':ti,ab,kw OR 'fedratinib':ti,ab,kw OR 'inrebic':ti,ab,kw OR 'sar 302503':ti,ab,kw OR 'sar-302503':ti,ab,kw OR 'sar302503':ti,ab,kw OR 'tg 101348':ti,ab,kw OR 'tg-101348':ti,ab,kw OR 'tg101348':ti,ab,kw OR '936091-26-8':ti,ab,kw OR 'c27h36n6o3s':ti,ab,kw OR 'delgocitinib':ti,ab,kw OR 'corectim':ti,ab,kw OR 'jte 052':ti,ab,kw OR 'jte-052':ti,ab,kw OR 'jte052':ti,ab,kw OR 'jte 052a':ti,ab,kw OR 'jte-052a':ti,ab,kw OR 'jte052a':ti,ab,kw OR 'leo 124249':ti,ab,kw OR 'leo-124249':ti,ab,kw OR 'leo124249':ti,ab,kw OR 'leo 124249a':ti,ab,kw OR 'leo-124249a':ti,ab,kw OR 'leo124249a':ti,ab,kw OR '1263774-59-9':ti,ab,kw OR 'c16h18n6o':ti,ab,kw OR 'filgotinib':ti,ab,kw OR 'jyseleca':ti,ab,kw OR 'gs 6034':ti,ab,kw OR 'gs-6034':ti,ab,kw OR 'gs6034':ti,ab,kw OR 'glpg 0634':ti,ab,kw OR 'glpg-0634':ti,ab,kw OR 'glpg0634':ti,ab,kw OR 'g 146034':ti,ab,kw OR 'g-146034':ti,ab,kw OR 'g146034':ti,ab,kw OR '1206101-20-3':ti,ab,kw OR '1206161-97-8':ti,ab,kw OR 'c21h23n5o3s':ti,ab,kw OR 'abrocitinib':ti,ab,kw OR 'cibinqo':ti,ab,kw OR 'pf 04965842':ti,ab,kw OR 'pf-04965842':ti,ab,kw OR 'pf04965842':ti,ab,kw OR '1622902-68-4':ti,ab,kw OR 'c14h21n5o2s':ti,ab,kw OR 'pacritinib':ti,ab,kw OR 'vonjo':ti,ab,kw OR 'sb 1518':ti,ab,kw OR 'sb-1518':ti,ab,kw OR 'sb1518':ti,ab,kw OR '937272-79-2':ti,ab,kw OR 'c28h32n4o3':ti,ab,kw OR 'deucravacitinib':ti,ab,kw OR 'sotyktu':ti,ab,kw OR 'bms 986165':ti,ab,kw OR 'bms-986165':ti,ab,kw OR 'bms986165':ti,ab,kw OR 'tyk2-in-4':ti,ab,kw OR 'tyk2 in 4':ti,ab,kw OR 'tyk2in4':ti,ab,kw OR '1609392-27-9':ti,ab,kw OR 'c20h22n8o3':ti,ab,kw OR 'cerdulatinib':ti,ab,kw OR 'prt 2070':ti,ab,kw OR 'prt-2070':ti,ab,kw OR 'prt2070':ti,ab,kw OR 'prt 062070':ti,ab,kw OR 'prt-062070':ti,ab,kw OR 'prt062070':ti,ab,kw OR 'dmvt 502':ti,ab,kw OR 'dmvt-502':ti,ab,kw OR 'dmvt502':ti,ab,kw OR 'rvt 502':ti,ab,kw OR 'rvt-502':ti,ab,kw OR 'rvt502':ti,ab,kw OR '1198300-79-6':ti,ab,kw OR 'c20h27n7o3s':ti,ab,kw OR 'gandotinib':ti,ab,kw OR 'ly 2784544':ti,ab,kw OR 'ly-2784544':ti,ab,kw OR 'ly2784544':ti,ab,kw OR '1229236-86-5':ti,ab,kw OR 'c23h25clfn7o':ti,ab,kw OR 'lestaurtinib':ti,ab,kw OR 'cep 701':ti,ab,kw OR 'cep-701':ti,ab,kw OR 'cep701':ti,ab,kw OR 'kt 5555':ti,ab,kw OR 'kt-5555':ti,ab,kw OR 'kt5555':ti,ab,kw OR 'spm 924':ti,ab,kw OR 'spm-924':ti,ab,kw OR 'spm924':ti,ab,kw OR 'kt 555':ti,ab,kw OR 'kt-555':ti,ab,kw OR 'kt555':ti,ab,kw OR '111358-88-4':ti,ab,kw OR 'c26h21n3o4':ti,ab,kw OR 'momelotinib':ti,ab,kw OR 'cyt 387':ti,ab,kw OR 'cyt-387':ti,ab,kw OR 'cyt387':ti,ab,kw OR 'cyt 11387':ti,ab,kw OR 'cyt-11387':ti,ab,kw OR 'cyt11387':ti,ab,kw OR 'gs-0387':ti,ab,kw OR 'gs 0387':ti,ab,kw OR 'gs0387':ti,ab,kw OR 'gs-387':ti,ab,kw OR 'gs 387':ti,ab,kw OR 'gs387':ti,ab,kw OR 'lm 1149':ti,ab,kw OR 'lm-1149':ti,ab,kw OR 'lm1149':ti,ab,kw OR '1056634-68-4':ti,ab,kw OR 'cucurbitacin':ti,ab,kw OR 'elatericin':ti,ab,kw OR 'jsi 124':ti,ab,kw OR 'jsi-124':ti,ab,kw OR 'jsi124':ti,ab,kw OR 'nsc 521777':ti,ab,kw OR 'nsc-521777':ti,ab,kw OR 'nsc521777':ti,ab,kw OR 'nsc 112167':ti,ab,kw OR 'nsc-112167':ti,ab,kw OR 'nsc112167':ti,ab,kw OR '2222-07-3':ti,ab,kw OR 'c30h42o7':ti,ab,kw OR 'chz 868':ti,ab,kw OR 'chz-868':ti,ab,kw OR 'chz868':ti,ab,kw OR 'c22h19f2n5o2':ti,ab,kw OR 'tumor necrosis factor inhib*':ti,ab,kw OR 'tumor necrosis factor-a inhib*':ti,ab,kw OR 'tumor necrosis factor-α inhib*':ti,ab,kw OR 'tumor necrosis factor-alpha inhib*':ti,ab,kw OR 'tumor necrosis factor a inhib*':ti,ab,kw OR 'tumor necrosis factor α inhib*':ti,ab,kw OR 'tumor necrosis factor alpha inhib*':ti,ab,kw OR 'tumor necrosis factor antag*':ti,ab,kw OR 'tumor necrosis factor-a antag*':ti,ab,kw OR 'tumor necrosis factor-α antag*':ti,ab,kw OR 'tumor necrosis factor-alpha antag*':ti,ab,kw OR 'tumor necrosis factor a antag*':ti,ab,kw OR 'tumor necrosis factor α antag*':ti,ab,kw OR 'tumor necrosis factor alpha antag*':ti,ab,kw OR 'tumor necrosis factor block*':ti,ab,kw OR 'tumor necrosis factor-a block*':ti,ab,kw OR 'tumor necrosis factor-α block*':ti,ab,kw OR 'tumor necrosis factor-alpha block*':ti,ab,kw OR 'tumor necrosis factor a block*':ti,ab,kw OR 'tumor necrosis factor α block*':ti,ab,kw OR 'tumor necrosis factor alpha block*':ti,ab,kw OR 'tumour necrosis factor inhib*':ti,ab,kw OR 'tumour necrosis factor-a inhib*':ti,ab,kw OR 'tumour necrosis factor-α inhib*':ti,ab,kw OR 'tumour necrosis factor-alpha inhib*':ti,ab,kw OR 'tumour necrosis factor a inhib*':ti,ab,kw OR 'tumour necrosis factor α inhib*':ti,ab,kw OR 'tumour necrosis factor alpha inhib*':ti,ab,kw OR 'tumour necrosis factor antag*':ti,ab,kw OR 'tumour necrosis factor-a antag*':ti,ab,kw OR 'tumour necrosis factor-α antag*':ti,ab,kw OR 'tumour necrosis factor-alpha antag*':ti,ab,kw OR 'tumour necrosis factor a antag*':ti,ab,kw OR 'tumour necrosis factor α antag*':ti,ab,kw OR 'tumour necrosis factor alpha antag*':ti,ab,kw OR 'tumour necrosis factor block*':ti,ab,kw OR 'tumour necrosis factor-a block*':ti,ab,kw OR 'tumour necrosis factor-α block*':ti,ab,kw OR 'tumour necrosis factor-alpha block*':ti,ab,kw OR 'tumour necrosis factor a block*':ti,ab,kw OR 'tumour necrosis factor α block*':ti,ab,kw OR 'tumour necrosis factor alpha block*':ti,ab,kw OR 'tnf inhib*':ti,ab,kw OR 'tnf-a inhib*':ti,ab,kw OR 'tnf-α inhib*':ti,ab,kw OR 'tnf-alpha inhib*':ti,ab,kw OR 'tnf a inhib*':ti,ab,kw OR 'tnf α inhib*':ti,ab,kw OR 'tnf alpha inhib*':ti,ab,kw OR 'tnf antag*':ti,ab,kw OR 'tnf-a antag*':ti,ab,kw OR 'tnf-α antag*':ti,ab,kw OR 'tnf-alpha antag*':ti,ab,kw OR 'tnf a antag*':ti,ab,kw OR 'tnf α antag*':ti,ab,kw OR 'tnf alpha antag*':ti,ab,kw OR 'tnf block*':ti,ab,kw OR 'tnf-a block*':ti,ab,kw OR 'tnf-α block*':ti,ab,kw OR 'tnf-alpha block*':ti,ab,kw OR 'tnf a block*':ti,ab,kw OR 'tnf α block*':ti,ab,kw OR 'tnf alpha block*':ti,ab,kw OR 'anti-tumour necrosis factor*':ti,ab,kw OR 'anti-tnf*':ti,ab,kw OR 'cachectin inhib*':ti,ab,kw OR 'cachectin antag*':ti,ab,kw OR 'cachectin block*':ti,ab,kw OR 'infliximab':ti,ab,kw OR 'remicade':ti,ab,kw OR 'inflectra':ti,ab,kw OR 'avsola':ti,ab,kw OR 'ixifi':ti,ab,kw OR 'renflexis':ti,ab,kw OR 'mab ca2':ti,ab,kw OR 'monoclonal antibody ca2':ti,ab,kw OR 'ta-650':ti,ab,kw OR 'ta 650':ti,ab,kw OR 'ta650':ti,ab,kw OR '170277-31-3':ti,ab,kw OR 'adalimumab':ti,ab,kw OR 'humira':ti,ab,kw OR 'abrilada':ti,ab,kw OR 'amgevita':ti,ab,kw OR 'amjevita':ti,ab,kw OR 'amsparity':ti,ab,kw OR 'ardalicip':ti,ab,kw OR 'cadalimab':ti,ab,kw OR 'ciptunec':ti,ab,kw OR 'cyltezo':ti,ab,kw OR 'exemptia':ti,ab,kw OR 'hadlima':ti,ab,kw OR 'halimatoz':ti,ab,kw OR 'hefiya':ti,ab,kw OR 'hukyndra':ti,ab,kw OR 'hulio':ti,ab,kw OR 'hyrimoz':ti,ab,kw OR 'idacio':ti,ab,kw OR 'imraldi':ti,ab,kw OR 'kromeya':ti,ab,kw OR 'libmyris':ti,ab,kw OR 'mabura':ti,ab,kw OR 'simlandi':ti,ab,kw OR 'solymbic':ti,ab,kw OR 'trudexa':ti,ab,kw OR 'yuflyma':ti,ab,kw OR 'yusimry':ti,ab,kw OR 'd2e7':ti,ab,kw OR '331731-18-1':ti,ab,kw OR 'c6428h9912n1694o1987s46':ti,ab,kw OR 'etanercept':ti,ab,kw OR 'enbrel':ti,ab,kw OR 'erelzi':ti,ab,kw OR 'eticovo':ti,ab,kw OR 'benepali':ti,ab,kw OR 'tnfr-fc':ti,ab,kw OR 'tnfr fc':ti,ab,kw OR 'tnt receptor fusion protein':ti,ab,kw OR 'tnf receptor type ii-igg fusion protein':ti,ab,kw OR 'tnf receptor type ii igg fusion protein':ti,ab,kw OR 'tnr 001':ti,ab,kw OR 'tnr-001':ti,ab,kw OR 'tnr001':ti,ab,kw OR '185243-69-0':ti,ab,kw OR 'c2224h3475n621o698s36':ti,ab,kw OR 'golimumab':ti,ab,kw OR 'cnto-148':ti,ab,kw OR 'cnto 148':ti,ab,kw OR 'cnto148':ti,ab,kw OR '476181-74-5':ti,ab,kw OR 'c6530h10068n1752o2026s44':ti,ab,kw OR 'certolizumab':ti,ab,kw OR 'cimzia':ti,ab,kw OR 'cdp 870':ti,ab,kw OR 'cdp-870':ti,ab,kw OR 'cdp870':ti,ab,kw OR '428863-50-7':ti,ab,kw OR 'c2115h3252n556o673s16':ti,ab,kw OR 'thalidomide':ti,ab,kw OR 'thalomid*':ti,ab,kw OR 'algosediv':ti,ab,kw OR 'asmadion':ti,ab,kw OR 'asmaval':ti,ab,kw OR 'bonbrain':ti,ab,kw OR 'calmore':ti,ab,kw OR 'calmorex':ti,ab,kw OR 'contergan':ti,ab,kw OR 'corronarobetin':ti,ab,kw OR 'distaval':ti,ab,kw OR 'ectiluran':ti,ab,kw OR 'enterosediv':ti,ab,kw OR 'gastrinide':ti,ab,kw OR 'glupan':ti,ab,kw OR 'glutanon':ti,ab,kw OR 'grippex':ti,ab,kw OR 'hippuzon':ti,ab,kw OR 'imidene':ti,ab,kw OR 'isomin':ti,ab,kw OR 'kevadon':ti,ab,kw OR 'neosedyn':ti,ab,kw OR 'nerosedyn':ti,ab,kw OR 'neufatin':ti,ab,kw OR 'neurodyn':ti,ab,kw OR 'neurosedin':ti,ab,kw OR 'neurosedym':ti,ab,kw OR 'nevrodyn':ti,ab,kw OR 'nibrol':ti,ab,kw OR 'noctosediv':ti,ab,kw OR 'noxodyn':ti,ab,kw OR 'pangul':ti,ab,kw OR 'pantosediv':ti,ab,kw OR 'phthalimidoglut':ti,ab,kw OR 'phthaloylglut':ti,ab,kw OR 'phthalylglut':ti,ab,kw OR 'polygripan':ti,ab,kw OR 'profarmil':ti,ab,kw OR 'psycholiquid':ti,ab,kw OR 'psychotablet':ti,ab,kw OR 'quetimid':ti,ab,kw OR 'quietoplex':ti,ab,kw OR 'sandormin':ti,ab,kw OR 'sedimide':ti,ab,kw OR 'sedisperil':ti,ab,kw OR 'sedoval':ti,ab,kw OR 'shinnibrol':ti,ab,kw OR 'sleepan':ti,ab,kw OR 'slipro':ti,ab,kw OR 'softenil':ti,ab,kw OR 'softenon':ti,ab,kw OR 'talargan':ti,ab,kw OR 'talimol':ti,ab,kw OR 'talismol':ti,ab,kw OR 'telagan':ti,ab,kw OR 'telargean':ti,ab,kw OR 'tensival':ti,ab,kw OR 'thalin':ti,ab,kw OR 'thalinette':ti,ab,kw OR 'theophilcholine':ti,ab,kw OR 'valgis':ti,ab,kw OR 'valgraine':ti,ab,kw OR '50-35-1':ti,ab,kw OR 'c13h10n2o4':ti,ab,kw OR 'lenalidomide':ti,ab,kw OR 'revlimid':ti,ab,kw OR 'linamide':ti,ab,kw OR 'revamid':ti,ab,kw OR 'revimid':ti,ab,kw OR 'cc 5013':ti,ab,kw OR 'cc-5013':ti,ab,kw OR 'cc5013':ti,ab,kw OR 'cdc 501':ti,ab,kw OR 'cdc-501':ti,ab,kw OR 'cdc501':ti,ab,kw OR 'imid3':ti,ab,kw OR 'imid-5013':ti,ab,kw OR 'imid 5013':ti,ab,kw OR 'imid5013':ti,ab,kw OR 'enmd-0997':ti,ab,kw OR 'enmd 0997':ti,ab,kw OR 'enmd0997':ti,ab,kw OR '191732-72-6':ti,ab,kw OR 'c13h13n3o3':ti,ab,kw OR 'nafamostat':ti,ab,kw OR 'simponi':ti,ab,kw OR 'ronastat':ti,ab,kw OR 'ckd-314':ti,ab,kw OR 'ckd 314':ti,ab,kw OR 'ckd314':ti,ab,kw OR 'fut-175':ti,ab,kw OR 'fut 175':ti,ab,kw OR 'fut175':ti,ab,kw OR '81525-10-2':ti,ab,kw OR 'c19h17n5o2':ti,ab,kw OR 'pentoxifylline':ti,ab,kw OR 'oxpentifylline':ti,ab,kw OR 'trental':ti,ab,kw OR 'torental':ti,ab,kw OR 'pentoxil':ti,ab,kw OR 'dimethyloxohexylxanthine':ti,ab,kw OR 'agapurin':ti,ab,kw OR 'azupentat':ti,ab,kw OR 'durapental':ti,ab,kw OR 'hemovas':ti,ab,kw OR 'ikomio':ti,ab,kw OR 'pentoxifil':ti,ab,kw OR 'ralofect':ti,ab,kw OR 'rentylin':ti,ab,kw OR 'vasofirin':ti,ab,kw OR 'vazofirin':ti,ab,kw OR 'pentoxin':ti,ab,kw OR 'bl 191':ti,ab,kw OR 'bl-191':ti,ab,kw OR 'bl191':ti,ab,kw OR '6493-05-6':ti,ab,kw OR 'c13h18n4o3':ti,ab,kw OR 'abp 501':ti,ab,kw OR 'abp-501':ti,ab,kw OR 'abp501':ti,ab,kw OR 'ajulemic':ti,ab,kw OR 'lenabasum':ti,ab,kw OR 'resunab':ti,ab,kw OR 'cervelo':ti,ab,kw OR 'anabasum':ti,ab,kw OR 'ip-751':ti,ab,kw OR 'ip 751':ti,ab,kw OR 'ip751':ti,ab,kw OR 'cpl-7075':ti,ab,kw OR 'cpl 7075':ti,ab,kw OR 'cpl7075':ti,ab,kw OR 'ct-3':ti,ab,kw OR 'ct 3':ti,ab,kw OR 'ct3':ti,ab,kw OR '137945-48-3':ti,ab,kw OR 'golnerminogene':ti,ab,kw OR 'tnferade':ti,ab,kw OR '957472-14-9':ti,ab,kw OR 'ad5fgf-4':ti,ab,kw OR 'ad5fgf 4':ti,ab,kw OR 'ad5fgf4':ti,ab,kw OR 'pf-06410293':ti,ab,kw OR 'pf 06410293':ti,ab,kw OR 'pf06410293':ti,ab,kw OR 'pf-06438179':ti,ab,kw OR 'pf 06438179':ti,ab,kw OR 'pf06438179':ti,ab,kw OR 'gp 1111':ti,ab,kw OR 'gp-1111':ti,ab,kw OR 'gp1111':ti,ab,kw OR 'remtolumab':ti,ab,kw OR 'abt-122':ti,ab,kw OR 'abt 122':ti,ab,kw OR 'abt122':ti,ab,kw OR '1791410-27-9':ti,ab,kw OR 'an0128':ti,ab,kw OR 'an 0128':ti,ab,kw OR 'an-0128':ti,ab,kw OR 'crm-0005':ti,ab,kw OR 'crm 0005':ti,ab,kw OR 'crm0005':ti,ab,kw OR '872044-70-7':ti,ab,kw OR 'apeptico':ti,ab,kw OR 'ap-301':ti,ab,kw OR 'ap 301':ti,ab,kw OR 'ap301':ti,ab,kw OR 'baicalein':ti,ab,kw OR 'noroxylin':ti,ab,kw OR 'baikalein':ti,ab,kw OR 'baicelein':ti,ab,kw OR 'baikelein':ti,ab,kw OR 'nsc661431':ti,ab,kw OR 'nsc 661431':ti,ab,kw OR 'nsc-661431':ti,ab,kw OR 'sho-saiko-to':ti,ab,kw OR '491-67-8':ti,ab,kw OR 'c15h10o5':ti,ab,kw OR 'delenex':ti,ab,kw OR 'dlx-105':ti,ab,kw OR 'dlx 105':ti,ab,kw OR 'dlx105':ti,ab,kw OR 'esba-105':ti,ab,kw OR 'esba 105':ti,ab,kw OR 'esba105':ti,ab,kw OR '1142149-71-0':ti,ab,kw OR 'ortataxel':ti,ab,kw OR '8h61y4e29n':ti,ab,kw OR 'idn-5109':ti,ab,kw OR 'idn 5109':ti,ab,kw OR 'idn5109':ti,ab,kw OR 'bay-59*':ti,ab,kw OR 'bay 59*':ti,ab,kw OR 'bay59*':ti,ab,kw OR 'hexanoic acid':ti,ab,kw OR 'genz 29155':ti,ab,kw OR 'genz-29155':ti,ab,kw OR 'genz29155':ti,ab,kw OR 'sb-t-101131':ti,ab,kw OR 'sb t 101131':ti,ab,kw OR 'sbt101131':ti,ab,kw OR '186348-23-2':ti,ab,kw OR 'c44h57no17':ti,ab,kw OR 'pegsunercept':ti,ab,kw OR 'pegylated soluble tumor necrosis factor receptor i':ti,ab,kw OR 'peg stnfri':ti,ab,kw OR 'stnf-ri':ti,ab,kw OR 'stnf ri':ti,ab,kw OR 'stnfri':ti,ab,kw OR 'stnf-r1':ti,ab,kw OR 'stnf r1':ti,ab,kw OR 'stnfr1':ti,ab,kw OR '330988-75-5':ti,ab,kw OR 'c502h758n154o165s16':ti,ab,kw OR 'allotrap':ti,ab,kw OR 'delmitide':ti,ab,kw OR 'peptide bc-1nl':ti,ab,kw OR 'rdp 58':ti,ab,kw OR 'rdp-58':ti,ab,kw OR 'rdp58':ti,ab,kw OR 'rsp 58':ti,ab,kw OR 'rsp-58':ti,ab,kw OR 'rsp58':ti,ab,kw OR '287096-87-1':ti,ab,kw OR 'c59h105n17o11':ti,ab,kw OR 'kinoid':ti,ab,kw OR 'neovacs':ti,ab,kw OR 'debio-0512':ti,ab,kw OR 'debio 0512':ti,ab,kw OR 'debio0512':ti,ab,kw OR 'cova322':ti,ab,kw OR 'cova-322':ti,ab,kw OR 'cova 322':ti,ab,kw OR 'gtpl10469':ti,ab,kw OR 'gtpl 10469':ti,ab,kw OR 'gtpl-10469':ti,ab,kw OR 'placulumab':ti,ab,kw OR 'pn-0621':ti,ab,kw OR 'pn 0621':ti,ab,kw OR 'pn0621':ti,ab,kw OR 'cep-37247':ti,ab,kw OR 'cep 37247':ti,ab,kw OR 'cep37247':ti,ab,kw OR 'art621':ti,ab,kw OR 'art 621':ti,ab,kw OR 'art-621':ti,ab,kw OR 'abbv-257':ti,ab,kw OR 'abbv 257':ti,ab,kw OR 'abbv257':ti,ab,kw OR 'ma59o0rpes':ti,ab,kw OR 'ast-005':ti,ab,kw OR 'ast 005':ti,ab,kw OR 'ast005':ti,ab,kw OR 'avx-470':ti,ab,kw OR 'avx 470':ti,ab,kw OR 'avx470':ti,ab,kw OR 'aurimune':ti,ab,kw OR 'cyt-609':ti,ab,kw OR 'cyt 609':ti,ab,kw OR 'cyt609':ti,ab,kw OR '877170-68-8':ti,ab,kw OR 'inb03':ti,ab,kw OR 'inb 03':ti,ab,kw OR 'inb-03':ti,ab,kw OR 'pf-05230905':ti,ab,kw OR 'pf 05230905':ti,ab,kw OR 'pf05230905':ti,ab,kw OR 'atn-192':ti,ab,kw OR 'atn 192':ti,ab,kw OR 'atn192':ti,ab,kw OR 'pmi-005':ti,ab,kw OR 'pmi 005':ti,ab,kw OR 'pmi005':ti,ab,kw OR 'abx-0401':ti,ab,kw OR 'abx 0401':ti,ab,kw OR 'abx0401':ti,ab,kw OR 'alx-0071':ti,ab,kw OR 'alx 0071':ti,ab,kw OR 'alx0071':ti,ab,kw OR 'celastrol':ti,ab,kw OR 'tripterin':ti,ab,kw OR 'celastrus scandens':ti,ab,kw OR '34157-83-0':ti,ab,kw OR 'c29h38o4':ti,ab,kw OR 'cdp571':ti,ab,kw OR 'cdp 571':ti,ab,kw OR 'cdp-571':ti,ab,kw OR 'afelimomab':ti,ab,kw OR 'segard':ti,ab,kw OR 'mak-195f':ti,ab,kw OR 'mak 195f':ti,ab,kw OR 'mak195f':ti,ab,kw OR 'lu-54107':ti,ab,kw OR 'lu 54107':ti,ab,kw OR 'lu54107':ti,ab,kw OR '156227-98-4':ti,ab,kw OR 'camobucol':ti,ab,kw OR 'ylgucpd100bdh':ti,ab,kw OR 'v-protectant':ti,ab,kw OR 'agix-4207':ti,ab,kw OR 'agix 4207':ti,ab,kw OR 'agix4207':ti,ab,kw OR '216167-92-9':ti,ab,kw OR 'c33h50o4s2':ti,ab,kw OR 'crx-191':ti,ab,kw OR 'crx 191':ti,ab,kw OR 'crx191':ti,ab,kw OR 'ame-527':ti,ab,kw OR 'ame 527':ti,ab,kw OR 'ame527':ti,ab,kw OR '1001159-86-9':ti,ab,kw OR 'cyt-007-tnfqb':ti,ab,kw OR 'cyt 007 tnfqb':ti,ab,kw OR 'cyt007tnfqb':ti,ab,kw OR 'als-00t2-0501':ti,ab,kw OR 'als 00t2 0501':ti,ab,kw OR 'als00t20501':ti,ab,kw OR 'fr-133605':ti,ab,kw OR 'fr 133605':ti,ab,kw OR 'fr133605':ti,ab,kw OR 'mdl-201112':ti,ab,kw OR 'mdl 201112':ti,ab,kw OR 'mdl201112':ti,ab,kw OR 'mdl-201449':ti,ab,kw OR 'mdl 201449':ti,ab,kw OR 'mdl201449':ti,ab,kw OR 'mdl-201449a':ti,ab,kw OR 'mdl 201449a':ti,ab,kw OR 'mdl201449a':ti,ab,kw OR '142130-73-2':ti,ab,kw OR 'c10h13n5o':ti,ab,kw OR 'carbocyclic nucleoside':ti,ab,kw) AND ('alopecia cicatrisata*':ti,ab,kw OR 'cicatricial alopeci*':ti,ab,kw OR 'scarring alopeci*':ti,ab,kw OR 'irreversible alopeci*':ti,ab,kw OR 'permanent alopeci*':ti,ab,kw OR 'cicatricial bald*':ti,ab,kw OR 'scarring bald*':ti,ab,kw OR 'irreversible bald*':ti,ab,kw OR 'permanent bald*':ti,ab,kw OR 'cicatricial hair los*':ti,ab,kw OR 'scarring hair los*':ti,ab,kw OR 'irreversible hair los*':ti,ab,kw OR 'permanent hair los*':ti,ab,kw OR 'pseudopelad*':ti,ab,kw OR 'brocq':ti,ab,kw OR 'scarring lichen plan*':ti,ab,kw OR 'scalp lichen plan*':ti,ab,kw OR 'lichen planopilar*':ti,ab,kw OR 'coup de sabre*':ti,ab,kw OR 'discoid lupus*':ti,ab,kw OR 'folliculitis decalvans':ti,ab,kw OR 'erosive pustular dermatosis':ti,ab,kw OR 'chronic atrophic dermatosis':ti,ab,kw OR 'frontal fibrosing alopecia':ti,ab,kw OR 'central centrifugal alopecia':ti,ab,kw OR 'central centrifugal cicatricial alopecia':ti,ab,kw OR 'tufted folliculitis':ti,ab,kw OR 'dissecting cellulitis':ti,ab,kw OR 'perifolliculitis capitis':ti,ab,kw OR 'folliculitis keloidalis':ti,ab,kw OR 'acne cheloidalis nuch*':ti,ab,kw OR 'acne keloidalis':ti,ab,kw OR 'papular atrichia':ti,ab,kw OR 'atrichia with papular lesion*':ti,ab,kw) | 300 |
| Google Scholar | Search No. 1:  (“Alopecia Cicatrisata" OR "Cicatricial Alopecia" OR "Scarring Alopecia") AND (Janus Kinase Inhibitor OR JAK inhibitor OR Tumor Necrosis Factor inhibitor OR TNF inhibitor OR TNF blocker Or Cachectin inhibitor) | No results |
|  | Search No. 2:  (“Alopecia Cicatrisata" OR "Cicatricial Alopecia" OR "Scarring Alopecia") AND (Ruxolitinib OR Tofacitinib OR Tasocitinib OR Baricitinib OR Upadacitinib OR Fedratinib OR Abrocitinib OR Pacritinib OR Deucravacitinib) | 449  (Screening the first 100 results) |
|  | Search No. 3:  (“Alopecia Cicatrisata" OR "Cicatricial Alopecia" OR "Scarring Alopecia") AND (Infliximab OR Adalimumab OR Etanercept OR Golimumab OR Certolizumab OR Thalidomide OR Lenalidomide OR Nafamostat OR Pentoxifylline) | 1,850  (Screening the first 100 results) |
| PubMed | ("Janus Kinase Inhibitors"[Mesh] OR "Tyrosine Protein Kinase Inhibitors"[Mesh] OR "Cucurbitacins"[Mesh] OR "Baricitinib"[Supplementary Concept] OR "Oclacitinib"[Supplementary Concept] OR "Ruxolitinib"[Supplementary Concept] OR "Tofacitinib"[Supplementary Concept] OR "Peficitinib"[Supplementary Concept] OR "Upadacitinib"[Supplementary Concept] OR "Fedratinib"[Supplementary Concept] OR "Delgocitinib"[Supplementary Concept] OR "GLPG0634"[Supplementary Concept] OR "Abrocitinib"[Supplementary Concept] OR "11-(2-pyrrolidin-1-ylethoxy)-14,19-dioxa-5,7,26-triazatetracyclo(19.3.1.1(2,6).1(8,12))heptacosa-1(25),2(26),3,5,8,10,12(27),16,21,23-decaene"[Supplementary Concept] OR "Deucravacitinib"[Supplementary Concept] OR "4-(cyclopropylamino)-2-((4-(4-(ethylsulfonyl)piperazin-1-yl)phenyl)amino)pyrimidine-5-carboxamide"[Supplementary Concept] OR "Lestaurtinib"[Supplementary Concept] OR "N-(cyanomethyl)-4-(2-((4-(4-morpholinyl)phenyl)amino)-4-pyrimidinyl)benzamide"[Supplementary Concept] OR "*JAK inhibit*"[Title/Abstract] OR "*Jakinib*"[Title/Abstract] OR "*Janus kinase inhibit*"[Title/Abstract] OR "*Tyrosine kinase inhibit*"[Title/Abstract] OR "*JAK-STAT inhibit*"[Title/Abstract] OR "*JAKi*"[Title/Abstract] OR "*JAK i*"[Title/Abstract] OR "*JAK-i*"[Title/Abstract] OR "*JAK1 inhibit*"[Title/Abstract] OR "*JAK2 inhibit*"[Title/Abstract] OR "*JAK3 inhibit*"[Title/Abstract] OR "*TYK2 inhibit*"[Title/Abstract] OR "*Janus kinase 1 inhibit*"[Title/Abstract] OR "*Janus kinase 2 inhibit*"[Title/Abstract] OR "*Janus kinase 3 inhibit*"[Title/Abstract] OR "*Tyrosine kinase 2 inhibit*"[Title/Abstract] OR "Ruxolitinib"[Title/Abstract] OR "Jakafi"[Title/Abstract] OR "Jakavi"[Title/Abstract] OR "Opzelura"[Title/Abstract] OR "INCB018424"[Title/Abstract] OR "INCB-018424"[Title/Abstract] OR "INCB 018424"[Title/Abstract] OR "INCB18424"[Title/Abstract] OR "INCB-18424"[Title/Abstract] OR "INCB 18424"[Title/Abstract] OR "INCA24"[Title/Abstract] OR "INC-A24"[Title/Abstract] OR "INC A24"[Title/Abstract] OR "INC424"[Title/Abstract] OR "INC-424"[Title/Abstract] OR "INC 424"[Title/Abstract] OR "941678-49-5"[Title/Abstract] OR "C17H18N6"[Title/Abstract] OR "C17H21N6O4P"[Title/Abstract] OR "Tofacitinib"[Title/Abstract] OR "Tasocitinib"[Title/Abstract] OR "Xeljanz"[Title/Abstract] OR "Jaquinus"[Title/Abstract] OR "CP-690550"[Title/Abstract] OR "CP 690550"[Title/Abstract] OR "CP690550"[Title/Abstract] OR "CP-690,550"[Title/Abstract] OR "CP 690,550"[Title/Abstract] OR "CP690,550"[Title/Abstract] OR "477600-75-2"[Title/Abstract] OR "C16H20N6O"[Title/Abstract] OR "Oclacitinib"[Title/Abstract] OR "Apoquel"[Title/Abstract] OR "PF 03394197"[Title/Abstract] OR "PF-03394197"[Title/Abstract] OR "PF03394197"[Title/Abstract] OR "1208319-26-9"[Title/Abstract] OR "C15H23N5O2S"[Title/Abstract] OR "Baricitinib"[Title/Abstract] OR "Olumiant"[Title/Abstract] OR "INCB 028050"[Title/Abstract] OR "INCB-028050"[Title/Abstract] OR "INCB028050"[Title/Abstract] OR "INCB 28050"[Title/Abstract] OR "INCB-28050"[Title/Abstract] OR "INCB28050"[Title/Abstract] OR "LY 3009104"[Title/Abstract] OR "LY-3009104"[Title/Abstract] OR "LY3009104"[Title/Abstract] OR "1187594-09-7"[Title/Abstract] OR "C16H17N7O2S"[Title/Abstract] OR "Peficitinib"[Title/Abstract] OR "Smyraf"[Title/Abstract] OR "ASP 015K"[Title/Abstract] OR "ASP-015K"[Title/Abstract] OR "ASP015K"[Title/Abstract] OR "JNJ 54781532"[Title/Abstract] OR "JNJ-54781532"[Title/Abstract] OR "JNJ54781532"[Title/Abstract] OR "944118-01-8"[Title/Abstract] OR "C18H22N4O2"[Title/Abstract] OR "Upadacitinib"[Title/Abstract] OR "Rinvoq"[Title/Abstract] OR "ABT 494"[Title/Abstract] OR "ABT-494"[Title/Abstract] OR "ABT494"[Title/Abstract] OR "1310726-60-3"[Title/Abstract] OR "C17H19F3N6O"[Title/Abstract] OR "Fedratinib"[Title/Abstract] OR "Inrebic"[Title/Abstract] OR "SAR 302503"[Title/Abstract] OR "SAR-302503"[Title/Abstract] OR "SAR302503"[Title/Abstract] OR "TG 101348"[Title/Abstract] OR "TG-101348"[Title/Abstract] OR "TG101348"[Title/Abstract] OR "936091-26-8"[Title/Abstract] OR "C27H36N6O3S"[Title/Abstract] OR "Delgocitinib"[Title/Abstract] OR "Corectim"[Title/Abstract] OR "JTE 052"[Title/Abstract] OR "JTE-052"[Title/Abstract] OR "JTE052"[Title/Abstract] OR "JTE 052A"[Title/Abstract] OR "JTE-052A"[Title/Abstract] OR "JTE052A"[Title/Abstract] OR "LEO 124249"[Title/Abstract] OR "LEO-124249"[Title/Abstract] OR "LEO124249"[Title/Abstract] OR "LEO 124249A"[Title/Abstract] OR "LEO-124249A"[Title/Abstract] OR "LEO124249A"[Title/Abstract] OR "1263774-59-9"[Title/Abstract] OR "C16H18N6O"[Title/Abstract] OR "Filgotinib"[Title/Abstract] OR "Jyseleca"[Title/Abstract] OR "GS 6034"[Title/Abstract] OR "GS-6034"[Title/Abstract] OR "GS6034"[Title/Abstract] OR "GLPG 0634"[Title/Abstract] OR "GLPG-0634"[Title/Abstract] OR "GLPG0634"[Title/Abstract] OR "G 146034"[Title/Abstract] OR "G-146034"[Title/Abstract] OR "G146034"[Title/Abstract] OR "1206101-20-3"[Title/Abstract] OR "1206161-97-8"[Title/Abstract] OR "C21H23N5O3S"[Title/Abstract] OR "Abrocitinib"[Title/Abstract] OR "Cibinqo"[Title/Abstract] OR "PF 04965842"[Title/Abstract] OR "PF-04965842"[Title/Abstract] OR "PF04965842"[Title/Abstract] OR "1622902-68-4"[Title/Abstract] OR "C14H21N5O2S"[Title/Abstract] OR "Pacritinib"[Title/Abstract] OR "Vonjo"[Title/Abstract] OR "SB 1518"[Title/Abstract] OR "SB-1518"[Title/Abstract] OR "SB1518"[Title/Abstract] OR "937272-79-2"[Title/Abstract] OR "C28H32N4O3"[Title/Abstract] OR "Deucravacitinib"[Title/Abstract] OR "Sotyktu"[Title/Abstract] OR "BMS 986165"[Title/Abstract] OR "BMS-986165"[Title/Abstract] OR "BMS986165"[Title/Abstract] OR "Tyk2-IN-4"[Title/Abstract] OR "Tyk2 IN 4"[Title/Abstract] OR "Tyk2IN4"[Title/Abstract] OR "1609392-27-9"[Title/Abstract] OR "C20H22N8O3"[Title/Abstract] OR "Cerdulatinib"[Title/Abstract] OR "PRT 2070"[Title/Abstract] OR "PRT-2070"[Title/Abstract] OR "PRT2070"[Title/Abstract] OR "PRT 062070"[Title/Abstract] OR "PRT-062070"[Title/Abstract] OR "PRT062070"[Title/Abstract] OR "DMVT 502"[Title/Abstract] OR "DMVT-502"[Title/Abstract] OR "DMVT502"[Title/Abstract] OR "RVT 502"[Title/Abstract] OR "RVT-502"[Title/Abstract] OR "RVT502"[Title/Abstract] OR "1198300-79-6"[Title/Abstract] OR "C20H27N7O3S"[Title/Abstract] OR "Gandotinib"[Title/Abstract] OR "LY 2784544"[Title/Abstract] OR "LY-2784544"[Title/Abstract] OR "LY2784544"[Title/Abstract] OR "1229236-86-5"[Title/Abstract] OR "C23H25ClFN7O"[Title/Abstract] OR "Lestaurtinib"[Title/Abstract] OR "CEP 701"[Title/Abstract] OR "CEP-701"[Title/Abstract] OR "CEP701"[Title/Abstract] OR "KT 5555"[Title/Abstract] OR "KT-5555"[Title/Abstract] OR "KT5555"[Title/Abstract] OR "SPM 924"[Title/Abstract] OR "SPM-924"[Title/Abstract] OR "SPM924"[Title/Abstract] OR "KT 555"[Title/Abstract] OR "KT-555"[Title/Abstract] OR "KT555"[Title/Abstract] OR "111358-88-4"[Title/Abstract] OR "C26H21N3O4"[Title/Abstract] OR "Momelotinib"[Title/Abstract] OR "CYT 387"[Title/Abstract] OR "CYT-387"[Title/Abstract] OR "CYT387"[Title/Abstract] OR "CYT 11387"[Title/Abstract] OR "CYT-11387"[Title/Abstract] OR "CYT11387"[Title/Abstract] OR "GS-0387"[Title/Abstract] OR "GS 0387"[Title/Abstract] OR "GS0387"[Title/Abstract] OR "GS-387"[Title/Abstract] OR "GS 387"[Title/Abstract] OR "GS387"[Title/Abstract] OR "LM 1149"[Title/Abstract] OR "LM-1149"[Title/Abstract] OR "LM1149"[Title/Abstract] OR "1056634-68-4"[Title/Abstract] OR "Cucurbitacin"[Title/Abstract] OR "Elatericin"[Title/Abstract] OR "JSI 124"[Title/Abstract] OR "JSI-124"[Title/Abstract] OR "JSI124"[Title/Abstract] OR "NSC 521777"[Title/Abstract] OR "NSC-521777"[Title/Abstract] OR "NSC521777"[Title/Abstract] OR "NSC 112167"[Title/Abstract] OR "NSC-112167"[Title/Abstract] OR "NSC112167"[Title/Abstract] OR "2222-07-3"[Title/Abstract] OR "C30H42O7"[Title/Abstract] OR "CHZ 868"[Title/Abstract] OR "CHZ-868"[Title/Abstract] OR "CHZ868"[Title/Abstract] OR "C22H19F2N5O2"[Title/Abstract] OR "Tumor Necrosis Factor Inhibitors"[Mesh] OR "Infliximab"[Mesh] OR "Adalimumab"[Mesh] OR "Etanercept"[Mesh] OR "Certolizumab Pegol"[Mesh] OR "Thalidomide"[Mesh] OR "Lenalidomide"[Mesh] OR "Golimumab"[Supplementary Concept] OR "Nafamostat"[Supplementary Concept] OR "ABP 501"[Supplementary Concept] OR "Lenabasum"[Supplementary Concept] OR "PF-06410293"[Supplementary Concept] OR "GP1111"[Supplementary Concept] OR "ABT-122"[Supplementary Concept] OR "3-hydroxypyridine-2-carbonyloxy-bis(3-chloro-4-methylphenyl)borane"[Supplementary Concept] OR "Baicalein"[Supplementary Concept] OR "IDN 5109"[Supplementary Concept] OR "PEGylated soluble tumor necrosis factor receptor I"[Supplementary Concept] OR "Allotrap"[Supplementary Concept] OR "AVX-470"[Supplementary Concept] OR "Celastrol"[Supplementary Concept] OR "Afelimomab"[Supplementary Concept] OR "Camobucol"[Supplementary Concept] OR "FR 133605"[Supplementary Concept] OR "Tumor Necrosis Factor inhib*"[Title/Abstract] OR "Tumor Necrosis Factor-a inhib*"[Title/Abstract] OR "Tumor Necrosis Factor-α inhib*"[Title/Abstract] OR "Tumor Necrosis Factor-alpha inhib*"[Title/Abstract] OR "Tumor Necrosis Factor a inhib*"[Title/Abstract] OR "Tumor Necrosis Factor α inhib*"[Title/Abstract] OR "Tumor Necrosis Factor alpha inhib*"[Title/Abstract] OR "Tumor Necrosis Factor antag*"[Title/Abstract] OR "Tumor Necrosis Factor-a antag*"[Title/Abstract] OR "Tumor Necrosis Factor-α antag*"[Title/Abstract] OR "Tumor Necrosis Factor-alpha antag*"[Title/Abstract] OR "Tumor Necrosis Factor a antag*"[Title/Abstract] OR "Tumor Necrosis Factor α antag*"[Title/Abstract] OR "Tumor Necrosis Factor alpha antag*"[Title/Abstract] OR "Tumor Necrosis Factor block*"[Title/Abstract] OR "Tumor Necrosis Factor-a block*"[Title/Abstract] OR "Tumor Necrosis Factor-α block*"[Title/Abstract] OR "Tumor Necrosis Factor-alpha block*"[Title/Abstract] OR "Tumor Necrosis Factor a block*"[Title/Abstract] OR "Tumor Necrosis Factor α block*"[Title/Abstract] OR "Tumor Necrosis Factor alpha block*"[Title/Abstract] OR "Tumour Necrosis Factor inhib*"[Title/Abstract] OR "Tumour Necrosis Factor-a inhib*"[Title/Abstract] OR "Tumour Necrosis Factor-α inhib*"[Title/Abstract] OR "Tumour Necrosis Factor-alpha inhib*"[Title/Abstract] OR "Tumour Necrosis Factor a inhib*"[Title/Abstract] OR "Tumour Necrosis Factor α inhib*"[Title/Abstract] OR "Tumour Necrosis Factor alpha inhib*"[Title/Abstract] OR "Tumour Necrosis Factor antag*"[Title/Abstract] OR "Tumour Necrosis Factor-a antag*"[Title/Abstract] OR "Tumour Necrosis Factor-α antag*"[Title/Abstract] OR "Tumour Necrosis Factor-alpha antag*"[Title/Abstract] OR "Tumour Necrosis Factor a antag*"[Title/Abstract] OR "Tumour Necrosis Factor α antag*"[Title/Abstract] OR "Tumour Necrosis Factor alpha antag*"[Title/Abstract] OR "Tumour Necrosis Factor block*"[Title/Abstract] OR "Tumour Necrosis Factor-a block*"[Title/Abstract] OR "Tumour Necrosis Factor-α block*"[Title/Abstract] OR "Tumour Necrosis Factor-alpha block*"[Title/Abstract] OR "Tumour Necrosis Factor a block*"[Title/Abstract] OR "Tumour Necrosis Factor α block*"[Title/Abstract] OR "Tumour Necrosis Factor alpha block*"[Title/Abstract] OR "TNF inhib*"[Title/Abstract] OR "TNF-a inhib*"[Title/Abstract] OR "TNF-α inhib*"[Title/Abstract] OR "TNF-alpha inhib*"[Title/Abstract] OR "TNF a inhib*"[Title/Abstract] OR "TNF α inhib*"[Title/Abstract] OR "TNF alpha inhib*"[Title/Abstract] OR "TNF antag*"[Title/Abstract] OR "TNF-a antag*"[Title/Abstract] OR "TNF-α antag*"[Title/Abstract] OR "TNF-alpha antag*"[Title/Abstract] OR "TNF a antag*"[Title/Abstract] OR "TNF α antag*"[Title/Abstract] OR "TNF alpha antag*"[Title/Abstract] OR "TNF block*"[Title/Abstract] OR "TNF-a block*"[Title/Abstract] OR "TNF-α block*"[Title/Abstract] OR "TNF-alpha block*"[Title/Abstract] OR "TNF a block*"[Title/Abstract] OR "TNF α block*"[Title/Abstract] OR "TNF alpha block*"[Title/Abstract] OR "Anti-Tumour Necrosis Factor*"[Title/Abstract] OR "Anti-TNF*"[Title/Abstract] OR "Cachectin inhib*"[Title/Abstract] OR "Cachectin antag*"[Title/Abstract] OR "Cachectin block*"[Title/Abstract] OR "Infliximab"[Title/Abstract] OR "Remicade"[Title/Abstract] OR "Inflectra"[Title/Abstract] OR "Avsola"[Title/Abstract] OR "Ixifi"[Title/Abstract] OR "Renflexis"[Title/Abstract] OR "Mab cA2"[Title/Abstract] OR "Monoclonal antibody cA2"[Title/Abstract] OR "TA-650"[Title/Abstract] OR "TA 650"[Title/Abstract] OR "TA650"[Title/Abstract] OR "170277-31-3"[Title/Abstract] OR "Adalimumab"[Title/Abstract] OR "Humira"[Title/Abstract] OR "Abrilada"[Title/Abstract] OR "Amgevita"[Title/Abstract] OR "Amjevita"[Title/Abstract] OR "Amsparity"[Title/Abstract] OR "Ardalicip"[Title/Abstract] OR "Cadalimab"[Title/Abstract] OR "Ciptunec"[Title/Abstract] OR "Cyltezo"[Title/Abstract] OR "Exemptia"[Title/Abstract] OR "Hadlima"[Title/Abstract] OR "Halimatoz"[Title/Abstract] OR "Hefiya"[Title/Abstract] OR "Hukyndra"[Title/Abstract] OR "Hulio"[Title/Abstract] OR "Hyrimoz"[Title/Abstract] OR "Idacio"[Title/Abstract] OR "Imraldi"[Title/Abstract] OR "Kromeya"[Title/Abstract] OR "Libmyris"[Title/Abstract] OR "Mabura"[Title/Abstract] OR "Simlandi"[Title/Abstract] OR "Solymbic"[Title/Abstract] OR "Trudexa"[Title/Abstract] OR "Yuflyma"[Title/Abstract] OR "Yusimry"[Title/Abstract] OR "D2E7"[Title/Abstract] OR "331731-18-1"[Title/Abstract] OR "C6428H9912N1694O1987S46"[Title/Abstract] OR "Etanercept"[Title/Abstract] OR "Enbrel"[Title/Abstract] OR "Erelzi"[Title/Abstract] OR "Eticovo"[Title/Abstract] OR "Benepali"[Title/Abstract] OR "TNFR-Fc"[Title/Abstract] OR "TNFR Fc"[Title/Abstract] OR "TNT Receptor Fusion Protein"[Title/Abstract] OR "TNF Receptor Type II-IgG Fusion Protein"[Title/Abstract] OR "TNF Receptor Type II IgG Fusion Protein"[Title/Abstract] OR "TNR 001"[Title/Abstract] OR "TNR-001"[Title/Abstract] OR "TNR001"[Title/Abstract] OR "185243-69-0"[Title/Abstract] OR "C2224H3475N621O698S36"[Title/Abstract] OR "Golimumab"[Title/Abstract] OR "Simponi"[Title/Abstract] OR "CNTO-148"[Title/Abstract] OR "CNTO 148"[Title/Abstract] OR "CNTO148"[Title/Abstract] OR "476181-74-5"[Title/Abstract] OR "C6530H10068N1752O2026S44"[Title/Abstract] OR "Certolizumab"[Title/Abstract] OR "Cimzia"[Title/Abstract] OR "CDP 870"[Title/Abstract] OR "CDP-870"[Title/Abstract] OR "CDP870"[Title/Abstract] OR "428863-50-7"[Title/Abstract] OR "C2115H3252N556O673S16"[Title/Abstract] OR "Thalidomide"[Title/Abstract] OR "Thalomid*"[Title/Abstract] OR "Algosediv"[Title/Abstract] OR "Asmadion"[Title/Abstract] OR "Asmaval"[Title/Abstract] OR "Bonbrain"[Title/Abstract] OR "Calmore"[Title/Abstract] OR "Calmorex"[Title/Abstract] OR "Contergan"[Title/Abstract] OR "Corronarobetin"[Title/Abstract] OR "Distaval"[Title/Abstract] OR "Ectiluran"[Title/Abstract] OR "Enterosediv"[Title/Abstract] OR "Gastrinide"[Title/Abstract] OR "Glupan"[Title/Abstract] OR "Glutanon"[Title/Abstract] OR "Grippex"[Title/Abstract] OR "Hippuzon"[Title/Abstract] OR "Imidene"[Title/Abstract] OR "Isomin"[Title/Abstract] OR "Kevadon"[Title/Abstract] OR "Neosedyn"[Title/Abstract] OR "Nerosedyn"[Title/Abstract] OR "Neufatin"[Title/Abstract] OR "Neurodyn"[Title/Abstract] OR "Neurosedin"[Title/Abstract] OR "Neurosedym"[Title/Abstract] OR "Nevrodyn"[Title/Abstract] OR "Nibrol"[Title/Abstract] OR "Noctosediv"[Title/Abstract] OR "Noxodyn"[Title/Abstract] OR "Pangul"[Title/Abstract] OR "Pantosediv"[Title/Abstract] OR "Phthalimidoglut"[Title/Abstract] OR "Phthaloylglut"[Title/Abstract] OR "Phthalylglut"[Title/Abstract] OR "Polygripan"[Title/Abstract] OR "Profarmil"[Title/Abstract] OR "Psycholiquid"[Title/Abstract] OR "Psychotablet"[Title/Abstract] OR "Quetimid"[Title/Abstract] OR "Quietoplex"[Title/Abstract] OR "Sandormin"[Title/Abstract] OR "Sedimide"[Title/Abstract] OR "Sedisperil"[Title/Abstract] OR "Sedoval"[Title/Abstract] OR "Shinnibrol"[Title/Abstract] OR "Sleepan"[Title/Abstract] OR "Slipro"[Title/Abstract] OR "Softenil"[Title/Abstract] OR "Softenon"[Title/Abstract] OR "Talargan"[Title/Abstract] OR "Talimol"[Title/Abstract] OR "Talismol"[Title/Abstract] OR "Telagan"[Title/Abstract] OR "Telargean"[Title/Abstract] OR "Tensival"[Title/Abstract] OR "Thalin"[Title/Abstract] OR "Thalinette"[Title/Abstract] OR "Theophilcholine"[Title/Abstract] OR "Valgis"[Title/Abstract] OR "Valgraine"[Title/Abstract] OR "50-35-1"[Title/Abstract] OR "C13H10N2O4"[Title/Abstract] OR "Lenalidomide"[Title/Abstract] OR "Revlimid"[Title/Abstract] OR "Linamide"[Title/Abstract] OR "Revamid"[Title/Abstract] OR "Revimid"[Title/Abstract] OR "CC 5013"[Title/Abstract] OR "CC-5013"[Title/Abstract] OR "CC5013"[Title/Abstract] OR "CDC 501"[Title/Abstract] OR "CDC-501"[Title/Abstract] OR "CDC501"[Title/Abstract] OR "IMiD3"[Title/Abstract] OR "IMID-5013"[Title/Abstract] OR "IMID 5013"[Title/Abstract] OR "IMID5013"[Title/Abstract] OR "ENMD-0997"[Title/Abstract] OR "ENMD 0997"[Title/Abstract] OR "ENMD0997"[Title/Abstract] OR "191732-72-6"[Title/Abstract] OR "C13H13N3O3"[Title/Abstract] OR "Nafamostat"[Title/Abstract] OR "Simponi"[Title/Abstract] OR "Ronastat"[Title/Abstract] OR "CKD-314"[Title/Abstract] OR "CKD 314"[Title/Abstract] OR "CKD314"[Title/Abstract] OR "FUT-175"[Title/Abstract] OR "FUT 175"[Title/Abstract] OR "FUT175"[Title/Abstract] OR "81525-10-2"[Title/Abstract] OR "C19H17N5O2"[Title/Abstract] OR "Pentoxifylline"[Title/Abstract] OR "Oxpentifylline"[Title/Abstract] OR "Trental"[Title/Abstract] OR "Torental"[Title/Abstract] OR "Pentoxil"[Title/Abstract] OR "Dimethyloxohexylxanthine"[Title/Abstract] OR "Agapurin"[Title/Abstract] OR "Azupentat"[Title/Abstract] OR "Durapental"[Title/Abstract] OR "Hemovas"[Title/Abstract] OR "Ikomio"[Title/Abstract] OR "Pentoxifil"[Title/Abstract] OR "Ralofect"[Title/Abstract] OR "Rentylin"[Title/Abstract] OR "Vasofirin"[Title/Abstract] OR "Vazofirin"[Title/Abstract] OR "Pentoxin"[Title/Abstract] OR "BL 191"[Title/Abstract] OR "BL-191"[Title/Abstract] OR "BL191"[Title/Abstract] OR "6493-05-6"[Title/Abstract] OR "C13H18N4O3"[Title/Abstract] OR "ABP 501"[Title/Abstract] OR "ABP-501"[Title/Abstract] OR "ABP501"[Title/Abstract] OR "Ajulemic"[Title/Abstract] OR "Lenabasum"[Title/Abstract] OR "Resunab"[Title/Abstract] OR "Cervelo"[Title/Abstract] OR "Anabasum"[Title/Abstract] OR "IP-751"[Title/Abstract] OR "IP 751"[Title/Abstract] OR "IP751"[Title/Abstract] OR "CPL-7075"[Title/Abstract] OR "CPL 7075"[Title/Abstract] OR "CPL7075"[Title/Abstract] OR "CT-3"[Title/Abstract] OR "CT 3"[Title/Abstract] OR "CT3"[Title/Abstract] OR "137945-48-3"[Title/Abstract] OR "Golnerminogene"[Title/Abstract] OR "TNFerade"[Title/Abstract] OR "957472-14-9"[Title/Abstract] OR "Ad5FGF-4"[Title/Abstract] OR "Ad5FGF 4"[Title/Abstract] OR "Ad5FGF4"[Title/Abstract] OR "PF-06410293"[Title/Abstract] OR "PF 06410293"[Title/Abstract] OR "PF06410293"[Title/Abstract] OR "PF-06438179"[Title/Abstract] OR "PF 06438179"[Title/Abstract] OR "PF06438179"[Title/Abstract] OR "GP 1111"[Title/Abstract] OR "GP-1111"[Title/Abstract] OR "GP1111"[Title/Abstract] OR "Remtolumab"[Title/Abstract] OR "ABT-122"[Title/Abstract] OR "ABT 122"[Title/Abstract] OR "ABT122"[Title/Abstract] OR "1791410-27-9"[Title/Abstract] OR "AN0128"[Title/Abstract] OR "AN 0128"[Title/Abstract] OR "AN-0128"[Title/Abstract] OR "CRM-0005"[Title/Abstract] OR "CRM 0005"[Title/Abstract] OR "CRM0005"[Title/Abstract] OR "872044-70-7"[Title/Abstract] OR "Apeptico"[Title/Abstract] OR "AP-301"[Title/Abstract] OR "AP 301"[Title/Abstract] OR "AP301"[Title/Abstract] OR "Baicalein"[Title/Abstract] OR "Noroxylin"[Title/Abstract] OR "Baikalein"[Title/Abstract] OR "Baicelein"[Title/Abstract] OR "Baikelein"[Title/Abstract] OR "NSC661431"[Title/Abstract] OR "NSC 661431"[Title/Abstract] OR "NSC-661431"[Title/Abstract] OR "Sho-saiko-to"[Title/Abstract] OR "491-67-8"[Title/Abstract] OR "C15H10O5"[Title/Abstract] OR "Delenex"[Title/Abstract] OR "DLX-105"[Title/Abstract] OR "DLX 105"[Title/Abstract] OR "DLX105"[Title/Abstract] OR "ESBA-105"[Title/Abstract] OR "ESBA 105"[Title/Abstract] OR "ESBA105"[Title/Abstract] OR "1142149-71-0"[Title/Abstract] OR "Ortataxel"[Title/Abstract] OR "8H61Y4E29N"[Title/Abstract] OR "IDN-5109"[Title/Abstract] OR "IDN 5109"[Title/Abstract] OR "IDN5109"[Title/Abstract] OR "Bay-59*"[Title/Abstract] OR "Bay 59*"[Title/Abstract] OR "Bay59*"[Title/Abstract] OR "Hexanoic acid"[Title/Abstract] OR "Genz 29155"[Title/Abstract] OR "Genz-29155"[Title/Abstract] OR "Genz29155"[Title/Abstract] OR "SB-T-101131"[Title/Abstract] OR "SB T 101131"[Title/Abstract] OR "SBT101131"[Title/Abstract] OR "186348-23-2"[Title/Abstract] OR "C44H57NO17"[Title/Abstract] OR "Pegsunercept"[Title/Abstract] OR "PEGylated soluble tumor necrosis factor receptor I"[Title/Abstract] OR "PEG sTNFRI"[Title/Abstract] OR "STNF-RI"[Title/Abstract] OR "STNF RI"[Title/Abstract] OR "STNFRI"[Title/Abstract] OR "STNF-R1"[Title/Abstract] OR "STNF R1"[Title/Abstract] OR "STNFR1"[Title/Abstract] OR "330988-75-5"[Title/Abstract] OR "C502H758N154O165S16"[Title/Abstract] OR "Allotrap"[Title/Abstract] OR "Delmitide"[Title/Abstract] OR "Peptide Bc-1nl"[Title/Abstract] OR "RDP 58"[Title/Abstract] OR "RDP-58"[Title/Abstract] OR "RDP58"[Title/Abstract] OR "RSP 58"[Title/Abstract] OR "RSP-58"[Title/Abstract] OR "RSP58"[Title/Abstract] OR "287096-87-1"[Title/Abstract] OR "C59H105N17O11"[Title/Abstract] OR "Kinoid"[Title/Abstract] OR "Neovacs"[Title/Abstract] OR "Debio-0512"[Title/Abstract] OR "Debio 0512"[Title/Abstract] OR "Debio0512"[Title/Abstract] OR "COVA322"[Title/Abstract] OR "COVA-322"[Title/Abstract] OR "COVA 322"[Title/Abstract] OR "GTPL10469"[Title/Abstract] OR "GTPL 10469"[Title/Abstract] OR "GTPL-10469"[Title/Abstract] OR "Placulumab"[Title/Abstract] OR "PN-0621"[Title/Abstract] OR "PN 0621"[Title/Abstract] OR "PN0621"[Title/Abstract] OR "CEP-37247"[Title/Abstract] OR "CEP 37247"[Title/Abstract] OR "CEP37247"[Title/Abstract] OR "ART621"[Title/Abstract] OR "ART 621"[Title/Abstract] OR "ART-621"[Title/Abstract] OR "ABBV-257"[Title/Abstract] OR "ABBV 257"[Title/Abstract] OR "ABBV257"[Title/Abstract] OR "MA59O0RPES"[Title/Abstract] OR "AST-005"[Title/Abstract] OR "AST 005"[Title/Abstract] OR "AST005"[Title/Abstract] OR "AVX-470"[Title/Abstract] OR "AVX 470"[Title/Abstract] OR "AVX470"[Title/Abstract] OR "Aurimune"[Title/Abstract] OR "CYT-609"[Title/Abstract] OR "CYT 609"[Title/Abstract] OR "CYT609"[Title/Abstract] OR "877170-68-8"[Title/Abstract] OR "INB03"[Title/Abstract] OR "INB 03"[Title/Abstract] OR "INB-03"[Title/Abstract] OR "PF-05230905"[Title/Abstract] OR "PF 05230905"[Title/Abstract] OR "PF05230905"[Title/Abstract] OR "ATN-192"[Title/Abstract] OR "ATN 192"[Title/Abstract] OR "ATN192"[Title/Abstract] OR "PMI-005"[Title/Abstract] OR "PMI 005"[Title/Abstract] OR "PMI005"[Title/Abstract] OR "ABX-0401"[Title/Abstract] OR "ABX 0401"[Title/Abstract] OR "ABX0401"[Title/Abstract] OR "ALX-0071"[Title/Abstract] OR "ALX 0071"[Title/Abstract] OR "ALX0071"[Title/Abstract] OR "Celastrol"[Title/Abstract] OR "Tripterin"[Title/Abstract] OR "Celastrus scandens"[Title/Abstract] OR "34157-83-0"[Title/Abstract] OR "C29H38O4"[Title/Abstract] OR "CDP571"[Title/Abstract] OR "CDP 571"[Title/Abstract] OR "CDP-571"[Title/Abstract] OR "Afelimomab"[Title/Abstract] OR "Segard"[Title/Abstract] OR "MAK-195F"[Title/Abstract] OR "MAK 195F"[Title/Abstract] OR "MAK195F"[Title/Abstract] OR "LU-54107"[Title/Abstract] OR "LU 54107"[Title/Abstract] OR "LU54107"[Title/Abstract] OR "156227-98-4"[Title/Abstract] OR "Camobucol"[Title/Abstract] OR "ylgucpd100bdh"[Title/Abstract] OR "V-protectant"[Title/Abstract] OR "AGIX-4207"[Title/Abstract] OR "AGIX 4207"[Title/Abstract] OR "AGIX4207"[Title/Abstract] OR "216167-92-9"[Title/Abstract] OR "C33H50O4S2"[Title/Abstract] OR "CRX-191"[Title/Abstract] OR "CRX 191"[Title/Abstract] OR "CRX191"[Title/Abstract] OR "AME-527"[Title/Abstract] OR "AME 527"[Title/Abstract] OR "AME527"[Title/Abstract] OR "1001159-86-9"[Title/Abstract] OR "CYT-007-TNFQb"[Title/Abstract] OR "CYT 007 TNFQb"[Title/Abstract] OR "CYT007TNFQb"[Title/Abstract] OR "ALS-00T2-0501"[Title/Abstract] OR "ALS 00T2 0501"[Title/Abstract] OR "ALS00T20501"[Title/Abstract] OR "FR-133605"[Title/Abstract] OR "FR 133605"[Title/Abstract] OR "FR133605"[Title/Abstract] OR "MDL-201112"[Title/Abstract] OR "MDL 201112"[Title/Abstract] OR "MDL201112"[Title/Abstract] OR "MDL-201449"[Title/Abstract] OR "MDL 201449"[Title/Abstract] OR "MDL201449"[Title/Abstract] OR "MDL-201449A"[Title/Abstract] OR "MDL 201449A"[Title/Abstract] OR "MDL201449A"[Title/Abstract] OR "142130-73-2"[Title/Abstract] OR "C10H13N5O"[Title/Abstract] OR "Carbocyclic nucleoside"[Title/Abstract]) AND ("Alopecia cicatrisata*"[Title/Abstract] OR "Cicatricial alopeci*"[Title/Abstract] OR "Scarring alopeci*"[Title/Abstract] OR "Irreversible alopeci*"[Title/Abstract] OR "Permanent alopeci*"[Title/Abstract] OR "Cicatricial bald*"[Title/Abstract] OR "Scarring bald*"[Title/Abstract] OR "Irreversible bald*"[Title/Abstract] OR "Permanent bald*"[Title/Abstract] OR "Cicatricial hair los*"[Title/Abstract] OR "Scarring hair los*"[Title/Abstract] OR "Irreversible hair los*"[Title/Abstract] OR "Permanent hair los*"[Title/Abstract] OR "Pseudopelad*"[Title/Abstract] OR "Brocq"[Title/Abstract] OR "Scarring lichen plan*"[Title/Abstract] OR "Scalp lichen plan*"[Title/Abstract] OR "Lichen planopilar*"[Title/Abstract] OR "Coup de sabre*"[Title/Abstract] OR "Discoid lupus*"[Title/Abstract] OR "Folliculitis decalvans"[Title/Abstract] OR "Erosive pustular dermatosis"[Title/Abstract] OR "Chronic atrophic dermatosis"[Title/Abstract] OR "Frontal fibrosing alopecia"[Title/Abstract] OR "Central centrifugal alopecia"[Title/Abstract] OR "Central centrifugal cicatricial alopecia"[Title/Abstract] OR "Tufted folliculitis"[Title/Abstract] OR "Dissecting cellulitis"[Title/Abstract] OR "Perifolliculitis capitis"[Title/Abstract] OR "Folliculitis keloidalis"[Title/Abstract] OR "Acne cheloidalis nuch*"[Title/Abstract] OR "Acne keloidalis"[Title/Abstract] OR "Papular atrichia"[Title/Abstract] OR "Atrichia with papular lesion*"[Title/Abstract]) | 191 |
| Scopus | (TITLE-ABS-KEY("*JAK inhibit*") OR TITLE-ABS-KEY("*Jakinib*") OR TITLE-ABS-KEY("*Janus kinase inhibit*") OR TITLE-ABS-KEY("*Tyrosine kinase inhibit*") OR TITLE-ABS-KEY("*JAK-STAT inhibit*") OR TITLE-ABS-KEY("*JAKi*") OR TITLE-ABS-KEY("*JAK i*") OR TITLE-ABS-KEY("*JAK-i*") OR TITLE-ABS-KEY("*JAK1 inhibit*") OR TITLE-ABS-KEY("*JAK2 inhibit*") OR TITLE-ABS-KEY("*JAK3 inhibit*") OR TITLE-ABS-KEY("*TYK2 inhibit*") OR TITLE-ABS-KEY("*Janus kinase 1 inhibit*") OR TITLE-ABS-KEY("*Janus kinase 2 inhibit*") OR TITLE-ABS-KEY("*Janus kinase 3 inhibit*") OR TITLE-ABS-KEY("*Tyrosine kinase 2 inhibit*") OR TITLE-ABS-KEY("Ruxolitinib") OR TITLE-ABS-KEY("Jakafi") OR TITLE-ABS-KEY("Jakavi") OR TITLE-ABS-KEY("Opzelura") OR TITLE-ABS-KEY("INCB018424") OR TITLE-ABS-KEY("INCB-018424") OR TITLE-ABS-KEY("INCB 018424") OR TITLE-ABS-KEY("INCB18424") OR TITLE-ABS-KEY("INCB-18424") OR TITLE-ABS-KEY("INCB 18424") OR TITLE-ABS-KEY("INCA24") OR TITLE-ABS-KEY("INC-A24") OR TITLE-ABS-KEY("INC A24") OR TITLE-ABS-KEY("INC424") OR TITLE-ABS-KEY("INC-424") OR TITLE-ABS-KEY("INC 424") OR TITLE-ABS-KEY("941678-49-5") OR TITLE-ABS-KEY("C17H18N6") OR TITLE-ABS-KEY("C17H21N6O4P") OR TITLE-ABS-KEY("Tofacitinib") OR TITLE-ABS-KEY("Tasocitinib") OR TITLE-ABS-KEY("Xeljanz") OR TITLE-ABS-KEY("Jaquinus") OR TITLE-ABS-KEY("CP-690550") OR TITLE-ABS-KEY("CP 690550") OR TITLE-ABS-KEY("CP690550") OR TITLE-ABS-KEY("CP-690,550") OR TITLE-ABS-KEY("CP 690,550") OR TITLE-ABS-KEY("CP690,550") OR TITLE-ABS-KEY("477600-75-2") OR TITLE-ABS-KEY("C16H20N6O") OR TITLE-ABS-KEY("Oclacitinib") OR TITLE-ABS-KEY("Apoquel") OR TITLE-ABS-KEY("PF 03394197") OR TITLE-ABS-KEY("PF-03394197") OR TITLE-ABS-KEY("PF03394197") OR TITLE-ABS-KEY("1208319-26-9") OR TITLE-ABS-KEY("C15H23N5O2S") OR TITLE-ABS-KEY("Baricitinib") OR TITLE-ABS-KEY("Olumiant") OR TITLE-ABS-KEY("INCB 028050") OR TITLE-ABS-KEY("INCB-028050") OR TITLE-ABS-KEY("INCB028050") OR TITLE-ABS-KEY("INCB 28050") OR TITLE-ABS-KEY("INCB-28050") OR TITLE-ABS-KEY("INCB28050") OR TITLE-ABS-KEY("LY 3009104") OR TITLE-ABS-KEY("LY-3009104") OR TITLE-ABS-KEY("LY3009104") OR TITLE-ABS-KEY("1187594-09-7") OR TITLE-ABS-KEY("C16H17N7O2S") OR TITLE-ABS-KEY("Peficitinib") OR TITLE-ABS-KEY("Smyraf") OR TITLE-ABS-KEY("ASP 015K") OR TITLE-ABS-KEY("ASP-015K") OR TITLE-ABS-KEY("ASP015K") OR TITLE-ABS-KEY("JNJ 54781532") OR TITLE-ABS-KEY("JNJ-54781532") OR TITLE-ABS-KEY("JNJ54781532") OR TITLE-ABS-KEY("944118-01-8") OR TITLE-ABS-KEY("C18H22N4O2") OR TITLE-ABS-KEY("Upadacitinib") OR TITLE-ABS-KEY("Rinvoq") OR TITLE-ABS-KEY("ABT 494") OR TITLE-ABS-KEY("ABT-494") OR TITLE-ABS-KEY("ABT494") OR TITLE-ABS-KEY("1310726-60-3") OR TITLE-ABS-KEY("C17H19F3N6O") OR TITLE-ABS-KEY("Fedratinib") OR TITLE-ABS-KEY("Inrebic") OR TITLE-ABS-KEY("SAR 302503") OR TITLE-ABS-KEY("SAR-302503") OR TITLE-ABS-KEY("SAR302503") OR TITLE-ABS-KEY("TG 101348") OR TITLE-ABS-KEY("TG-101348") OR TITLE-ABS-KEY("TG101348") OR TITLE-ABS-KEY("936091-26-8") OR TITLE-ABS-KEY("C27H36N6O3S") OR TITLE-ABS-KEY("Delgocitinib") OR TITLE-ABS-KEY("Corectim") OR TITLE-ABS-KEY("JTE 052") OR TITLE-ABS-KEY("JTE-052") OR TITLE-ABS-KEY("JTE052") OR TITLE-ABS-KEY("JTE 052A") OR TITLE-ABS-KEY("JTE-052A") OR TITLE-ABS-KEY("JTE052A") OR TITLE-ABS-KEY("LEO 124249") OR TITLE-ABS-KEY("LEO-124249") OR TITLE-ABS-KEY("LEO124249") OR TITLE-ABS-KEY("LEO 124249A") OR TITLE-ABS-KEY("LEO-124249A") OR TITLE-ABS-KEY("LEO124249A") OR TITLE-ABS-KEY("1263774-59-9") OR TITLE-ABS-KEY("C16H18N6O") OR TITLE-ABS-KEY("Filgotinib") OR TITLE-ABS-KEY("Jyseleca") OR TITLE-ABS-KEY("GS 6034") OR TITLE-ABS-KEY("GS-6034") OR TITLE-ABS-KEY("GS6034") OR TITLE-ABS-KEY("GLPG 0634") OR TITLE-ABS-KEY("GLPG-0634") OR TITLE-ABS-KEY("GLPG0634") OR TITLE-ABS-KEY("G 146034") OR TITLE-ABS-KEY("G-146034") OR TITLE-ABS-KEY("G146034") OR TITLE-ABS-KEY("1206101-20-3") OR TITLE-ABS-KEY("1206161-97-8") OR TITLE-ABS-KEY("C21H23N5O3S") OR TITLE-ABS-KEY("Abrocitinib") OR TITLE-ABS-KEY("Cibinqo") OR TITLE-ABS-KEY("PF 04965842") OR TITLE-ABS-KEY("PF-04965842") OR TITLE-ABS-KEY("PF04965842") OR TITLE-ABS-KEY("1622902-68-4") OR TITLE-ABS-KEY("C14H21N5O2S") OR TITLE-ABS-KEY("Pacritinib") OR TITLE-ABS-KEY("Vonjo") OR TITLE-ABS-KEY("SB 1518") OR TITLE-ABS-KEY("SB-1518") OR TITLE-ABS-KEY("SB1518") OR TITLE-ABS-KEY("937272-79-2") OR TITLE-ABS-KEY("C28H32N4O3") OR TITLE-ABS-KEY("Deucravacitinib") OR TITLE-ABS-KEY("Sotyktu") OR TITLE-ABS-KEY("BMS 986165") OR TITLE-ABS-KEY("BMS-986165") OR TITLE-ABS-KEY("BMS986165") OR TITLE-ABS-KEY("Tyk2-IN-4") OR TITLE-ABS-KEY("Tyk2 IN 4") OR TITLE-ABS-KEY("Tyk2IN4") OR TITLE-ABS-KEY("1609392-27-9") OR TITLE-ABS-KEY("C20H22N8O3") OR TITLE-ABS-KEY("Cerdulatinib") OR TITLE-ABS-KEY("PRT 2070") OR TITLE-ABS-KEY("PRT-2070") OR TITLE-ABS-KEY("PRT2070") OR TITLE-ABS-KEY("PRT 062070") OR TITLE-ABS-KEY("PRT-062070") OR TITLE-ABS-KEY("PRT062070") OR TITLE-ABS-KEY("DMVT 502") OR TITLE-ABS-KEY("DMVT-502") OR TITLE-ABS-KEY("DMVT502") OR TITLE-ABS-KEY("RVT 502") OR TITLE-ABS-KEY("RVT-502") OR TITLE-ABS-KEY("RVT502") OR TITLE-ABS-KEY("1198300-79-6") OR TITLE-ABS-KEY("C20H27N7O3S") OR TITLE-ABS-KEY("Gandotinib") OR TITLE-ABS-KEY("LY 2784544") OR TITLE-ABS-KEY("LY-2784544") OR TITLE-ABS-KEY("LY2784544") OR TITLE-ABS-KEY("1229236-86-5") OR TITLE-ABS-KEY("C23H25ClFN7O") OR TITLE-ABS-KEY("Lestaurtinib") OR TITLE-ABS-KEY("CEP 701") OR TITLE-ABS-KEY("CEP-701") OR TITLE-ABS-KEY("CEP701") OR TITLE-ABS-KEY("KT 5555") OR TITLE-ABS-KEY("KT-5555") OR TITLE-ABS-KEY("KT5555") OR TITLE-ABS-KEY("SPM 924") OR TITLE-ABS-KEY("SPM-924") OR TITLE-ABS-KEY("SPM924") OR TITLE-ABS-KEY("KT 555") OR TITLE-ABS-KEY("KT-555") OR TITLE-ABS-KEY("KT555") OR TITLE-ABS-KEY("111358-88-4") OR TITLE-ABS-KEY("C26H21N3O4") OR TITLE-ABS-KEY("Momelotinib") OR TITLE-ABS-KEY("CYT 387") OR TITLE-ABS-KEY("CYT-387") OR TITLE-ABS-KEY("CYT387") OR TITLE-ABS-KEY("CYT 11387") OR TITLE-ABS-KEY("CYT-11387") OR TITLE-ABS-KEY("CYT11387") OR TITLE-ABS-KEY("GS-0387") OR TITLE-ABS-KEY("GS 0387") OR TITLE-ABS-KEY("GS0387") OR TITLE-ABS-KEY("GS-387") OR TITLE-ABS-KEY("GS 387") OR TITLE-ABS-KEY("GS387") OR TITLE-ABS-KEY("LM 1149") OR TITLE-ABS-KEY("LM-1149") OR TITLE-ABS-KEY("LM1149") OR TITLE-ABS-KEY("1056634-68-4") OR TITLE-ABS-KEY("Cucurbitacin") OR TITLE-ABS-KEY("Elatericin") OR TITLE-ABS-KEY("JSI 124") OR TITLE-ABS-KEY("JSI-124") OR TITLE-ABS-KEY("JSI124") OR TITLE-ABS-KEY("NSC 521777") OR TITLE-ABS-KEY("NSC-521777") OR TITLE-ABS-KEY("NSC521777") OR TITLE-ABS-KEY("NSC 112167") OR TITLE-ABS-KEY("NSC-112167") OR TITLE-ABS-KEY("NSC112167") OR TITLE-ABS-KEY("2222-07-3") OR TITLE-ABS-KEY("C30H42O7") OR TITLE-ABS-KEY("CHZ 868") OR TITLE-ABS-KEY("CHZ-868") OR TITLE-ABS-KEY("CHZ868") OR TITLE-ABS-KEY("C22H19F2N5O2") OR TITLE-ABS-KEY("Tumor Necrosis Factor inhib*") OR TITLE-ABS-KEY("Tumor Necrosis Factor-a inhib*") OR TITLE-ABS-KEY("Tumor Necrosis Factor-α inhib*") OR TITLE-ABS-KEY("Tumor Necrosis Factor-alpha inhib*") OR TITLE-ABS-KEY("Tumor Necrosis Factor a inhib*") OR TITLE-ABS-KEY("Tumor Necrosis Factor α inhib*") OR TITLE-ABS-KEY("Tumor Necrosis Factor alpha inhib*") OR TITLE-ABS-KEY("Tumor Necrosis Factor antag*") OR TITLE-ABS-KEY("Tumor Necrosis Factor-a antag*") OR TITLE-ABS-KEY("Tumor Necrosis Factor-α antag*") OR TITLE-ABS-KEY("Tumor Necrosis Factor-alpha antag*") OR TITLE-ABS-KEY("Tumor Necrosis Factor a antag*") OR TITLE-ABS-KEY("Tumor Necrosis Factor α antag*") OR TITLE-ABS-KEY("Tumor Necrosis Factor alpha antag*") OR TITLE-ABS-KEY("Tumor Necrosis Factor block*") OR TITLE-ABS-KEY("Tumor Necrosis Factor-a block*") OR TITLE-ABS-KEY("Tumor Necrosis Factor-α block*") OR TITLE-ABS-KEY("Tumor Necrosis Factor-alpha block*") OR TITLE-ABS-KEY("Tumor Necrosis Factor a block*") OR TITLE-ABS-KEY("Tumor Necrosis Factor α block*") OR TITLE-ABS-KEY("Tumor Necrosis Factor alpha block*") OR TITLE-ABS-KEY("Tumour Necrosis Factor inhib*") OR TITLE-ABS-KEY("Tumour Necrosis Factor-a inhib*") OR TITLE-ABS-KEY("Tumour Necrosis Factor-α inhib*") OR TITLE-ABS-KEY("Tumour Necrosis Factor-alpha inhib*") OR TITLE-ABS-KEY("Tumour Necrosis Factor a inhib*") OR TITLE-ABS-KEY("Tumour Necrosis Factor α inhib*") OR TITLE-ABS-KEY("Tumour Necrosis Factor alpha inhib*") OR TITLE-ABS-KEY("Tumour Necrosis Factor antag*") OR TITLE-ABS-KEY("Tumour Necrosis Factor-a antag*") OR TITLE-ABS-KEY("Tumour Necrosis Factor-α antag*") OR TITLE-ABS-KEY("Tumour Necrosis Factor-alpha antag*") OR TITLE-ABS-KEY("Tumour Necrosis Factor a antag*") OR TITLE-ABS-KEY("Tumour Necrosis Factor α antag*") OR TITLE-ABS-KEY("Tumour Necrosis Factor alpha antag*") OR TITLE-ABS-KEY("Tumour Necrosis Factor block*") OR TITLE-ABS-KEY("Tumour Necrosis Factor-a block*") OR TITLE-ABS-KEY("Tumour Necrosis Factor-α block*") OR TITLE-ABS-KEY("Tumour Necrosis Factor-alpha block*") OR TITLE-ABS-KEY("Tumour Necrosis Factor a block*") OR TITLE-ABS-KEY("Tumour Necrosis Factor α block*") OR TITLE-ABS-KEY("Tumour Necrosis Factor alpha block*") OR TITLE-ABS-KEY("TNF inhib*") OR TITLE-ABS-KEY("TNF-a inhib*") OR TITLE-ABS-KEY("TNF-α inhib*") OR TITLE-ABS-KEY("TNF-alpha inhib*") OR TITLE-ABS-KEY("TNF a inhib*") OR TITLE-ABS-KEY("TNF α inhib*") OR TITLE-ABS-KEY("TNF alpha inhib*") OR TITLE-ABS-KEY("TNF antag*") OR TITLE-ABS-KEY("TNF-a antag*") OR TITLE-ABS-KEY("TNF-α antag*") OR TITLE-ABS-KEY("TNF-alpha antag*") OR TITLE-ABS-KEY("TNF a antag*") OR TITLE-ABS-KEY("TNF α antag*") OR TITLE-ABS-KEY("TNF alpha antag*") OR TITLE-ABS-KEY("TNF block*") OR TITLE-ABS-KEY("TNF-a block*") OR TITLE-ABS-KEY("TNF-α block*") OR TITLE-ABS-KEY("TNF-alpha block*") OR TITLE-ABS-KEY("TNF a block*") OR TITLE-ABS-KEY("TNF α block*") OR TITLE-ABS-KEY("TNF alpha block*") OR TITLE-ABS-KEY("Anti-Tumour Necrosis Factor*") OR TITLE-ABS-KEY("Anti-TNF*") OR TITLE-ABS-KEY("Cachectin inhib*") OR TITLE-ABS-KEY("Cachectin antag*") OR TITLE-ABS-KEY("Cachectin block*") OR TITLE-ABS-KEY("Infliximab") OR TITLE-ABS-KEY("Remicade") OR TITLE-ABS-KEY("Inflectra") OR TITLE-ABS-KEY("Avsola") OR TITLE-ABS-KEY("Ixifi") OR TITLE-ABS-KEY("Renflexis") OR TITLE-ABS-KEY("Mab cA2") OR TITLE-ABS-KEY("Monoclonal antibody cA2") OR TITLE-ABS-KEY("TA-650") OR TITLE-ABS-KEY("TA 650") OR TITLE-ABS-KEY("TA650") OR TITLE-ABS-KEY("170277-31-3") OR TITLE-ABS-KEY("Adalimumab") OR TITLE-ABS-KEY("Humira") OR TITLE-ABS-KEY("Abrilada") OR TITLE-ABS-KEY("Amgevita") OR TITLE-ABS-KEY("Amjevita") OR TITLE-ABS-KEY("Amsparity") OR TITLE-ABS-KEY("Ardalicip") OR TITLE-ABS-KEY("Cadalimab") OR TITLE-ABS-KEY("Ciptunec") OR TITLE-ABS-KEY("Cyltezo") OR TITLE-ABS-KEY("Exemptia") OR TITLE-ABS-KEY("Hadlima") OR TITLE-ABS-KEY("Halimatoz") OR TITLE-ABS-KEY("Hefiya") OR TITLE-ABS-KEY("Hukyndra") OR TITLE-ABS-KEY("Hulio") OR TITLE-ABS-KEY("Hyrimoz") OR TITLE-ABS-KEY("Idacio") OR TITLE-ABS-KEY("Imraldi") OR TITLE-ABS-KEY("Kromeya") OR TITLE-ABS-KEY("Libmyris") OR TITLE-ABS-KEY("Mabura") OR TITLE-ABS-KEY("Simlandi") OR TITLE-ABS-KEY("Solymbic") OR TITLE-ABS-KEY("Trudexa") OR TITLE-ABS-KEY("Yuflyma") OR TITLE-ABS-KEY("Yusimry") OR TITLE-ABS-KEY("D2E7") OR TITLE-ABS-KEY("331731-18-1") OR TITLE-ABS-KEY("C6428H9912N1694O1987S46") OR TITLE-ABS-KEY("Etanercept") OR TITLE-ABS-KEY("Enbrel") OR TITLE-ABS-KEY("Erelzi") OR TITLE-ABS-KEY("Eticovo") OR TITLE-ABS-KEY("Benepali") OR TITLE-ABS-KEY("TNFR-Fc") OR TITLE-ABS-KEY("TNFR Fc") OR TITLE-ABS-KEY("TNT Receptor Fusion Protein") OR TITLE-ABS-KEY("TNF Receptor Type II-IgG Fusion Protein") OR TITLE-ABS-KEY("TNF Receptor Type II IgG Fusion Protein") OR TITLE-ABS-KEY("TNR 001") OR TITLE-ABS-KEY("TNR-001") OR TITLE-ABS-KEY("TNR001") OR TITLE-ABS-KEY("185243-69-0") OR TITLE-ABS-KEY("C2224H3475N621O698S36") OR TITLE-ABS-KEY("Golimumab") OR TITLE-ABS-KEY("Simponi") OR TITLE-ABS-KEY("CNTO-148") OR TITLE-ABS-KEY("CNTO 148") OR TITLE-ABS-KEY("CNTO148") OR TITLE-ABS-KEY("476181-74-5") OR TITLE-ABS-KEY("C6530H10068N1752O2026S44") OR TITLE-ABS-KEY("Certolizumab") OR TITLE-ABS-KEY("Cimzia") OR TITLE-ABS-KEY("CDP 870") OR TITLE-ABS-KEY("CDP-870") OR TITLE-ABS-KEY("CDP870") OR TITLE-ABS-KEY("428863-50-7") OR TITLE-ABS-KEY("C2115H3252N556O673S16") OR TITLE-ABS-KEY("Thalidomide") OR TITLE-ABS-KEY("Thalomid*") OR TITLE-ABS-KEY("Algosediv") OR TITLE-ABS-KEY("Asmadion") OR TITLE-ABS-KEY("Asmaval") OR TITLE-ABS-KEY("Bonbrain") OR TITLE-ABS-KEY("Calmore") OR TITLE-ABS-KEY("Calmorex") OR TITLE-ABS-KEY("Contergan") OR TITLE-ABS-KEY("Corronarobetin") OR TITLE-ABS-KEY("Distaval") OR TITLE-ABS-KEY("Ectiluran") OR TITLE-ABS-KEY("Enterosediv") OR TITLE-ABS-KEY("Gastrinide") OR TITLE-ABS-KEY("Glupan") OR TITLE-ABS-KEY("Glutanon") OR TITLE-ABS-KEY("Grippex") OR TITLE-ABS-KEY("Hippuzon") OR TITLE-ABS-KEY("Imidene") OR TITLE-ABS-KEY("Isomin") OR TITLE-ABS-KEY("Kevadon") OR TITLE-ABS-KEY("Neosedyn") OR TITLE-ABS-KEY("Nerosedyn") OR TITLE-ABS-KEY("Neufatin") OR TITLE-ABS-KEY("Neurodyn") OR TITLE-ABS-KEY("Neurosedin") OR TITLE-ABS-KEY("Neurosedym") OR TITLE-ABS-KEY("Nevrodyn") OR TITLE-ABS-KEY("Nibrol") OR TITLE-ABS-KEY("Noctosediv") OR TITLE-ABS-KEY("Noxodyn") OR TITLE-ABS-KEY("Pangul") OR TITLE-ABS-KEY("Pantosediv") OR TITLE-ABS-KEY("Phthalimidoglut") OR TITLE-ABS-KEY("Phthaloylglut") OR TITLE-ABS-KEY("Phthalylglut") OR TITLE-ABS-KEY("Polygripan") OR TITLE-ABS-KEY("Profarmil") OR TITLE-ABS-KEY("Psycholiquid") OR TITLE-ABS-KEY("Psychotablet") OR TITLE-ABS-KEY("Quetimid") OR TITLE-ABS-KEY("Quietoplex") OR TITLE-ABS-KEY("Sandormin") OR TITLE-ABS-KEY("Sedimide") OR TITLE-ABS-KEY("Sedisperil") OR TITLE-ABS-KEY("Sedoval") OR TITLE-ABS-KEY("Shinnibrol") OR TITLE-ABS-KEY("Sleepan") OR TITLE-ABS-KEY("Slipro") OR TITLE-ABS-KEY("Softenil") OR TITLE-ABS-KEY("Softenon") OR TITLE-ABS-KEY("Talargan") OR TITLE-ABS-KEY("Talimol") OR TITLE-ABS-KEY("Talismol") OR TITLE-ABS-KEY("Telagan") OR TITLE-ABS-KEY("Telargean") OR TITLE-ABS-KEY("Tensival") OR TITLE-ABS-KEY("Thalin") OR TITLE-ABS-KEY("Thalinette") OR TITLE-ABS-KEY("Theophilcholine") OR TITLE-ABS-KEY("Valgis") OR TITLE-ABS-KEY("Valgraine") OR TITLE-ABS-KEY("50-35-1") OR TITLE-ABS-KEY("C13H10N2O4") OR TITLE-ABS-KEY("Lenalidomide") OR TITLE-ABS-KEY("Revlimid") OR TITLE-ABS-KEY("Linamide") OR TITLE-ABS-KEY("Revamid") OR TITLE-ABS-KEY("Revimid") OR TITLE-ABS-KEY("CC 5013") OR TITLE-ABS-KEY("CC-5013") OR TITLE-ABS-KEY("CC5013") OR TITLE-ABS-KEY("CDC 501") OR TITLE-ABS-KEY("CDC-501") OR TITLE-ABS-KEY("CDC501") OR TITLE-ABS-KEY("IMiD3") OR TITLE-ABS-KEY("IMID-5013") OR TITLE-ABS-KEY("IMID 5013") OR TITLE-ABS-KEY("IMID5013") OR TITLE-ABS-KEY("ENMD-0997") OR TITLE-ABS-KEY("ENMD 0997") OR TITLE-ABS-KEY("ENMD0997") OR TITLE-ABS-KEY("191732-72-6") OR TITLE-ABS-KEY("C13H13N3O3") OR TITLE-ABS-KEY("Nafamostat") OR TITLE-ABS-KEY("Simponi") OR TITLE-ABS-KEY("Ronastat") OR TITLE-ABS-KEY("CKD-314") OR TITLE-ABS-KEY("CKD 314") OR TITLE-ABS-KEY("CKD314") OR TITLE-ABS-KEY("FUT-175") OR TITLE-ABS-KEY("FUT 175") OR TITLE-ABS-KEY("FUT175") OR TITLE-ABS-KEY("81525-10-2") OR TITLE-ABS-KEY("C19H17N5O2") OR TITLE-ABS-KEY("Pentoxifylline") OR TITLE-ABS-KEY("Oxpentifylline") OR TITLE-ABS-KEY("Trental") OR TITLE-ABS-KEY("Torental") OR TITLE-ABS-KEY("Pentoxil") OR TITLE-ABS-KEY("Dimethyloxohexylxanthine") OR TITLE-ABS-KEY("Agapurin") OR TITLE-ABS-KEY("Azupentat") OR TITLE-ABS-KEY("Durapental") OR TITLE-ABS-KEY("Hemovas") OR TITLE-ABS-KEY("Ikomio") OR TITLE-ABS-KEY("Pentoxifil") OR TITLE-ABS-KEY("Ralofect") OR TITLE-ABS-KEY("Rentylin") OR TITLE-ABS-KEY("Vasofirin") OR TITLE-ABS-KEY("Vazofirin") OR TITLE-ABS-KEY("Pentoxin") OR TITLE-ABS-KEY("BL 191") OR TITLE-ABS-KEY("BL-191") OR TITLE-ABS-KEY("BL191") OR TITLE-ABS-KEY("6493-05-6") OR TITLE-ABS-KEY("C13H18N4O3") OR TITLE-ABS-KEY("ABP 501") OR TITLE-ABS-KEY("ABP-501") OR TITLE-ABS-KEY("ABP501") OR TITLE-ABS-KEY("Ajulemic") OR TITLE-ABS-KEY("Lenabasum") OR TITLE-ABS-KEY("Resunab") OR TITLE-ABS-KEY("Cervelo") OR TITLE-ABS-KEY("Anabasum") OR TITLE-ABS-KEY("IP-751") OR TITLE-ABS-KEY("IP 751") OR TITLE-ABS-KEY("IP751") OR TITLE-ABS-KEY("CPL-7075") OR TITLE-ABS-KEY("CPL 7075") OR TITLE-ABS-KEY("CPL7075") OR TITLE-ABS-KEY("CT-3") OR TITLE-ABS-KEY("CT 3") OR TITLE-ABS-KEY("CT3") OR TITLE-ABS-KEY("137945-48-3") OR TITLE-ABS-KEY("Golnerminogene") OR TITLE-ABS-KEY("TNFerade") OR TITLE-ABS-KEY("957472-14-9") OR TITLE-ABS-KEY("Ad5FGF-4") OR TITLE-ABS-KEY("Ad5FGF 4") OR TITLE-ABS-KEY("Ad5FGF4") OR TITLE-ABS-KEY("PF-06410293") OR TITLE-ABS-KEY("PF 06410293") OR TITLE-ABS-KEY("PF06410293") OR TITLE-ABS-KEY("PF-06438179") OR TITLE-ABS-KEY("PF 06438179") OR TITLE-ABS-KEY("PF06438179") OR TITLE-ABS-KEY("GP 1111") OR TITLE-ABS-KEY("GP-1111") OR TITLE-ABS-KEY("GP1111") OR TITLE-ABS-KEY("Remtolumab") OR TITLE-ABS-KEY("ABT-122") OR TITLE-ABS-KEY("ABT 122") OR TITLE-ABS-KEY("ABT122") OR TITLE-ABS-KEY("1791410-27-9") OR TITLE-ABS-KEY("AN0128") OR TITLE-ABS-KEY("AN 0128") OR TITLE-ABS-KEY("AN-0128") OR TITLE-ABS-KEY("CRM-0005") OR TITLE-ABS-KEY("CRM 0005") OR TITLE-ABS-KEY("CRM0005") OR TITLE-ABS-KEY("872044-70-7") OR TITLE-ABS-KEY("Apeptico") OR TITLE-ABS-KEY("AP-301") OR TITLE-ABS-KEY("AP 301") OR TITLE-ABS-KEY("AP301") OR TITLE-ABS-KEY("Baicalein") OR TITLE-ABS-KEY("Noroxylin") OR TITLE-ABS-KEY("Baikalein") OR TITLE-ABS-KEY("Baicelein") OR TITLE-ABS-KEY("Baikelein") OR TITLE-ABS-KEY("NSC661431") OR TITLE-ABS-KEY("NSC 661431") OR TITLE-ABS-KEY("NSC-661431") OR TITLE-ABS-KEY("Sho-saiko-to") OR TITLE-ABS-KEY("491-67-8") OR TITLE-ABS-KEY("C15H10O5") OR TITLE-ABS-KEY("Delenex") OR TITLE-ABS-KEY("DLX-105") OR TITLE-ABS-KEY("DLX 105") OR TITLE-ABS-KEY("DLX105") OR TITLE-ABS-KEY("ESBA-105") OR TITLE-ABS-KEY("ESBA 105") OR TITLE-ABS-KEY("ESBA105") OR TITLE-ABS-KEY("1142149-71-0") OR TITLE-ABS-KEY("Ortataxel") OR TITLE-ABS-KEY("8H61Y4E29N") OR TITLE-ABS-KEY("IDN-5109") OR TITLE-ABS-KEY("IDN 5109") OR TITLE-ABS-KEY("IDN5109") OR TITLE-ABS-KEY("Bay-59*") OR TITLE-ABS-KEY("Bay 59*") OR TITLE-ABS-KEY("Bay59*") OR TITLE-ABS-KEY("Hexanoic acid") OR TITLE-ABS-KEY("Genz 29155") OR TITLE-ABS-KEY("Genz-29155") OR TITLE-ABS-KEY("Genz29155") OR TITLE-ABS-KEY("SB-T-101131") OR TITLE-ABS-KEY("SB T 101131") OR TITLE-ABS-KEY("SBT101131") OR TITLE-ABS-KEY("186348-23-2") OR TITLE-ABS-KEY("C44H57NO17") OR TITLE-ABS-KEY("Pegsunercept") OR TITLE-ABS-KEY("PEGylated soluble tumor necrosis factor receptor I") OR TITLE-ABS-KEY("PEG sTNFRI") OR TITLE-ABS-KEY("STNF-RI") OR TITLE-ABS-KEY("STNF RI") OR TITLE-ABS-KEY("STNFRI") OR TITLE-ABS-KEY("STNF-R1") OR TITLE-ABS-KEY("STNF R1") OR TITLE-ABS-KEY("STNFR1") OR TITLE-ABS-KEY("330988-75-5") OR TITLE-ABS-KEY("C502H758N154O165S16") OR TITLE-ABS-KEY("Allotrap") OR TITLE-ABS-KEY("Delmitide") OR TITLE-ABS-KEY("Peptide Bc-1nl") OR TITLE-ABS-KEY("RDP 58") OR TITLE-ABS-KEY("RDP-58") OR TITLE-ABS-KEY("RDP58") OR TITLE-ABS-KEY("RSP 58") OR TITLE-ABS-KEY("RSP-58") OR TITLE-ABS-KEY("RSP58") OR TITLE-ABS-KEY("287096-87-1") OR TITLE-ABS-KEY("C59H105N17O11") OR TITLE-ABS-KEY("Kinoid") OR TITLE-ABS-KEY("Neovacs") OR TITLE-ABS-KEY("Debio-0512") OR TITLE-ABS-KEY("Debio 0512") OR TITLE-ABS-KEY("Debio0512") OR TITLE-ABS-KEY("COVA322") OR TITLE-ABS-KEY("COVA-322") OR TITLE-ABS-KEY("COVA 322") OR TITLE-ABS-KEY("GTPL10469") OR TITLE-ABS-KEY("GTPL 10469") OR TITLE-ABS-KEY("GTPL-10469") OR TITLE-ABS-KEY("Placulumab") OR TITLE-ABS-KEY("PN-0621") OR TITLE-ABS-KEY("PN 0621") OR TITLE-ABS-KEY("PN0621") OR TITLE-ABS-KEY("CEP-37247") OR TITLE-ABS-KEY("CEP 37247") OR TITLE-ABS-KEY("CEP37247") OR TITLE-ABS-KEY("ART621") OR TITLE-ABS-KEY("ART 621") OR TITLE-ABS-KEY("ART-621") OR TITLE-ABS-KEY("ABBV-257") OR TITLE-ABS-KEY("ABBV 257") OR TITLE-ABS-KEY("ABBV257") OR TITLE-ABS-KEY("MA59O0RPES") OR TITLE-ABS-KEY("AST-005") OR TITLE-ABS-KEY("AST 005") OR TITLE-ABS-KEY("AST005") OR TITLE-ABS-KEY("AVX-470") OR TITLE-ABS-KEY("AVX 470") OR TITLE-ABS-KEY("AVX470") OR TITLE-ABS-KEY("Aurimune") OR TITLE-ABS-KEY("CYT-609") OR TITLE-ABS-KEY("CYT 609") OR TITLE-ABS-KEY("CYT609") OR TITLE-ABS-KEY("877170-68-8") OR TITLE-ABS-KEY("INB03") OR TITLE-ABS-KEY("INB 03") OR TITLE-ABS-KEY("INB-03") OR TITLE-ABS-KEY("PF-05230905") OR TITLE-ABS-KEY("PF 05230905") OR TITLE-ABS-KEY("PF05230905") OR TITLE-ABS-KEY("ATN-192") OR TITLE-ABS-KEY("ATN 192") OR TITLE-ABS-KEY("ATN192") OR TITLE-ABS-KEY("PMI-005") OR TITLE-ABS-KEY("PMI 005") OR TITLE-ABS-KEY("PMI005") OR TITLE-ABS-KEY("ABX-0401") OR TITLE-ABS-KEY("ABX 0401") OR TITLE-ABS-KEY("ABX0401") OR TITLE-ABS-KEY("ALX-0071") OR TITLE-ABS-KEY("ALX 0071") OR TITLE-ABS-KEY("ALX0071") OR TITLE-ABS-KEY("Celastrol") OR TITLE-ABS-KEY("Tripterin") OR TITLE-ABS-KEY("Celastrus scandens") OR TITLE-ABS-KEY("34157-83-0") OR TITLE-ABS-KEY("C29H38O4") OR TITLE-ABS-KEY("CDP571") OR TITLE-ABS-KEY("CDP 571") OR TITLE-ABS-KEY("CDP-571") OR TITLE-ABS-KEY("Afelimomab") OR TITLE-ABS-KEY("Segard") OR TITLE-ABS-KEY("MAK-195F") OR TITLE-ABS-KEY("MAK 195F") OR TITLE-ABS-KEY("MAK195F") OR TITLE-ABS-KEY("LU-54107") OR TITLE-ABS-KEY("LU 54107") OR TITLE-ABS-KEY("LU54107") OR TITLE-ABS-KEY("156227-98-4") OR TITLE-ABS-KEY("Camobucol") OR TITLE-ABS-KEY("ylgucpd100bdh") OR TITLE-ABS-KEY("V-protectant") OR TITLE-ABS-KEY("AGIX-4207") OR TITLE-ABS-KEY("AGIX 4207") OR TITLE-ABS-KEY("AGIX4207") OR TITLE-ABS-KEY("216167-92-9") OR TITLE-ABS-KEY("C33H50O4S2") OR TITLE-ABS-KEY("CRX-191") OR TITLE-ABS-KEY("CRX 191") OR TITLE-ABS-KEY("CRX191") OR TITLE-ABS-KEY("AME-527") OR TITLE-ABS-KEY("AME 527") OR TITLE-ABS-KEY("AME527") OR TITLE-ABS-KEY("1001159-86-9") OR TITLE-ABS-KEY("CYT-007-TNFQb") OR TITLE-ABS-KEY("CYT 007 TNFQb") OR TITLE-ABS-KEY("CYT007TNFQb") OR TITLE-ABS-KEY("ALS-00T2-0501") OR TITLE-ABS-KEY("ALS 00T2 0501") OR TITLE-ABS-KEY("ALS00T20501") OR TITLE-ABS-KEY("FR-133605") OR TITLE-ABS-KEY("FR 133605") OR TITLE-ABS-KEY("FR133605") OR TITLE-ABS-KEY("MDL-201112") OR TITLE-ABS-KEY("MDL 201112") OR TITLE-ABS-KEY("MDL201112") OR TITLE-ABS-KEY("MDL-201449") OR TITLE-ABS-KEY("MDL 201449") OR TITLE-ABS-KEY("MDL201449") OR TITLE-ABS-KEY("MDL-201449A") OR TITLE-ABS-KEY("MDL 201449A") OR TITLE-ABS-KEY("MDL201449A") OR TITLE-ABS-KEY("142130-73-2") OR TITLE-ABS-KEY("C10H13N5O") OR TITLE-ABS-KEY("Carbocyclic nucleoside")) AND (TITLE-ABS-KEY("Alopecia cicatrisata*") OR TITLE-ABS-KEY("Cicatricial alopeci*") OR TITLE-ABS-KEY("Scarring alopeci*") OR TITLE-ABS-KEY("Irreversible alopeci*") OR TITLE-ABS-KEY("Permanent alopeci*") OR TITLE-ABS-KEY("Cicatricial bald*") OR TITLE-ABS-KEY("Scarring bald*") OR TITLE-ABS-KEY("Irreversible bald*") OR TITLE-ABS-KEY("Permanent bald*") OR TITLE-ABS-KEY("Cicatricial hair los*") OR TITLE-ABS-KEY("Scarring hair los*") OR TITLE-ABS-KEY("Irreversible hair los*") OR TITLE-ABS-KEY("Permanent hair los*") OR TITLE-ABS-KEY("Pseudopelad*") OR TITLE-ABS-KEY("Brocq") OR TITLE-ABS-KEY("Scarring lichen plan*") OR TITLE-ABS-KEY("Scalp lichen plan*") OR TITLE-ABS-KEY("Lichen planopilar*") OR TITLE-ABS-KEY("Coup de sabre*") OR TITLE-ABS-KEY("Discoid lupus*") OR TITLE-ABS-KEY("Folliculitis decalvans") OR TITLE-ABS-KEY("Erosive pustular dermatosis") OR TITLE-ABS-KEY("Chronic atrophic dermatosis") OR TITLE-ABS-KEY("Frontal fibrosing alopecia") OR TITLE-ABS-KEY("Central centrifugal alopecia") OR TITLE-ABS-KEY("Central centrifugal cicatricial alopecia") OR TITLE-ABS-KEY("Tufted folliculitis") OR TITLE-ABS-KEY("Dissecting cellulitis") OR TITLE-ABS-KEY("Perifolliculitis capitis") OR TITLE-ABS-KEY("Folliculitis keloidalis") OR TITLE-ABS-KEY("Acne cheloidalis nuch*") OR TITLE-ABS-KEY("Acne keloidalis") OR TITLE-ABS-KEY("Papular atrichia") OR TITLE-ABS-KEY("Atrichia with papular lesion*")) | 587 |
| Web of Science | (TI=("*JAK inhibit*") OR TI=("*Jakinib*") OR TI=("*Janus kinase inhibit*") OR TI=("*Tyrosine kinase inhibit*") OR TI=("*JAK-STAT inhibit*") OR TI=("*JAKi*") OR TI=("*JAK i*") OR TI=("*JAK-i*") OR TI=("*JAK1 inhibit*") OR TI=("*JAK2 inhibit*") OR TI=("*JAK3 inhibit*") OR TI=("*TYK2 inhibit*") OR TI=("*Janus kinase 1 inhibit*") OR TI=("*Janus kinase 2 inhibit*") OR TI=("*Janus kinase 3 inhibit*") OR TI=("*Tyrosine kinase 2 inhibit*") OR TI=("Ruxolitinib") OR TI=("Jakafi") OR TI=("Jakavi") OR TI=("Opzelura") OR TI=("INCB018424") OR TI=("INCB-018424") OR TI=("INCB 018424") OR TI=("INCB18424") OR TI=("INCB-18424") OR TI=("INCB 18424") OR TI=("INCA24") OR TI=("INC-A24") OR TI=("INC A24") OR TI=("INC424") OR TI=("INC-424") OR TI=("INC 424") OR TI=("941678-49-5") OR TI=("C17H18N6") OR TI=("C17H21N6O4P") OR TI=("Tofacitinib") OR TI=("Tasocitinib") OR TI=("Xeljanz") OR TI=("Jaquinus") OR TI=("CP-690550") OR TI=("CP 690550") OR TI=("CP690550") OR TI=("CP-690,550") OR TI=("CP 690,550") OR TI=("CP690,550") OR TI=("477600-75-2") OR TI=("C16H20N6O") OR TI=("Oclacitinib") OR TI=("Apoquel") OR TI=("PF 03394197") OR TI=("PF-03394197") OR TI=("PF03394197") OR TI=("1208319-26-9") OR TI=("C15H23N5O2S") OR TI=("Baricitinib") OR TI=("Olumiant") OR TI=("INCB 028050") OR TI=("INCB-028050") OR TI=("INCB028050") OR TI=("INCB 28050") OR TI=("INCB-28050") OR TI=("INCB28050") OR TI=("LY 3009104") OR TI=("LY-3009104") OR TI=("LY3009104") OR TI=("1187594-09-7") OR TI=("C16H17N7O2S") OR TI=("Peficitinib") OR TI=("Smyraf") OR TI=("ASP 015K") OR TI=("ASP-015K") OR TI=("ASP015K") OR TI=("JNJ 54781532") OR TI=("JNJ-54781532") OR TI=("JNJ54781532") OR TI=("944118-01-8") OR TI=("C18H22N4O2") OR TI=("Upadacitinib") OR TI=("Rinvoq") OR TI=("ABT 494") OR TI=("ABT-494") OR TI=("ABT494") OR TI=("1310726-60-3") OR TI=("C17H19F3N6O") OR TI=("Fedratinib") OR TI=("Inrebic") OR TI=("SAR 302503") OR TI=("SAR-302503") OR TI=("SAR302503") OR TI=("TG 101348") OR TI=("TG-101348") OR TI=("TG101348") OR TI=("936091-26-8") OR TI=("C27H36N6O3S") OR TI=("Delgocitinib") OR TI=("Corectim") OR TI=("JTE 052") OR TI=("JTE-052") OR TI=("JTE052") OR TI=("JTE 052A") OR TI=("JTE-052A") OR TI=("JTE052A") OR TI=("LEO 124249") OR TI=("LEO-124249") OR TI=("LEO124249") OR TI=("LEO 124249A") OR TI=("LEO-124249A") OR TI=("LEO124249A") OR TI=("1263774-59-9") OR TI=("C16H18N6O") OR TI=("Filgotinib") OR TI=("Jyseleca") OR TI=("GS 6034") OR TI=("GS-6034") OR TI=("GS6034") OR TI=("GLPG 0634") OR TI=("GLPG-0634") OR TI=("GLPG0634") OR TI=("G 146034") OR TI=("G-146034") OR TI=("G146034") OR TI=("1206101-20-3") OR TI=("1206161-97-8") OR TI=("C21H23N5O3S") OR TI=("Abrocitinib") OR TI=("Cibinqo") OR TI=("PF 04965842") OR TI=("PF-04965842") OR TI=("PF04965842") OR TI=("1622902-68-4") OR TI=("C14H21N5O2S") OR TI=("Pacritinib") OR TI=("Vonjo") OR TI=("SB 1518") OR TI=("SB-1518") OR TI=("SB1518") OR TI=("937272-79-2") OR TI=("C28H32N4O3") OR TI=("Deucravacitinib") OR TI=("Sotyktu") OR TI=("BMS 986165") OR TI=("BMS-986165") OR TI=("BMS986165") OR TI=("Tyk2-IN-4") OR TI=("Tyk2 IN 4") OR TI=("Tyk2IN4") OR TI=("1609392-27-9") OR TI=("C20H22N8O3") OR TI=("Cerdulatinib") OR TI=("PRT 2070") OR TI=("PRT-2070") OR TI=("PRT2070") OR TI=("PRT 062070") OR TI=("PRT-062070") OR TI=("PRT062070") OR TI=("DMVT 502") OR TI=("DMVT-502") OR TI=("DMVT502") OR TI=("RVT 502") OR TI=("RVT-502") OR TI=("RVT502") OR TI=("1198300-79-6") OR TI=("C20H27N7O3S") OR TI=("Gandotinib") OR TI=("LY 2784544") OR TI=("LY-2784544") OR TI=("LY2784544") OR TI=("1229236-86-5") OR TI=("C23H25ClFN7O") OR TI=("Lestaurtinib") OR TI=("CEP 701") OR TI=("CEP-701") OR TI=("CEP701") OR TI=("KT 5555") OR TI=("KT-5555") OR TI=("KT5555") OR TI=("SPM 924") OR TI=("SPM-924") OR TI=("SPM924") OR TI=("KT 555") OR TI=("KT-555") OR TI=("KT555") OR TI=("111358-88-4") OR TI=("C26H21N3O4") OR TI=("Momelotinib") OR TI=("CYT 387") OR TI=("CYT-387") OR TI=("CYT387") OR TI=("CYT 11387") OR TI=("CYT-11387") OR TI=("CYT11387") OR TI=("GS-0387") OR TI=("GS 0387") OR TI=("GS0387") OR TI=("GS-387") OR TI=("GS 387") OR TI=("GS387") OR TI=("LM 1149") OR TI=("LM-1149") OR TI=("LM1149") OR TI=("1056634-68-4") OR TI=("Cucurbitacin") OR TI=("Elatericin") OR TI=("JSI 124") OR TI=("JSI-124") OR TI=("JSI124") OR TI=("NSC 521777") OR TI=("NSC-521777") OR TI=("NSC521777") OR TI=("NSC 112167") OR TI=("NSC-112167") OR TI=("NSC112167") OR TI=("2222-07-3") OR TI=("C30H42O7") OR TI=("CHZ 868") OR TI=("CHZ-868") OR TI=("CHZ868") OR TI=("C22H19F2N5O2") OR TI=("Tumor Necrosis Factor inhib*") OR TI=("Tumor Necrosis Factor-a inhib*") OR TI=("Tumor Necrosis Factor-α inhib*") OR TI=("Tumor Necrosis Factor-alpha inhib*") OR TI=("Tumor Necrosis Factor a inhib*") OR TI=("Tumor Necrosis Factor α inhib*") OR TI=("Tumor Necrosis Factor alpha inhib*") OR TI=("Tumor Necrosis Factor antag*") OR TI=("Tumor Necrosis Factor-a antag*") OR TI=("Tumor Necrosis Factor-α antag*") OR TI=("Tumor Necrosis Factor-alpha antag*") OR TI=("Tumor Necrosis Factor a antag*") OR TI=("Tumor Necrosis Factor α antag*") OR TI=("Tumor Necrosis Factor alpha antag*") OR TI=("Tumor Necrosis Factor block*") OR TI=("Tumor Necrosis Factor-a block*") OR TI=("Tumor Necrosis Factor-α block*") OR TI=("Tumor Necrosis Factor-alpha block*") OR TI=("Tumor Necrosis Factor a block*") OR TI=("Tumor Necrosis Factor α block*") OR TI=("Tumor Necrosis Factor alpha block*") OR TI=("Tumour Necrosis Factor inhib*") OR TI=("Tumour Necrosis Factor-a inhib*") OR TI=("Tumour Necrosis Factor-α inhib*") OR TI=("Tumour Necrosis Factor-alpha inhib*") OR TI=("Tumour Necrosis Factor a inhib*") OR TI=("Tumour Necrosis Factor α inhib*") OR TI=("Tumour Necrosis Factor alpha inhib*") OR TI=("Tumour Necrosis Factor antag*") OR TI=("Tumour Necrosis Factor-a antag*") OR TI=("Tumour Necrosis Factor-α antag*") OR TI=("Tumour Necrosis Factor-alpha antag*") OR TI=("Tumour Necrosis Factor a antag*") OR TI=("Tumour Necrosis Factor α antag*") OR TI=("Tumour Necrosis Factor alpha antag*") OR TI=("Tumour Necrosis Factor block*") OR TI=("Tumour Necrosis Factor-a block*") OR TI=("Tumour Necrosis Factor-α block*") OR TI=("Tumour Necrosis Factor-alpha block*") OR TI=("Tumour Necrosis Factor a block*") OR TI=("Tumour Necrosis Factor α block*") OR TI=("Tumour Necrosis Factor alpha block*") OR TI=("TNF inhib*") OR TI=("TNF-a inhib*") OR TI=("TNF-α inhib*") OR TI=("TNF-alpha inhib*") OR TI=("TNF a inhib*") OR TI=("TNF α inhib*") OR TI=("TNF alpha inhib*") OR TI=("TNF antag*") OR TI=("TNF-a antag*") OR TI=("TNF-α antag*") OR TI=("TNF-alpha antag*") OR TI=("TNF a antag*") OR TI=("TNF α antag*") OR TI=("TNF alpha antag*") OR TI=("TNF block*") OR TI=("TNF-a block*") OR TI=("TNF-α block*") OR TI=("TNF-alpha block*") OR TI=("TNF a block*") OR TI=("TNF α block*") OR TI=("TNF alpha block*") OR TI=("Anti-Tumour Necrosis Factor*") OR TI=("Anti-TNF*") OR TI=("Cachectin inhib*") OR TI=("Cachectin antag*") OR TI=("Cachectin block*") OR TI=("Infliximab") OR TI=("Remicade") OR TI=("Inflectra") OR TI=("Avsola") OR TI=("Ixifi") OR TI=("Renflexis") OR TI=("Mab cA2") OR TI=("Monoclonal antibody cA2") OR TI=("TA-650") OR TI=("TA 650") OR TI=("TA650") OR TI=("170277-31-3") OR TI=("Adalimumab") OR TI=("Humira") OR TI=("Abrilada") OR TI=("Amgevita") OR TI=("Amjevita") OR TI=("Amsparity") OR TI=("Ardalicip") OR TI=("Cadalimab") OR TI=("Ciptunec") OR TI=("Cyltezo") OR TI=("Exemptia") OR TI=("Hadlima") OR TI=("Halimatoz") OR TI=("Hefiya") OR TI=("Hukyndra") OR TI=("Hulio") OR TI=("Hyrimoz") OR TI=("Idacio") OR TI=("Imraldi") OR TI=("Kromeya") OR TI=("Libmyris") OR TI=("Mabura") OR TI=("Simlandi") OR TI=("Solymbic") OR TI=("Trudexa") OR TI=("Yuflyma") OR TI=("Yusimry") OR TI=("D2E7") OR TI=("331731-18-1") OR TI=("C6428H9912N1694O1987S46") OR TI=("Etanercept") OR TI=("Enbrel") OR TI=("Erelzi") OR TI=("Eticovo") OR TI=("Benepali") OR TI=("TNFR-Fc") OR TI=("TNFR Fc") OR TI=("TNT Receptor Fusion Protein") OR TI=("TNF Receptor Type II-IgG Fusion Protein") OR TI=("TNF Receptor Type II IgG Fusion Protein") OR TI=("TNR 001") OR TI=("TNR-001") OR TI=("TNR001") OR TI=("185243-69-0") OR TI=("C2224H3475N621O698S36") OR TI=("Golimumab") OR TI=("Simponi") OR TI=("CNTO-148") OR TI=("CNTO 148") OR TI=("CNTO148") OR TI=("476181-74-5") OR TI=("C6530H10068N1752O2026S44") OR TI=("Certolizumab") OR TI=("Cimzia") OR TI=("CDP 870") OR TI=("CDP-870") OR TI=("CDP870") OR TI=("428863-50-7") OR TI=("C2115H3252N556O673S16") OR TI=("Thalidomide") OR TI=("Thalomid*") OR TI=("Algosediv") OR TI=("Asmadion") OR TI=("Asmaval") OR TI=("Bonbrain") OR TI=("Calmore") OR TI=("Calmorex") OR TI=("Contergan") OR TI=("Corronarobetin") OR TI=("Distaval") OR TI=("Ectiluran") OR TI=("Enterosediv") OR TI=("Gastrinide") OR TI=("Glupan") OR TI=("Glutanon") OR TI=("Grippex") OR TI=("Hippuzon") OR TI=("Imidene") OR TI=("Isomin") OR TI=("Kevadon") OR TI=("Neosedyn") OR TI=("Nerosedyn") OR TI=("Neufatin") OR TI=("Neurodyn") OR TI=("Neurosedin") OR TI=("Neurosedym") OR TI=("Nevrodyn") OR TI=("Nibrol") OR TI=("Noctosediv") OR TI=("Noxodyn") OR TI=("Pangul") OR TI=("Pantosediv") OR TI=("Phthalimidoglut") OR TI=("Phthaloylglut") OR TI=("Phthalylglut") OR TI=("Polygripan") OR TI=("Profarmil") OR TI=("Psycholiquid") OR TI=("Psychotablet") OR TI=("Quetimid") OR TI=("Quietoplex") OR TI=("Sandormin") OR TI=("Sedimide") OR TI=("Sedisperil") OR TI=("Sedoval") OR TI=("Shinnibrol") OR TI=("Sleepan") OR TI=("Slipro") OR TI=("Softenil") OR TI=("Softenon") OR TI=("Talargan") OR TI=("Talimol") OR TI=("Talismol") OR TI=("Telagan") OR TI=("Telargean") OR TI=("Tensival") OR TI=("Thalin") OR TI=("Thalinette") OR TI=("Theophilcholine") OR TI=("Valgis") OR TI=("Valgraine") OR TI=("50-35-1") OR TI=("C13H10N2O4") OR TI=("Lenalidomide") OR TI=("Revlimid") OR TI=("Linamide") OR TI=("Revamid") OR TI=("Revimid") OR TI=("CC 5013") OR TI=("CC-5013") OR TI=("CC5013") OR TI=("CDC 501") OR TI=("CDC-501") OR TI=("CDC501") OR TI=("IMiD3") OR TI=("IMID-5013") OR TI=("IMID 5013") OR TI=("IMID5013") OR TI=("ENMD-0997") OR TI=("ENMD 0997") OR TI=("ENMD0997") OR TI=("191732-72-6") OR TI=("C13H13N3O3") OR TI=("Nafamostat") OR TI=("Simponi") OR TI=("Ronastat") OR TI=("CKD-314") OR TI=("CKD 314") OR TI=("CKD314") OR TI=("FUT-175") OR TI=("FUT 175") OR TI=("FUT175") OR TI=("81525-10-2") OR TI=("C19H17N5O2") OR TI=("Pentoxifylline") OR TI=("Oxpentifylline") OR TI=("Trental") OR TI=("Torental") OR TI=("Pentoxil") OR TI=("Dimethyloxohexylxanthine") OR TI=("Agapurin") OR TI=("Azupentat") OR TI=("Durapental") OR TI=("Hemovas") OR TI=("Ikomio") OR TI=("Pentoxifil") OR TI=("Ralofect") OR TI=("Rentylin") OR TI=("Vasofirin") OR TI=("Vazofirin") OR TI=("Pentoxin") OR TI=("BL 191") OR TI=("BL-191") OR TI=("BL191") OR TI=("6493-05-6") OR TI=("C13H18N4O3") OR TI=("ABP 501") OR TI=("ABP-501") OR TI=("ABP501") OR TI=("Ajulemic") OR TI=("Lenabasum") OR TI=("Resunab") OR TI=("Cervelo") OR TI=("Anabasum") OR TI=("IP-751") OR TI=("IP 751") OR TI=("IP751") OR TI=("CPL-7075") OR TI=("CPL 7075") OR TI=("CPL7075") OR TI=("CT-3") OR TI=("CT 3") OR TI=("CT3") OR TI=("137945-48-3") OR TI=("Golnerminogene") OR TI=("TNFerade") OR TI=("957472-14-9") OR TI=("Ad5FGF-4") OR TI=("Ad5FGF 4") OR TI=("Ad5FGF4") OR TI=("PF-06410293") OR TI=("PF 06410293") OR TI=("PF06410293") OR TI=("PF-06438179") OR TI=("PF 06438179") OR TI=("PF06438179") OR TI=("GP 1111") OR TI=("GP-1111") OR TI=("GP1111") OR TI=("Remtolumab") OR TI=("ABT-122") OR TI=("ABT 122") OR TI=("ABT122") OR TI=("1791410-27-9") OR TI=("AN0128") OR TI=("AN 0128") OR TI=("AN-0128") OR TI=("CRM-0005") OR TI=("CRM 0005") OR TI=("CRM0005") OR TI=("872044-70-7") OR TI=("Apeptico") OR TI=("AP-301") OR TI=("AP 301") OR TI=("AP301") OR TI=("Baicalein") OR TI=("Noroxylin") OR TI=("Baikalein") OR TI=("Baicelein") OR TI=("Baikelein") OR TI=("NSC661431") OR TI=("NSC 661431") OR TI=("NSC-661431") OR TI=("Sho-saiko-to") OR TI=("491-67-8") OR TI=("C15H10O5") OR TI=("Delenex") OR TI=("DLX-105") OR TI=("DLX 105") OR TI=("DLX105") OR TI=("ESBA-105") OR TI=("ESBA 105") OR TI=("ESBA105") OR TI=("1142149-71-0") OR TI=("Ortataxel") OR TI=("8H61Y4E29N") OR TI=("IDN-5109") OR TI=("IDN 5109") OR TI=("IDN5109") OR TI=("Bay-59*") OR TI=("Bay 59*") OR TI=("Bay59*") OR TI=("Hexanoic acid") OR TI=("Genz 29155") OR TI=("Genz-29155") OR TI=("Genz29155") OR TI=("SB-T-101131") OR TI=("SB T 101131") OR TI=("SBT101131") OR TI=("186348-23-2") OR TI=("C44H57NO17") OR TI=("Pegsunercept") OR TI=("PEGylated soluble tumor necrosis factor receptor I") OR TI=("PEG sTNFRI") OR TI=("STNF-RI") OR TI=("STNF RI") OR TI=("STNFRI") OR TI=("STNF-R1") OR TI=("STNF R1") OR TI=("STNFR1") OR TI=("330988-75-5") OR TI=("C502H758N154O165S16") OR TI=("Allotrap") OR TI=("Delmitide") OR TI=("Peptide Bc-1nl") OR TI=("RDP 58") OR TI=("RDP-58") OR TI=("RDP58") OR TI=("RSP 58") OR TI=("RSP-58") OR TI=("RSP58") OR TI=("287096-87-1") OR TI=("C59H105N17O11") OR TI=("Kinoid") OR TI=("Neovacs") OR TI=("Debio-0512") OR TI=("Debio 0512") OR TI=("Debio0512") OR TI=("COVA322") OR TI=("COVA-322") OR TI=("COVA 322") OR TI=("GTPL10469") OR TI=("GTPL 10469") OR TI=("GTPL-10469") OR TI=("Placulumab") OR TI=("PN-0621") OR TI=("PN 0621") OR TI=("PN0621") OR TI=("CEP-37247") OR TI=("CEP 37247") OR TI=("CEP37247") OR TI=("ART621") OR TI=("ART 621") OR TI=("ART-621") OR TI=("ABBV-257") OR TI=("ABBV 257") OR TI=("ABBV257") OR TI=("MA59O0RPES") OR TI=("AST-005") OR TI=("AST 005") OR TI=("AST005") OR TI=("AVX-470") OR TI=("AVX 470") OR TI=("AVX470") OR TI=("Aurimune") OR TI=("CYT-609") OR TI=("CYT 609") OR TI=("CYT609") OR TI=("877170-68-8") OR TI=("INB03") OR TI=("INB 03") OR TI=("INB-03") OR TI=("PF-05230905") OR TI=("PF 05230905") OR TI=("PF05230905") OR TI=("ATN-192") OR TI=("ATN 192") OR TI=("ATN192") OR TI=("PMI-005") OR TI=("PMI 005") OR TI=("PMI005") OR TI=("ABX-0401") OR TI=("ABX 0401") OR TI=("ABX0401") OR TI=("ALX-0071") OR TI=("ALX 0071") OR TI=("ALX0071") OR TI=("Celastrol") OR TI=("Tripterin") OR TI=("Celastrus scandens") OR TI=("34157-83-0") OR TI=("C29H38O4") OR TI=("CDP571") OR TI=("CDP 571") OR TI=("CDP-571") OR TI=("Afelimomab") OR TI=("Segard") OR TI=("MAK-195F") OR TI=("MAK 195F") OR TI=("MAK195F") OR TI=("LU-54107") OR TI=("LU 54107") OR TI=("LU54107") OR TI=("156227-98-4") OR TI=("Camobucol") OR TI=("ylgucpd100bdh") OR TI=("V-protectant") OR TI=("AGIX-4207") OR TI=("AGIX 4207") OR TI=("AGIX4207") OR TI=("216167-92-9") OR TI=("C33H50O4S2") OR TI=("CRX-191") OR TI=("CRX 191") OR TI=("CRX191") OR TI=("AME-527") OR TI=("AME 527") OR TI=("AME527") OR TI=("1001159-86-9") OR TI=("CYT-007-TNFQb") OR TI=("CYT 007 TNFQb") OR TI=("CYT007TNFQb") OR TI=("ALS-00T2-0501") OR TI=("ALS 00T2 0501") OR TI=("ALS00T20501") OR TI=("FR-133605") OR TI=("FR 133605") OR TI=("FR133605") OR TI=("MDL-201112") OR TI=("MDL 201112") OR TI=("MDL201112") OR TI=("MDL-201449") OR TI=("MDL 201449") OR TI=("MDL201449") OR TI=("MDL-201449A") OR TI=("MDL 201449A") OR TI=("MDL201449A") OR TI=("142130-73-2") OR TI=("C10H13N5O") OR TI=("Carbocyclic nucleoside") OR AB=("JAK inhibit*") OR AB=("Jakinib*") OR AB=("Janus kinase inhibit*") OR AB=("Tyrosine kinase inhibit*") OR AB=("JAK-STAT inhibit*") OR AB=("JAKi*") OR AB=("JAK i*") OR AB=("JAK-i*") OR AB=("JAK1 inhibit*") OR AB=("JAK2 inhibit*") OR AB=("JAK3 inhibit*") OR AB=("TYK2 inhibit*") OR AB=("Janus kinase 1 inhibit*") OR AB=("Janus kinase 2 inhibit*") OR AB=("Janus kinase 3 inhibit*") OR AB=("Tyrosine kinase 2 inhibit*") OR AB=("Ruxolitinib") OR AB=("Jakafi") OR AB=("Jakavi") OR AB=("Opzelura") OR AB=("INCB018424") OR AB=("INCB-018424") OR AB=("INCB 018424") OR AB=("INCB18424") OR AB=("INCB-18424") OR AB=("INCB 18424") OR AB=("INCA24") OR AB=("INC-A24") OR AB=("INC A24") OR AB=("INC424") OR AB=("INC-424") OR AB=("INC 424") OR AB=("941678-49-5") OR AB=("C17H18N6") OR AB=("C17H21N6O4P") OR AB=("Tofacitinib") OR AB=("Tasocitinib") OR AB=("Xeljanz") OR AB=("Jaquinus") OR AB=("CP-690550") OR AB=("CP 690550") OR AB=("CP690550") OR AB=("CP-690,550") OR AB=("CP 690,550") OR AB=("CP690,550") OR AB=("477600-75-2") OR AB=("C16H20N6O") OR AB=("Oclacitinib") OR AB=("Apoquel") OR AB=("PF 03394197") OR AB=("PF-03394197") OR AB=("PF03394197") OR AB=("1208319-26-9") OR AB=("C15H23N5O2S") OR AB=("Baricitinib") OR AB=("Olumiant") OR AB=("INCB 028050") OR AB=("INCB-028050") OR AB=("INCB028050") OR AB=("INCB 28050") OR AB=("INCB-28050") OR AB=("INCB28050") OR AB=("LY 3009104") OR AB=("LY-3009104") OR AB=("LY3009104") OR AB=("1187594-09-7") OR AB=("C16H17N7O2S") OR AB=("Peficitinib") OR AB=("Smyraf") OR AB=("ASP 015K") OR AB=("ASP-015K") OR AB=("ASP015K") OR AB=("JNJ 54781532") OR AB=("JNJ-54781532") OR AB=("JNJ54781532") OR AB=("944118-01-8") OR AB=("C18H22N4O2") OR AB=("Upadacitinib") OR AB=("Rinvoq") OR AB=("ABT 494") OR AB=("ABT-494") OR AB=("ABT494") OR AB=("1310726-60-3") OR AB=("C17H19F3N6O") OR AB=("Fedratinib") OR AB=("Inrebic") OR AB=("SAR 302503") OR AB=("SAR-302503") OR AB=("SAR302503") OR AB=("TG 101348") OR AB=("TG-101348") OR AB=("TG101348") OR AB=("936091-26-8") OR AB=("C27H36N6O3S") OR AB=("Delgocitinib") OR AB=("Corectim") OR AB=("JTE 052") OR AB=("JTE-052") OR AB=("JTE052") OR AB=("JTE 052A") OR AB=("JTE-052A") OR AB=("JTE052A") OR AB=("LEO 124249") OR AB=("LEO-124249") OR AB=("LEO124249") OR AB=("LEO 124249A") OR AB=("LEO-124249A") OR AB=("LEO124249A") OR AB=("1263774-59-9") OR AB=("C16H18N6O") OR AB=("Filgotinib") OR AB=("Jyseleca") OR AB=("GS 6034") OR AB=("GS-6034") OR AB=("GS6034") OR AB=("GLPG 0634") OR AB=("GLPG-0634") OR AB=("GLPG0634") OR AB=("G 146034") OR AB=("G-146034") OR AB=("G146034") OR AB=("1206101-20-3") OR AB=("1206161-97-8") OR AB=("C21H23N5O3S") OR AB=("Abrocitinib") OR AB=("Cibinqo") OR AB=("PF 04965842") OR AB=("PF-04965842") OR AB=("PF04965842") OR AB=("1622902-68-4") OR AB=("C14H21N5O2S") OR AB=("Pacritinib") OR AB=("Vonjo") OR AB=("SB 1518") OR AB=("SB-1518") OR AB=("SB1518") OR AB=("937272-79-2") OR AB=("C28H32N4O3") OR AB=("Deucravacitinib") OR AB=("Sotyktu") OR AB=("BMS 986165") OR AB=("BMS-986165") OR AB=("BMS986165") OR AB=("Tyk2-IN-4") OR AB=("Tyk2 IN 4") OR AB=("Tyk2IN4") OR AB=("1609392-27-9") OR AB=("C20H22N8O3") OR AB=("Cerdulatinib") OR AB=("PRT 2070") OR AB=("PRT-2070") OR AB=("PRT2070") OR AB=("PRT 062070") OR AB=("PRT-062070") OR AB=("PRT062070") OR AB=("DMVT 502") OR AB=("DMVT-502") OR AB=("DMVT502") OR AB=("RVT 502") OR AB=("RVT-502") OR AB=("RVT502") OR AB=("1198300-79-6") OR AB=("C20H27N7O3S") OR AB=("Gandotinib") OR AB=("LY 2784544") OR AB=("LY-2784544") OR AB=("LY2784544") OR AB=("1229236-86-5") OR AB=("C23H25ClFN7O") OR AB=("Lestaurtinib") OR AB=("CEP 701") OR AB=("CEP-701") OR AB=("CEP701") OR AB=("KT 5555") OR AB=("KT-5555") OR AB=("KT5555") OR AB=("SPM 924") OR AB=("SPM-924") OR AB=("SPM924") OR AB=("KT 555") OR AB=("KT-555") OR AB=("KT555") OR AB=("111358-88-4") OR AB=("C26H21N3O4") OR AB=("Momelotinib") OR AB=("CYT 387") OR AB=("CYT-387") OR AB=("CYT387") OR AB=("CYT 11387") OR AB=("CYT-11387") OR AB=("CYT11387") OR AB=("GS-0387") OR AB=("GS 0387") OR AB=("GS0387") OR AB=("GS-387") OR AB=("GS 387") OR AB=("GS387") OR AB=("LM 1149") OR AB=("LM-1149") OR AB=("LM1149") OR AB=("1056634-68-4") OR AB=("Cucurbitacin") OR AB=("Elatericin") OR AB=("JSI 124") OR AB=("JSI-124") OR AB=("JSI124") OR AB=("NSC 521777") OR AB=("NSC-521777") OR AB=("NSC521777") OR AB=("NSC 112167") OR AB=("NSC-112167") OR AB=("NSC112167") OR AB=("2222-07-3") OR AB=("C30H42O7") OR AB=("CHZ 868") OR AB=("CHZ-868") OR AB=("CHZ868") OR AB=("C22H19F2N5O2") OR AB=("Tumor Necrosis Factor inhib*") OR AB=("Tumor Necrosis Factor-a inhib*") OR AB=("Tumor Necrosis Factor-α inhib*") OR AB=("Tumor Necrosis Factor-alpha inhib*") OR AB=("Tumor Necrosis Factor a inhib*") OR AB=("Tumor Necrosis Factor α inhib*") OR AB=("Tumor Necrosis Factor alpha inhib*") OR AB=("Tumor Necrosis Factor antag*") OR AB=("Tumor Necrosis Factor-a antag*") OR AB=("Tumor Necrosis Factor-α antag*") OR AB=("Tumor Necrosis Factor-alpha antag*") OR AB=("Tumor Necrosis Factor a antag*") OR AB=("Tumor Necrosis Factor α antag*") OR AB=("Tumor Necrosis Factor alpha antag*") OR AB=("Tumor Necrosis Factor block*") OR AB=("Tumor Necrosis Factor-a block*") OR AB=("Tumor Necrosis Factor-α block*") OR AB=("Tumor Necrosis Factor-alpha block*") OR AB=("Tumor Necrosis Factor a block*") OR AB=("Tumor Necrosis Factor α block*") OR AB=("Tumor Necrosis Factor alpha block*") OR AB=("Tumour Necrosis Factor inhib*") OR AB=("Tumour Necrosis Factor-a inhib*") OR AB=("Tumour Necrosis Factor-α inhib*") OR AB=("Tumour Necrosis Factor-alpha inhib*") OR AB=("Tumour Necrosis Factor a inhib*") OR AB=("Tumour Necrosis Factor α inhib*") OR AB=("Tumour Necrosis Factor alpha inhib*") OR AB=("Tumour Necrosis Factor antag*") OR AB=("Tumour Necrosis Factor-a antag*") OR AB=("Tumour Necrosis Factor-α antag*") OR AB=("Tumour Necrosis Factor-alpha antag*") OR AB=("Tumour Necrosis Factor a antag*") OR AB=("Tumour Necrosis Factor α antag*") OR AB=("Tumour Necrosis Factor alpha antag*") OR AB=("Tumour Necrosis Factor block*") OR AB=("Tumour Necrosis Factor-a block*") OR AB=("Tumour Necrosis Factor-α block*") OR AB=("Tumour Necrosis Factor-alpha block*") OR AB=("Tumour Necrosis Factor a block*") OR AB=("Tumour Necrosis Factor α block*") OR AB=("Tumour Necrosis Factor alpha block*") OR AB=("TNF inhib*") OR AB=("TNF-a inhib*") OR AB=("TNF-α inhib*") OR AB=("TNF-alpha inhib*") OR AB=("TNF a inhib*") OR AB=("TNF α inhib*") OR AB=("TNF alpha inhib*") OR AB=("TNF antag*") OR AB=("TNF-a antag*") OR AB=("TNF-α antag*") OR AB=("TNF-alpha antag*") OR AB=("TNF a antag*") OR AB=("TNF α antag*") OR AB=("TNF alpha antag*") OR AB=("TNF block*") OR AB=("TNF-a block*") OR AB=("TNF-α block*") OR AB=("TNF-alpha block*") OR AB=("TNF a block*") OR AB=("TNF α block*") OR AB=("TNF alpha block*") OR AB=("Anti-Tumour Necrosis Factor*") OR AB=("Anti-TNF*") OR AB=("Cachectin inhib*") OR AB=("Cachectin antag*") OR AB=("Cachectin block*") OR AB=("Infliximab") OR AB=("Remicade") OR AB=("Inflectra") OR AB=("Avsola") OR AB=("Ixifi") OR AB=("Renflexis") OR AB=("Mab cA2") OR AB=("Monoclonal antibody cA2") OR AB=("TA-650") OR AB=("TA 650") OR AB=("TA650") OR AB=("170277-31-3") OR AB=("Adalimumab") OR AB=("Humira") OR AB=("Abrilada") OR AB=("Amgevita") OR AB=("Amjevita") OR AB=("Amsparity") OR AB=("Ardalicip") OR AB=("Cadalimab") OR AB=("Ciptunec") OR AB=("Cyltezo") OR AB=("Exemptia") OR AB=("Hadlima") OR AB=("Halimatoz") OR AB=("Hefiya") OR AB=("Hukyndra") OR AB=("Hulio") OR AB=("Hyrimoz") OR AB=("Idacio") OR AB=("Imraldi") OR AB=("Kromeya") OR AB=("Libmyris") OR AB=("Mabura") OR AB=("Simlandi") OR AB=("Solymbic") OR AB=("Trudexa") OR AB=("Yuflyma") OR AB=("Yusimry") OR AB=("D2E7") OR AB=("331731-18-1") OR AB=("C6428H9912N1694O1987S46") OR AB=("Etanercept") OR AB=("Enbrel") OR AB=("Erelzi") OR AB=("Eticovo") OR AB=("Benepali") OR AB=("TNFR-Fc") OR AB=("TNFR Fc") OR AB=("TNT Receptor Fusion Protein") OR AB=("TNF Receptor Type II-IgG Fusion Protein") OR AB=("TNF Receptor Type II IgG Fusion Protein") OR AB=("TNR 001") OR AB=("TNR-001") OR AB=("TNR001") OR AB=("185243-69-0") OR AB=("C2224H3475N621O698S36") OR AB=("Golimumab") OR AB=("Simponi") OR AB=("CNTO-148") OR AB=("CNTO 148") OR AB=("CNTO148") OR AB=("476181-74-5") OR AB=("C6530H10068N1752O2026S44") OR AB=("Certolizumab") OR AB=("Cimzia") OR AB=("CDP 870") OR AB=("CDP-870") OR AB=("CDP870") OR AB=("428863-50-7") OR AB=("C2115H3252N556O673S16") OR AB=("Thalidomide") OR AB=("Thalomid*") OR AB=("Algosediv") OR AB=("Asmadion") OR AB=("Asmaval") OR AB=("Bonbrain") OR AB=("Calmore") OR AB=("Calmorex") OR AB=("Contergan") OR AB=("Corronarobetin") OR AB=("Distaval") OR AB=("Ectiluran") OR AB=("Enterosediv") OR AB=("Gastrinide") OR AB=("Glupan") OR AB=("Glutanon") OR AB=("Grippex") OR AB=("Hippuzon") OR AB=("Imidene") OR AB=("Isomin") OR AB=("Kevadon") OR AB=("Neosedyn") OR AB=("Nerosedyn") OR AB=("Neufatin") OR AB=("Neurodyn") OR AB=("Neurosedin") OR AB=("Neurosedym") OR AB=("Nevrodyn") OR AB=("Nibrol") OR AB=("Noctosediv") OR AB=("Noxodyn") OR AB=("Pangul") OR AB=("Pantosediv") OR AB=("Phthalimidoglut") OR AB=("Phthaloylglut") OR AB=("Phthalylglut") OR AB=("Polygripan") OR AB=("Profarmil") OR AB=("Psycholiquid") OR AB=("Psychotablet") OR AB=("Quetimid") OR AB=("Quietoplex") OR AB=("Sandormin") OR AB=("Sedimide") OR AB=("Sedisperil") OR AB=("Sedoval") OR AB=("Shinnibrol") OR AB=("Sleepan") OR AB=("Slipro") OR AB=("Softenil") OR AB=("Softenon") OR AB=("Talargan") OR AB=("Talimol") OR AB=("Talismol") OR AB=("Telagan") OR AB=("Telargean") OR AB=("Tensival") OR AB=("Thalin") OR AB=("Thalinette") OR AB=("Theophilcholine") OR AB=("Valgis") OR AB=("Valgraine") OR AB=("50-35-1") OR AB=("C13H10N2O4") OR AB=("Lenalidomide") OR AB=("Revlimid") OR AB=("Linamide") OR AB=("Revamid") OR AB=("Revimid") OR AB=("CC 5013") OR AB=("CC-5013") OR AB=("CC5013") OR AB=("CDC 501") OR AB=("CDC-501") OR AB=("CDC501") OR AB=("IMiD3") OR AB=("IMID-5013") OR AB=("IMID 5013") OR AB=("IMID5013") OR AB=("ENMD-0997") OR AB=("ENMD 0997") OR AB=("ENMD0997") OR AB=("191732-72-6") OR AB=("C13H13N3O3") OR AB=("Nafamostat") OR AB=("Simponi") OR AB=("Ronastat") OR AB=("CKD-314") OR AB=("CKD 314") OR AB=("CKD314") OR AB=("FUT-175") OR AB=("FUT 175") OR AB=("FUT175") OR AB=("81525-10-2") OR AB=("C19H17N5O2") OR AB=("Pentoxifylline") OR AB=("Oxpentifylline") OR AB=("Trental") OR AB=("Torental") OR AB=("Pentoxil") OR AB=("Dimethyloxohexylxanthine") OR AB=("Agapurin") OR AB=("Azupentat") OR AB=("Durapental") OR AB=("Hemovas") OR AB=("Ikomio") OR AB=("Pentoxifil") OR AB=("Ralofect") OR AB=("Rentylin") OR AB=("Vasofirin") OR AB=("Vazofirin") OR AB=("Pentoxin") OR AB=("BL 191") OR AB=("BL-191") OR AB=("BL191") OR AB=("6493-05-6") OR AB=("C13H18N4O3") OR AB=("ABP 501") OR AB=("ABP-501") OR AB=("ABP501") OR AB=("Ajulemic") OR AB=("Lenabasum") OR AB=("Resunab") OR AB=("Cervelo") OR AB=("Anabasum") OR AB=("IP-751") OR AB=("IP 751") OR AB=("IP751") OR AB=("CPL-7075") OR AB=("CPL 7075") OR AB=("CPL7075") OR AB=("CT-3") OR AB=("CT 3") OR AB=("CT3") OR AB=("137945-48-3") OR AB=("Golnerminogene") OR AB=("TNFerade") OR AB=("957472-14-9") OR AB=("Ad5FGF-4") OR AB=("Ad5FGF 4") OR AB=("Ad5FGF4") OR AB=("PF-06410293") OR AB=("PF 06410293") OR AB=("PF06410293") OR AB=("PF-06438179") OR AB=("PF 06438179") OR AB=("PF06438179") OR AB=("GP 1111") OR AB=("GP-1111") OR AB=("GP1111") OR AB=("Remtolumab") OR AB=("ABT-122") OR AB=("ABT 122") OR AB=("ABT122") OR AB=("1791410-27-9") OR AB=("AN0128") OR AB=("AN 0128") OR AB=("AN-0128") OR AB=("CRM-0005") OR AB=("CRM 0005") OR AB=("CRM0005") OR AB=("872044-70-7") OR AB=("Apeptico") OR AB=("AP-301") OR AB=("AP 301") OR AB=("AP301") OR AB=("Baicalein") OR AB=("Noroxylin") OR AB=("Baikalein") OR AB=("Baicelein") OR AB=("Baikelein") OR AB=("NSC661431") OR AB=("NSC 661431") OR AB=("NSC-661431") OR AB=("Sho-saiko-to") OR AB=("491-67-8") OR AB=("C15H10O5") OR AB=("Delenex") OR AB=("DLX-105") OR AB=("DLX 105") OR AB=("DLX105") OR AB=("ESBA-105") OR AB=("ESBA 105") OR AB=("ESBA105") OR AB=("1142149-71-0") OR AB=("Ortataxel") OR AB=("8H61Y4E29N") OR AB=("IDN-5109") OR AB=("IDN 5109") OR AB=("IDN5109") OR AB=("Bay-59*") OR AB=("Bay 59*") OR AB=("Bay59*") OR AB=("Hexanoic acid") OR AB=("Genz 29155") OR AB=("Genz-29155") OR AB=("Genz29155") OR AB=("SB-T-101131") OR AB=("SB T 101131") OR AB=("SBT101131") OR AB=("186348-23-2") OR AB=("C44H57NO17") OR AB=("Pegsunercept") OR AB=("PEGylated soluble tumor necrosis factor receptor I") OR AB=("PEG sTNFRI") OR AB=("STNF-RI") OR AB=("STNF RI") OR AB=("STNFRI") OR AB=("STNF-R1") OR AB=("STNF R1") OR AB=("STNFR1") OR AB=("330988-75-5") OR AB=("C502H758N154O165S16") OR AB=("Allotrap") OR AB=("Delmitide") OR AB=("Peptide Bc-1nl") OR AB=("RDP 58") OR AB=("RDP-58") OR AB=("RDP58") OR AB=("RSP 58") OR AB=("RSP-58") OR AB=("RSP58") OR AB=("287096-87-1") OR AB=("C59H105N17O11") OR AB=("Kinoid") OR AB=("Neovacs") OR AB=("Debio-0512") OR AB=("Debio 0512") OR AB=("Debio0512") OR AB=("COVA322") OR AB=("COVA-322") OR AB=("COVA 322") OR AB=("GTPL10469") OR AB=("GTPL 10469") OR AB=("GTPL-10469") OR AB=("Placulumab") OR AB=("PN-0621") OR AB=("PN 0621") OR AB=("PN0621") OR AB=("CEP-37247") OR AB=("CEP 37247") OR AB=("CEP37247") OR AB=("ART621") OR AB=("ART 621") OR AB=("ART-621") OR AB=("ABBV-257") OR AB=("ABBV 257") OR AB=("ABBV257") OR AB=("MA59O0RPES") OR AB=("AST-005") OR AB=("AST 005") OR AB=("AST005") OR AB=("AVX-470") OR AB=("AVX 470") OR AB=("AVX470") OR AB=("Aurimune") OR AB=("CYT-609") OR AB=("CYT 609") OR AB=("CYT609") OR AB=("877170-68-8") OR AB=("INB03") OR AB=("INB 03") OR AB=("INB-03") OR AB=("PF-05230905") OR AB=("PF 05230905") OR AB=("PF05230905") OR AB=("ATN-192") OR AB=("ATN 192") OR AB=("ATN192") OR AB=("PMI-005") OR AB=("PMI 005") OR AB=("PMI005") OR AB=("ABX-0401") OR AB=("ABX 0401") OR AB=("ABX0401") OR AB=("ALX-0071") OR AB=("ALX 0071") OR AB=("ALX0071") OR AB=("Celastrol") OR AB=("Tripterin") OR AB=("Celastrus scandens") OR AB=("34157-83-0") OR AB=("C29H38O4") OR AB=("CDP571") OR AB=("CDP 571") OR AB=("CDP-571") OR AB=("Afelimomab") OR AB=("Segard") OR AB=("MAK-195F") OR AB=("MAK 195F") OR AB=("MAK195F") OR AB=("LU-54107") OR AB=("LU 54107") OR AB=("LU54107") OR AB=("156227-98-4") OR AB=("Camobucol") OR AB=("ylgucpd100bdh") OR AB=("V-protectant") OR AB=("AGIX-4207") OR AB=("AGIX 4207") OR AB=("AGIX4207") OR AB=("216167-92-9") OR AB=("C33H50O4S2") OR AB=("CRX-191") OR AB=("CRX 191") OR AB=("CRX191") OR AB=("AME-527") OR AB=("AME 527") OR AB=("AME527") OR AB=("1001159-86-9") OR AB=("CYT-007-TNFQb") OR AB=("CYT 007 TNFQb") OR AB=("CYT007TNFQb") OR AB=("ALS-00T2-0501") OR AB=("ALS 00T2 0501") OR AB=("ALS00T20501") OR AB=("FR-133605") OR AB=("FR 133605") OR AB=("FR133605") OR AB=("MDL-201112") OR AB=("MDL 201112") OR AB=("MDL201112") OR AB=("MDL-201449") OR AB=("MDL 201449") OR AB=("MDL201449") OR AB=("MDL-201449A") OR AB=("MDL 201449A") OR AB=("MDL201449A") OR AB=("142130-73-2") OR AB=("C10H13N5O") OR AB=("Carbocyclic nucleoside")) AND (TI=("Alopecia cicatrisata*") OR TI=("Cicatricial alopeci*") OR TI=("Scarring alopeci*") OR TI=("Irreversible alopeci*") OR TI=("Permanent alopeci*") OR TI=("Cicatricial bald*") OR TI=("Scarring bald*") OR TI=("Irreversible bald*") OR TI=("Permanent bald*") OR TI=("Cicatricial hair los*") OR TI=("Scarring hair los*") OR TI=("Irreversible hair los*") OR TI=("Permanent hair los*") OR TI=("Pseudopelad*") OR TI=("Brocq") OR TI=("Scarring lichen plan*") OR TI=("Scalp lichen plan*") OR TI=("Lichen planopilar*") OR TI=("Coup de sabre*") OR TI=("Discoid lupus*") OR TI=("Folliculitis decalvans") OR TI=("Erosive pustular dermatosis") OR TI=("Chronic atrophic dermatosis") OR TI=("Frontal fibrosing alopecia") OR TI=("Central centrifugal alopecia") OR TI=("Central centrifugal cicatricial alopecia") OR TI=("Tufted folliculitis") OR TI=("Dissecting cellulitis") OR TI=("Perifolliculitis capitis") OR TI=("Folliculitis keloidalis") OR TI=("Acne cheloidalis nuch*") OR TI=("Acne keloidalis") OR TI=("Papular atrichia") OR TI=("Atrichia with papular lesion*") OR AB=("Alopecia cicatrisata*") OR AB=("Cicatricial alopeci*") OR AB=("Scarring alopeci*") OR AB=("Irreversible alopeci*") OR AB=("Permanent alopeci*") OR AB=("Cicatricial bald*") OR AB=("Scarring bald*") OR AB=("Irreversible bald*") OR AB=("Permanent bald*") OR AB=("Cicatricial hair los*") OR AB=("Scarring hair los*") OR AB=("Irreversible hair los*") OR AB=("Permanent hair los*") OR AB=("Pseudopelad*") OR AB=("Brocq") OR AB=("Scarring lichen plan*") OR AB=("Scalp lichen plan*") OR AB=("Lichen planopilar*") OR AB=("Coup de sabre*") OR AB=("Discoid lupus*") OR AB=("Folliculitis decalvans") OR AB=("Erosive pustular dermatosis") OR AB=("Chronic atrophic dermatosis") OR AB=("Frontal fibrosing alopecia") OR AB=("Central centrifugal alopecia") OR AB=("Central centrifugal cicatricial alopecia") OR AB=("Tufted folliculitis") OR AB=("Dissecting cellulitis") OR AB=("Perifolliculitis capitis") OR AB=("Folliculitis keloidalis") OR AB=("Acne cheloidalis nuch*") OR AB=("Acne keloidalis") OR AB=("Papular atrichia") OR AB=("Atrichia with papular lesion*")) | 147 |
| WHO ICTRP | In the title:  (Alopecia cicatrisata OR Cicatricial alopecia OR Scarring alopecia OR Lichen planopilaris OR Frontal fibrosing alopecia OR Pseudopelade OR Scalp lichen planus OR Discoid lupus OR Coup de sabre OR Folliculitis decalvans OR Erosive pustular dermatosis OR Chronic atrophic dermatosis OR Central centrifugal OR Tufted folliculitis OR Dissecting cellulitis OR Perifolliculitis capitis OR Folliculitis keloidalis OR Acne cheloidalis nuchae OR Acne keloidalis OR Papular atrichia OR Atrichia with papular lesions) AND (Janus Kinase Inhibit* OR JAK inhibit* OR Jakinib OR Ruxolitinib OR Tofacitinib OR Tasocitinib OR Oclacitinib OR Baricitinib OR Peficitinib OR Upadacitinib OR Fedratinib OR Delgocitinib OR Filgotinib OR Abrocitinib OR Pacritinib OR Deucravacitinib OR Cerdulatinib OR Gandotinib OR Lestaurtinib OR Momelotinib OR Cucurbitacin OR CHZ868 OR Tumor Necrosis Factor* OR TNF* OR Cachectin OR Infliximab OR Adalimumab OR Etanercept OR Golimumab OR Certolizumab OR Thalidomide OR Lenalidomide OR Nafamostat OR Pentoxifylline OR ABP-501 OR Ajulemic OR IP-751 OR Golnerminogene OR PF-06410293 OR PF-06438179 OR Remtolumab OR ABT-122 OR AN-0128 OR Apeptico OR AP-301 OR Baicalein OR Delenex OR DLX-105 OR Ortataxel OR Pegsunercept OR Allotrap OR Delmitide OR Kinoid OR Debio-0512 OR COVA322 OR GTPL10469 OR Placulumab OR ABBV-257 OR AST-005 OR AVX-470 OR Aurimune OR CYT-609 OR INB03 OR PF-05230905 OR PMI-005 OR ABX-0401 OR ALX-0071 OR Celastrol OR CDP571 OR Afelimomab OR Camobucol OR CRX-191 OR AME-527 OR CYT-007-TNFQb OR ALS-00T2-0501 OR FR-133605 OR MDL-201112 OR MDL-201449 OR MDL-201449A) | 5 |
