# Supplementary material for: Tumor necrosis factor inhibitors and janus kinase inhibitors in the treatment of cicatricial alopecia: A systematic review
Source: PLoS One. 2024 Feb 9;19(2):e0293433. doi: 10.1371/journal.pone.0293433 (PMC10857607; doi:10.1371/journal.pone.0293433)
Supplement: S5 Table — (DOCX) [file pone.0293433.s005.docx]

**S5 Table.** The quality assessment included before-after studies with no control group [21].

| NIH Quality Assessment Tool for Before-After (Pre-Post) Studies with No Control Group | | | | | | | | | | | | | |
| --- | --- | --- | --- | --- | --- | --- | --- | --- | --- | --- | --- | --- | --- |
| Study ID | Q1 | Q2 | Q3 | Q4 | Q5 | Q6 | Q7 | Q8 | Q9 | Q10 | Q11 | Q12 | Total Quality Score |
| Jouanique, 2004 | ✓ | ✗ | CD | NR | CD | ✓ | ✓ | ✗ | ✗ | ✓ | ✓ | NA | 5 (Poor) |
| Knop, 1983 | ✓ | ✓ | ✓ | ✓ | ✓ | ✓ | CD | ✗ | CD | ✓ | ✓ | NA | 8 (Fair) |
| Sallee, 2018 | ✓ | ✗ | CD | CD | CD | ✓ | CD | ✗ | ✓ | ✓ | CD | NA | 4 (Poor) |

*Q1: Was study question or objective clearly stated?, Q2: Were eligibility/selection criteria for the study population prespecified and clearly described?, Q3: Were the participants in the study representative of those who would be eligible for the test/service/intervention in the general or clinical population of interest?, Q4: Were all eligible participants that met the prespecified entry criteria enrolled?, Q5: Was the sample size sufficiently large to provide confidence in the findings?, Q6: Was the test/service/intervention clearly described and delivered consistently across the study population?, Q7: Were the outcome measures prespecified, clearly defined, valid, reliable, and assessed consistently across all study participants?, Q8: Were the people assessing the outcomes blinded to the participants' exposures/interventions?, Q9: Was the loss to follow-up after baseline 20% or less? Were those lost to follow-up accounted for in the analysis?, Q10: Did the statistical methods examine changes in outcome measures from before to after the intervention? Were statistical tests done that provided p values for the pre-to-post changes?, Q11: Were outcome measures of interest taken multiple times before the intervention and multiple times after the intervention (i.e., did they use an interrupted time-series design)?, Q12: If the intervention was conducted at a group level (e.g., a whole hospital, a community, etc.) did the statistical analysis take into account the use of individual-level data to determine effects at the group level?, Abbreviations: CD, cannot determine; NA, not applicable; NR, not reported*
