# Supplementary material for: Tumor necrosis factor inhibitors and janus kinase inhibitors in the treatment of cicatricial alopecia: A systematic review
Source: PLoS One. 2024 Feb 9;19(2):e0293433. doi: 10.1371/journal.pone.0293433 (PMC10857607; doi:10.1371/journal.pone.0293433)
Supplement: S6 Table — (DOCX) [file pone.0293433.s006.docx]

**S6 Table.** The quality assessment included case reports and case series by Murad et al. [22].

| Murad MH, et al. Quality Assessment Tool for Case Reports and Case Series | | | | | | | | | |
| --- | --- | --- | --- | --- | --- | --- | --- | --- | --- |
| Study ID | Selection | Ascertainment | | Causality | | | | Reporting | Total Quality Score |
|  | Q1 | Q2 | Q3 | Q4 | Q5 | Q6 | Q7 | Q8 |  |
| Abbasi, 2009 | ✓ | ✓ | CD | CD | ✗ | ✗ | ✓ | ✓ | 4 |
| Alam, 2020 | ✓ | ✓ | CD | CD | ✗ | CD | ✓ | ✓ | 4 |
| Alfadley, 2003 | ✓ | ✓ | CD | CD | ✗ | ✓ | ✓ | ✓ | 5 |
| Alhameedy, 2019 | ✓ | ✓ | CD | NR | ✓ | ✗ | CD | ✓ | 4 |
| Alsantali, 2021 | ✓ | ✓ | CD | ✓ | ✗ | CD | ✓ | ✓ | 5 |
| Amschler, 2018 | ✓ | ✓ | CD | CD | ✓ | ✗ | ✓ | ✓ | 5 |
| Batra, 2020 | ✓ | ✓ | CD | CD | ✗ | ✗ | ✓ | ✓ | 4 |
| Bordone, 2017 | ✓ | ✓ | ✓ | NR | ✓ | ✓ | ✓ | ✓ | 6 |
| Brandt, 2008 | ✓ | ✓ | CD | NR | ✗ | ✗ | ✓ | ✓ | 4 |
| Brehon, 2020 | ✓ | ✓ | ✓ | CD | ✓ | ✗ | ✓ | ✓ | 6 |
| Cautela, 2020 | ✓ | ✓ | CD | NR | ✗ | ✗ | ✓ | ✓ | 4 |
| El Shabrawi-Caelen, 2010 | ✓ | ✓ | CD | ✓ | ✓ | ✗ | ✓ | ✓ | 6 |
| Eldik, 2019 | ✓ | ✓ | CD | NR | ✗ | ✗ | CD | ✓ | 3 |
| Fässler, 2020 | ✓ | ✗ | CD | ✓ | ✓ | NR | ✓ | ✓ | 5 |
| Fernández-Torres, 2010 | ✓ | ✓ | ✓ | CD | ✓ | ✓ | NR | ✓ | 6 |
| Garbelini-Lima, 2021 | ✓ | ✓ | CD | ✗ | ✓ | ✗ | ✓ | ✓ | 5 |
| Garcovich, 2008 | ✓ | ✓ | ✓ | ✓ | ✓ | ✗ | NR | ✓ | 6 |
| George, 2001 | ✓ | ✓ | CD | ✓ | ✗ | CD | ✓ | ✓ | 5 |
| Helm, 2018 | ✓ | ✗ | CD | CD | ✗ | ✗ | NR | ✓ | 2 |
| Hession, 2010 | ✓ | ✓ | CD | NR | ✓ | ✗ | ✓ | ✓ | 5 |
| Hoy, 2022 | ✓ | ✓ | CD | CD | ✓ | ✗ | ✓ | ✓ | 5 |
| Iorizzo, 2022 | ✓ | ✓ | CD | ✓ | ✗ | ✗ | ✓ | ✓ | 5 |
| Jayasekera, 2016 | ✓ | ✓ | CD | ✓ | ✗ | ✗ | ✓ | ✓ | 5 |
| Jerjen, 2020 | ✓ | ✓ | CD | ✗ | ✓ | CD | ✓ | ✓ | 5 |
| Kreutzer, 2014 | ✓ | ✓ | CD | NR | ✓ | ✗ | ✓ | ✓ | 5 |
| Kurokawa, 2021 | ✓ | ✓ | CD | NR | ✗ | ✗ | ✓ | ✓ | 4 |
| Lenzy, 2010 | ✓ | ✗ | CD | NR | ✗ | ✗ | NR | ✓ | 2 |
| Leung, 2019 | ✓ | ✓ | CD | NR | ✗ | ✗ | CD | ✓ | 3 |
| Malara, 2022 | ✓ | ✓ | CD | NR | ✗ | ✗ | ✓ | ✓ | 4 |
| Mansouri, 2016 | ✓ | ✓ | CD | NR | ✗ | ✗ | ✓ | ✓ | 4 |
| Martín-García, 2015 | ✓ | ✓ | CD | NR | ✗ | ✗ | ✓ | ✓ | 4 |
| Masnec, 2018 | ✓ | ✓ | CD | NR | ✗ | ✗ | ✓ | ✓ | 4 |
| Maxon, 2020 | ✓ | ✓ | CD | CD | ✗ | ✗ | ✓ | ✓ | 4 |
| McPhie, 2020 | ✓ | ✓ | CD | CD | ✓ | ✗ | ✓ | ✓ | 5 |
| Mihaljević , 2012 | ✓ | CD | CD | CD | ✗ | ✗ | ✓ | ✓ | 3 |
| Minakawa, 2021 | ✓ | ✗ | CD | NR | ✗ | CD | ✓ | ✓ | 3 |
| Moussa, 2022 | ✓ | ✓ | CD | CD | ✗ | ✗ | ✓ | ✓ | 4 |
| Navarini, 2010 | ✓ | ✓ | CD | NR | ✓ | ✗ | ✓ | ✓ | 5 |
| Plante, 2020 | ✓ | ✓ | CD | CD | ✗ | ✓ | ✓ | ✓ | 5 |
| Sanchez-Diaz, 2021 | ✓ | ✓ | CD | ✗ | ✓ | ✓ | ✓ | ✓ | 6 |
| Shah, 2009 | ✓ | ✓ | ✓ | ✗ | ✗ | ✗ | ✓ | ✓ | 5 |
| Shireen, 2018 | ✓ | ✓ | CD | ✓ | ✗ | ✓ | ✓ | ✓ | 6 |
| Sjerobabski Masnec, 2018 | ✓ | ✓ | CD | NR | ✗ | ✗ | ✓ | ✓ | 4 |
| Stevens, 1997 | ✓ | ✓ | ✓ | CD | ✓ | ✓ | CD | ✓ | 6 |
| Sukhatme, 2009 | ✓ | ✓ | CD | ✓ | ✗ | ✗ | ✓ | ✓ | 5 |
| Swale, 2003 | ✓ | ✓ | ✓ | CD | ✓ | ✗ | ✓ | ✓ | 6 |
| Takahashi, 2019 | ✓ | ✓ | CD | NR | ✗ | ✓ | ✓ | ✓ | 5 |
| Tran, 2020 | ✓ | ✓ | CD | CD | ✗ | ✗ | ✓ | ✓ | 4 |
| Udkoff, 2016 | ✓ | ✓ | CD | CD | ✓ | ✗ | ✓ | ✓ | 5 |
| Walsh, 2013 | ✓ | ✓ | CD | CD | ✗ | ✗ | CD | ✓ | 3 |
| Wollina, 2012 | ✓ | ✓ | CD | CD | ✓ | ✗ | ✓ | ✓ | 5 |
| Wu, 2013 | ✓ | ✓ | ✗ | ✗ | ✗ | ✗ | ✓ | ✗ | 3 |
| Yang, 2017 | ✓ | ✓ | CD | CD | ✓ | ✓ | ✓ | ✓ | 6 |

*Q1. Does the patient[s] represent[s] the whole experience of the investigator (center) or is the selection method unclear to the extent that other patients with similar presentation may not have been reported?, Q2. Was the exposure adequately ascertained?, Q3. Was the outcome adequately ascertained?, Q4. Were other alternative causes that may explain the observation ruled out?, Q5. Was there a challenge/rechallenge phenomenon?, Q6. Was there a dose-response effect? (Questions 4-6 are mostly relevant to cases of adverse drug events), Q7. Was follow-up long enough for outcomes to occur?, Q8. Is the case[s] described with sufficient details to allow other investigators to replicate the research or to allow practitioners make inferences related to their own practice?, Abbreviations: CD, cannot determine; NA, not applicable; NR, not reported*
